# The crystal structure of *Erwinia amylovora* AmyR, a member of the YbjN protein family, shows similarity to type III secretion chaperones but suggests different cellular functions

**DOI:** 10.1371/journal.pone.0176049

**Published:** 2017-04-20

**Authors:** Joseph D. Bartho, Dom Bellini, Jochen Wuerges, Nicola Demitri, Mirco Toccafondi, Armin O. Schmitt, Youfu Zhao, Martin A. Walsh, Stefano Benini

**Affiliations:** 1 Bioorganic Chemistry and Bio-Crystallography laboratory (B2Cl), Faculty of Science and Technology, Free University of Bolzano, Piazza Università 5, Bolzano, Italy; 2 Diamond Light Source, Harwell Science and Innovation Campus, Didcot, United Kingdom; 3 Research Complex at Harwell, Harwell Science and Innovation Campus, Didcot, United Kingdom; 4 Elettra–Sincrotrone Trieste, S.S 14 km 163.5 in Area Science Park, Basovizza, Trieste, Italy; 5 Georg-August-Universität Göttingen, Dept. Nutztierwissenschaften, Breeding informatics, Margarethe von Wrangell-Weg 7, Göttingen, Germany; 6 Department of Crop Sciences, University of Illinois, 1201 W. Gregory Dr., Urbana, IL, United States of America; Centre National de la Recherche Scientifique, Aix-Marseille Université, FRANCE

## Abstract

AmyR is a stress and virulence associated protein from the plant pathogenic Enterobacteriaceae species *Erwinia amylovora*, and is a functionally conserved ortholog of YbjN from *Escherichia coli*. The crystal structure of *E*. *amylovora* AmyR reveals a class I type III secretion chaperone-like fold, despite the lack of sequence similarity between these two classes of protein and lacking any evidence of a secretion-associated role. The results indicate that AmyR, and YbjN proteins in general, function through protein-protein interactions without any enzymatic action. The YbjN proteins of Enterobacteriaceae show remarkably low sequence similarity with other members of the YbjN protein family in Eubacteria, yet a high level of structural conservation is observed. Across the YbjN protein family sequence conservation is limited to residues stabilising the protein core and dimerization interface, while interacting regions are only conserved between closely related species. This study presents the first structure of a YbjN protein from Enterobacteriaceae, the most highly divergent and well-studied subgroup of YbjN proteins, and an in-depth sequence and structural analysis of this important but poorly understood protein family.

## Introduction

*Erwinia amylovora* is a Gram-negative plant pathogen that belongs to the Enterobacteriaceae family, which is closely related to other pathogenic Enterobacteria such as *Escherichia coli*, *Yersinia pestis*, *Salmonella enterica* and *Shigella flexneri* [1]. *E*. *amylovora* is the causal agent of Fire Blight, a severe necrotic disease affecting species of the Rosaceae family, and of particular importance to the apple and pear fruit industries [2]. The infection strategy of *E*. *amylovora* hinges on the expression of the hrp (hypersensitive response and pathogenicity) type III secretion system (T3SS) and the production of an exopolysaccharide (EPS) matrix [3–5]. The matrix is mainly composed of the two EPSs, levan and amylovoran [4]. While levansucrase is the only enzyme designated to produce levan [6–9], amylovoran biosynthesis requires twelve proteins encoded by the genes of the *ams* operon and proteins involved in the galactose metabolism [10–12]. Amylovoran is one of the most important *E*. *amylovora* pathogenicity factors and strains with defective amylovoran biosynthesis are non-pathogenic [13]. Regulation of amylovoran production involves a complex signalling network, which is still poorly understood [14].

The amylovoran repressor gene *amyR* of *E*. *amylovora* is a member of the *ybjN* gene family, which is highly conserved among Enterobacteria [15,16], although distantly related members of the *ybjN* family appear in a wide spectrum of bacteria. The *ybjN* gene from *E*. *coli* has been defined as a stress-related gene, whose expression depends on the temperature and the growth stage [15]. Microarray analysis demonstrated that the over-expression of *ybjN* in *E*. *coli* induces several pleiotropic effects, promoting the transcription of stress-related genes such as the toxin-antitoxin module, SOS stress response, cold-shock, and phage-shock genes [17]. At the same time, *ybjN* down-regulates metabolic pathways such as energy metabolism and biosynthetic processes. *E*. *coli* strains over-expressing *ybjN* were observed to slow down their growth rate and decrease their swarming motility [17]. Moreover, there is a marked reduction in the biosynthesis of the capsule, biofilm formation and increased acid susceptibility. Expression of *ybjN* is induced under several stress conditions [18–20]. Therefore, *ybjN* in *E*. *coli* has an important role in stress-response and is able to regulate some virulence factors shared among other Enterobacteriaceae [17,21]. Recent studies have demonstrated that *E*. *amylovora* AmyR exerts a similar function on the metabolism of this organism in stress conditions [16]. Importantly, the over-expression of *amyR* negatively regulates the production of the two *E*. *amylovora* exopolysaccharides, amylovoran and levan. In fact, an *E*. *amylovora amyR* knockout strain demonstrated an overproduction of amylovoran 8-fold higher than the control in MBMA medium and colonies on LB plates exhibited a mucoid phenotype [16]. Therefore, *amyR* in *E*. *amylovora*, as with *ybjN* in *E*. *coli*, is involved in regulating exopolysaccharide production.

AmyR regulates the *ams* operon and *E*. *amylovora* strains with mutated *amyR* exhibited expression of *amsG*, *amsC* and *amsD* up to five-fold higher than wild-type levels [16]. Ams proteins are required for amylovoran production and their overexpression correlates with an increased EPS biosynthesis. Furthermore, the *amyR* mutation also significantly increased the transcription level of the genes *galF* and *galE* involved in galactose metabolism [16]. The encoded protein GalF could be considered an isoform of GalU, an uridylyltransferase responsible for UDP-glucose production that the second protein, GalE, converts to UDP-galactose [22–24]. These two enzymes together have a pivotal role in amylovoran production as the providers of galactose, the essential building block for this biosynthetic pathway [11]. In contrast, the over-expression of *amyR* led to a marked inhibition of amylovoran production, including in several amylovoran-overproducing mutants, and resulted in a loss of pathogenicity on apple shoots and leaves [16].

The Rcs phosphorelay system is an atypical two-component signal transduction (TCST) system present only in Enterobacteriaceae and positively regulates amylovoran biosynthesis by activating the *ams* operon transcription [25–27]. AmyR may regulate the Rcs phosphorelay system, potentially through interactions with RcsA or RcsB, the two response regulator proteins of the Rcs TCST, by preventing RcsB phosphorylation or interfering with RcsA DNA binding, or acting on the upstream transducers RcsC and RcsD [17,26,27]. However, while *amyR* and *rcsC* mutants show very similar changes in gene expression, *in vivo* infection assays show that *rscC* mutants are non-pathogenic while *amyR* mutants have similar virulence to wild-type strains, indicating that the activity of *amyR* is more complex than simply inhibiting the Rcs TCST system [16,26].

Recently the protein structure of a distantly related ybjN protein, DR1245 from *Deinococcus radiodurans*, was published [28]. The crystal structure of DR1245 revealed a fold similar to that of T3SS chaperones (T3C), despite not being implicated in any T3SS role. A structural similarity search also identified another distantly related YbjN protein from the cyanobacteria *Synechococcus elongatus* (unpublished, PDB ID: 2PLG). DR1245 does not have significant sequence similarity to the YbjN proteins of Enterobacteriaceae, with only 10% protein sequence identity to AmyR. DR1245 is an interaction partner of DdrB, a novel single stranded DNA binding protein upregulated in response to radiation stress [28]. The *E*. *coli ybjN* gene has since been shown to improve radiation resistance [29], indicating a shared stress response role of the *ybjN* family throughout Eubacteria.

This study reports the crystal structure of *E*. *amylovora* AmyR, the first representative structure of a YbjN protein from an Enterobacteriaceae species, which is the most highly divergent and intensely studied YbjN sub-family. The results provide specific implications for the mechanism of AmyR function, as well as relevance to the evolution and conserved functions of YbjN proteins across Eubacteria.

## Materials and methods

### Cloning

The *amyR* gene (Genbank FN434113.1, 1374785–1375270) was PCR amplified from genomic DNA of *E*. *amylovora* strain CFBP1430, using the forward primer 5’- ATTATTCCATGGGTATGGTTTCACTGGTCG—3’ and the reverse primer 5’- ATTATTCTCGAGTTAATGCAGCAGGGAATGGCTG -3’. Primers were designed to introduce an NcoI restriction site at the 5’ end and an XhoI restriction site at the 3’ end of the gene sequence. The PCR product was purified using the QIAquick PCR Purification Kit (Qiagen, Germany). The PCR product and the plasmid pETM-11 were double digested with the restriction enzymes NcoI and XhoI (NEB, USA). The digested PCR product and plasmid were run on a 1% agarose gel, and the appropriate bands were excised and gel purified (Qiagen, Germany). The *amyR* sequence was ligated into the pETM-11 expression vector [30], which encodes for a six histidine tag sequence and the Tobacco Etch Virus protease (TEV) cleavage site before the *amyR* gene insertion. The resultant protein after TEV protease cleavage has two non-native amino acids (GA) at the N-terminus. The construct was propagated in *E*. *coli* NovaBlue cells (EMD4 Biosciences, Germany) and was purified using a DNA miniprep kit (Sigma, USA). The plasmid molecular weight was checked by agarose gel electrophoresis after single and double digestion and the correctness of the inserted region was verified by sequencing at Eurofins facilities (Eurofins Genomics, Italy). *E*. *coli* BL21 (DE3) chemically competent cells (EMD4 Biosciences, Germany) were transformed with the pETM-11-*amyR* construct to express the recombinant protein.

### Expression and purification of the native protein

An overnight pre-culture was grown in 20 ml 2xYT medium containing kanamycin (30 mg L^-1^) at 310 K with shaking at 220 rpm. Two 2.5 L baffled flasks containing 1 L of LB medium were inoculated with 5 ml of the pre-culture and grown at 310 K with shaking at 220 rpm until they reached an OD600 of 0.6. The cultures were then cooled in an ice water bath for 10 min. Expression was then induced with 0.2 mM IPTG, and the cultures were incubated overnight at 293 K with shaking at 220 rpm. After 16 hours the cells were pelleted by centrifugation at 4500*g* for 10 min at 277 K, the supernatant removed, and the cells were re-suspended in ice-cold buffer A (50 mM HEPES pH 7.5, 500 mM NaCl, 20 mM imidazole, 2 mM DTT, 3% (w/v) glycerol) at 4 ml per gram wet weight of cell pellet with 0.2 mg ml^-1^ lysozyme (Sigma Aldrich, USA), 20μg ml^-1^ DNAse (Sigma-Aldrich, USA) and a protease inhibitor cocktail (Sigma-Aldrich, USA). The cells were disrupted by sonication on ice for 90 s using 30 s cycles (15.6 MHz) at 2 min intervals. The lysate was cleared by centrifugation at 25,000*g* for 30 min at 277 K and the supernatant filtered with a 0.45 μm cellulose acetate filter. The lysate solution was loaded onto a 5 ml HisTrap HP column (GE Healthcare, Sweden) pre-equilibrated with buffer A at a flow rate of 3 ml min^-1^. The column was washed with 20 ml of buffer A and the bound protein was eluted with a gradient of buffer B (50 mM HEPES pH 7.5, 500 mM NaCl, 500 mM imidazole) over 90 ml. The eluted AmyR peak was pooled in cellulose dialysis tubing (12.5 kDa MWCO) with 6His-tagged TEV protease at a 1:20 (TEV:AmyR) ratio, and dialysed overnight into buffer C (20 mM HEPES pH 7.5, 200 mM NaCl, 20 mM imidazole, 1 mM DTT) at 277 K to cleave the N-terminal 6His tag and remove the excess imidazole. The protein solution was then passed through a 5 ml HisTrap HP column pre-equilibrated with buffer C at 3 ml min^-1^ to remove the TEV protease, uncleaved AmyR, cleaved 6His tag, and other contaminating proteins. The flow through was collected and concentrated using a Vivaspin 20 ultrafiltration unit (Sartorius, Germany) with a 10,000 Da MWCO membrane to a volume of about 2 ml. The sample was run through a Superdex S75 16/60 column (GE Healthcare, Sweden) equilibrated with buffer D (20 mM HEPES pH 7.5, 200 mM NaCl, 1 mM DTT) at a flow rate of 1 ml min^-1^, and the AmyR peak was collected. All purification steps were carried out at 277 K. Purity of recombinant AmyR was confirmed by SDS-PAGE electrophoresis.

### Expression and purification of the selenomethionine substituted protein

An overnight pre-culture was grown in 20 ml of minimal media (1x M9 salts, 2 mM MgSO_4_, 0.4% (w/v) glucose, 0.1 mM CaCl_2_, 1x Kao & Michayluk vitamins, 1x trace metals (0.1 mM MnCl_2_-4H_2_O, 0.1 mM FeCl_3_, 5 μM ZnSO_4_-7H_2_O, 5 μM CoCl_2_-6H_2_O, 5 μM CuCl_2_-2H_2_O, 5 μM NiCl_2_-6H_2_O, 5 μM H_3_BO_3_, 5 μM NaMoO_4_-2H_2_O)) with 30 mg L^-1^ kanamycin at 310 K with shaking at 220 rpm. Two 2.5 L baffled flasks containing 1 L of minimal medium with 30 mg L^-1^ kanamycin were inoculated with 5 ml of the pre-culture and grown at 310 K with shaking at 220 rpm until reaching an OD600 of 0.5. The cultures were then supplemented with 100 mg of L-lysine, 100 mg of L-phenylalanine, 100 mg of L-threonine, 50 mg of L-isoleucine, 50 mg of L-leucine, 50 mg of L-valine, and 50 mg of L-selenomethionine to inhibit methionine synthesis and incorporate the selenomethionine [31]. The cultures were incubated at 293 K with shaking at 220 rpm for 20 minutes, then protein expression was induced with 0.2 mM IPTG and the cultures were grown for a further 16 h.

The cells were harvested and the protein extracted and purified following the method described for the native protein, with the addition of 10 mM DTT to all buffers. Selenomethionine incorporation was confirmed by ESI mass spectrometry.

### Crystallisation

The purified native AmyR was concentrated to 10 mg ml^-1^ in buffer D. Protein concentration was determined by direct UV measurement at 280 nm on a NanoVue spectrophotometer (GE Healthcare, Sweden) using an extinction coefficient of 12,490 M^-1^ cm^-1^, calculated by ProtParam (ExPASy) [32]. Crystallisation trials were performed using the microbatch-under-oil method in 96-wells MRC plates (Molecular Dimensions, UK) using volatile oil (MD2-06, Molecular Dimensions, UK) at 293 K and 277 K. Drops of 1 μl of protein solution were equilibrated against the same volume of commercially available crystallization kits PACT, CSS1, CSS2, JCSG+, MIDAS, and MORPHEUS (Molecular Dimensions, UK). Crystals grew in the condition JCSG+ B10, which consists of 0.2 M magnesium chloride, 0.1 M sodium cacodylate pH 6.5, 50% (v/v) PEG 200. This crystallisation condition was optimised by sitting-drop vapour-diffusion, using 0.5 μl of protein solution with 0.5 μl of precipitant and 100 μl reservoirs in MRC plates (Molecular Dimensions, UK), with varying pH, temperature, and protein, salt and PEG concentrations. Optimum crystals grew at 293 K 4–8 days with a protein concentration of 8 mg ml^-1^ using 0.1 M sodium cacodylate pH 6.9, 0.2 M magnesium chloride, 46% (v/v) PEG 200 at 293 K. Crystals were harvested from the plate using cryoloops (Hampton Research, USA) and flash cooled in liquid nitrogen with cryoprotection provided by the crystallisation condition.

Crystallisation of the selenomethionine substituted protein was attempted under the same conditions as the native protein, but only produced showers of small crystals. The conditions were optimised by the same method described for the native protein. Optimum crystals grew at 293 K with a protein concentration of 8 mg ml^-1^ and the precipitant condition 0.1 M sodium cacodylate-Tris pH 7.3, 0.3 M magnesium chloride, 42% (v/v) PEG 200 at 293 K. The sodium cacodylate-Tris buffer was prepared by titrating 1 M unbuffered Tris into 1 M sodium cacodylate pH 6.9 until reaching the desired pH of 7.3. This condition produced crystals of a similar quality to those observed for the native protein, and were collected and flash cooled in the same manner.

### Diffraction data collection and processing

X-ray diffraction data of the native protein were collected to a resolution of 1.95 Å on beamline I04-1 at Diamond Light Source (DLS), Didcot, UK. Data for MAD phasing were collected from the selenomethionine substituted protein to 2.12 Å resolution on beamline I04 at DLS. Data were processed using XDS [33] within XIA2 [34]. Data collection statistics are provided in Table 1. The Se substructure was determined using SHELX [35]. Programs of the CCP4 suite [36] were used for structure solution and model refinement. Building of the structure was aided by automated procedures in Buccaneer [37] and manual building was performed with COOT [38]. The selenomethionine substituted protein was used as a search model for molecular replacement with the native data using PHASER [39]. The structure was refined with REFMAC5 [40], and validated with MolProbity [41]. Baverage was used to extract mean B factors values [36]. Figures of the structures were prepared with CCP4MG [42]. Refinement statistics are provided in Table 2.

**Table 1:** Data collection statistics for native and selenomethionine substituted AmyR. Values in parentheses are for the highest resolution shell.

	Native	Selenomethonine		
		Peak	Inflection Point	High Energy Remote
Diffraction source	DLS I04-1	DLS I04	DLS I04	DLS I04
Wavelength (Å)	0.91730	0.9794	0.9796	0.9740
Temperature (K)	100	100	100	100
Detector	Dectris Pilatus 6M	Dectris Pilatus 6M	Dectris Pilatus 6M	Dectris Pilatus 6M
Space group	*P* 2_1_2_1_2	*P* 2_1_2_1_2	*P* 2_1_2_1_2	*P* 2_1_2_1_2
*a*, *b*, *c* (Å)	137.16, 49.82, 57.79	138.03, 49.62, 57.81	138.03, 49.62, 57.81	138.03, 49.62, 57.81
α, β, γ (°)	90, 90, 90	90, 90, 90	90, 90, 90	90, 90, 90
Resolution limit (Å)	53.26–1.95 (2.14–1.95)	53.34–2.13 (2.19–2.13)	53.34–2.12 (2.18–2.12)	49.66–2.22 (2.28–2.22)
Completeness	99.7 (99.6)	97.8 (79.0)	97.4 (76.2)	99.8 (98.5)
Multiplicity	5.0 (5.0)	24.7 (17.8)	24.6 (17.5)	25.4 (20.0)
I/sigma	9.2 (1.8)	23.2 (1.4)	24.0 (1.4)	17.7 (1.3)
Rmeas(I)	0.080 (1.230)	0.124 (2.282)	0.110 (2.238)	0.136 (2.474)
Rmeas(I+/-)		0.091 (2.242)	0.089 (2.203)	0.125 (2.411)
CC half		0.999 (0.512)	1.000 (0.582)	0.999 (0.512)
Wilson B factor	37.15	47.36	46.34	48.48
Anomalous completeness		97.6 (78.3)	97.2 (75.4)	99.8 (98.1)
Anomalous multiplicity		13.0 (9.0)	12.9 (8.9)	13.4 (10.3)
Anomalous correlation		0.957 (0.032)	0.927 (0.024)	0.802 (0.030)
Total observations	147243 (34524)	553915 (23088)	557484 (22227)	516612 (28673)
Total unique	29587 (6947)	22462 (1298)	22681 (1272)	20357 (1434)

**Table 2:** Refinement statistics for native and selenomethionine substituted AmyR.

	Native	Selenomethionine
R_work_	0.224	0.215
R_free_	0.269	0.274
RMS deviation from ideal values		
Bond lengths (Å)	0.016	0.016
Bond angles (°)	1.797	1.833
Average B-factor (Å^2^)		
Protein atoms	42.82	50.88
Water molecules	43.49	49.71
Ramachandran plot		
Favoured (%)	284 (99.0)	283 (98.6)
Allowed (%)	1 (0.3)	4 (1.4)
Outliers (%)	2 (0.7)	0 (0)

### Bioinformatics

Similar protein structures to AmyR were identified using the protein structure comparison service PDBeFold from the European Bioinformatics Institute (http://www.ebi.ac.uk/msd-srv/ssm) [43] and proteins structure alignments performed in COOT [38]. Similar protein sequences to AmyR were identified by NCBI protein BLAST (http://blast.ncbi.nlm.nih.gov/Blast.cgi) [44] and the NCBI Conserved Domain Database (CDD) [45]. The CDD was used to generate a list of 100 aligned sequences from the protein family “YbjN putative bacterial sensory transductor regulator” using the type selection “the most diverse members”, and the sequences were exported in mFasta format. Sequences of larger proteins containing YbjN domains were removed, and the sequences of *E*. *amylovora* AmyR, *E*. *coli* YbjN, *D*. *radiodurans* DR1245 and *S*. *elongates* T110839 were added for comparison. The protein sequences were aligned and visualised using M-Coffee from the Centre for Genomic Regulation of Barcelona (http://tcoffee.crg.cat/apps/tcoffee/index.html) [46,47] and ESPript 3.0 (http://espript.ibcp.fr) [48]. A phylogenetic tree was built using Clustal Omega [49].

## Results and discussion

### Crystallisation and structure solution

Two different crystal forms developed in the conditions used for crystallisation; rectangular crystals and rhombic plates (S1 Fig), with the thicker rectangular crystals regularly producing better diffraction quality crystals. No structures of similar sequence were identified in the protein data bank, and all attempts at solving the native protein structure by molecular replacement failed to produce a solution, including using a type III secretion chaperone model, CesT (PDB: 1K3E), identified by homology modelling in ROBETTA [50].

The crystal structure of AmyR substituted with selenomethionine was solved at 2.12 Å resolution by multiple wavelength anomalous diffraction (PDB: 5FRK), and native AmyR was determined at 1.95 Å by molecular replacement (PDB: 5FR7) using the selenomethionine structure as a search model. Both crystals were orthorhombic and belonged to space group *P* 2_1_2_1_2.

### Overall structure of AmyR

**Fig 1:**
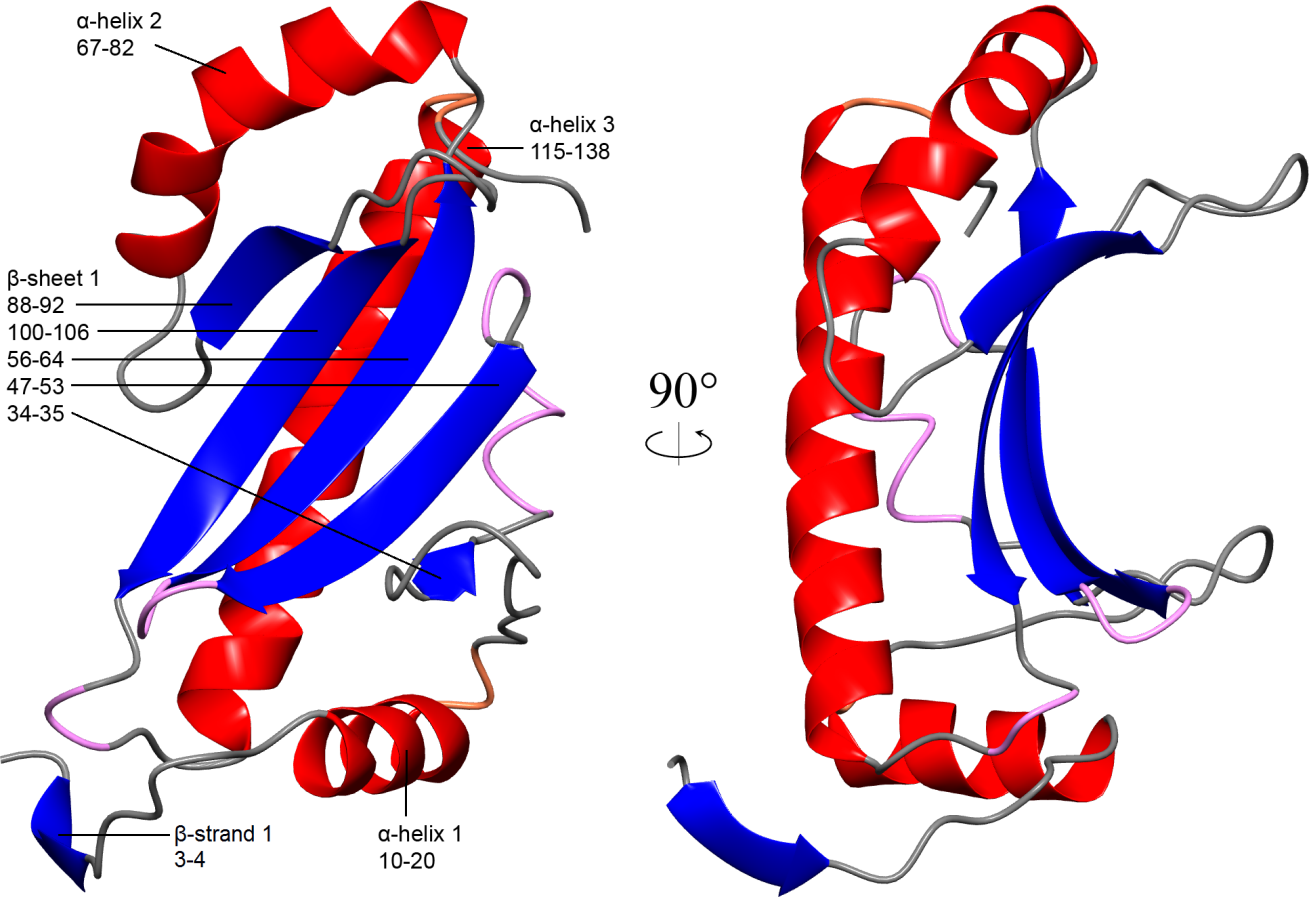
Ribbon representation of the overall structure of the AmyR monomer. The AmyR monomer is coloured by secondary structure. Secondary structure elements are labelled with residue numbers. The right panel is rotated 90°. β-strand 1 interacts with the equivalent residues in the dimer, shown in Fig 2.

**Fig 2:**
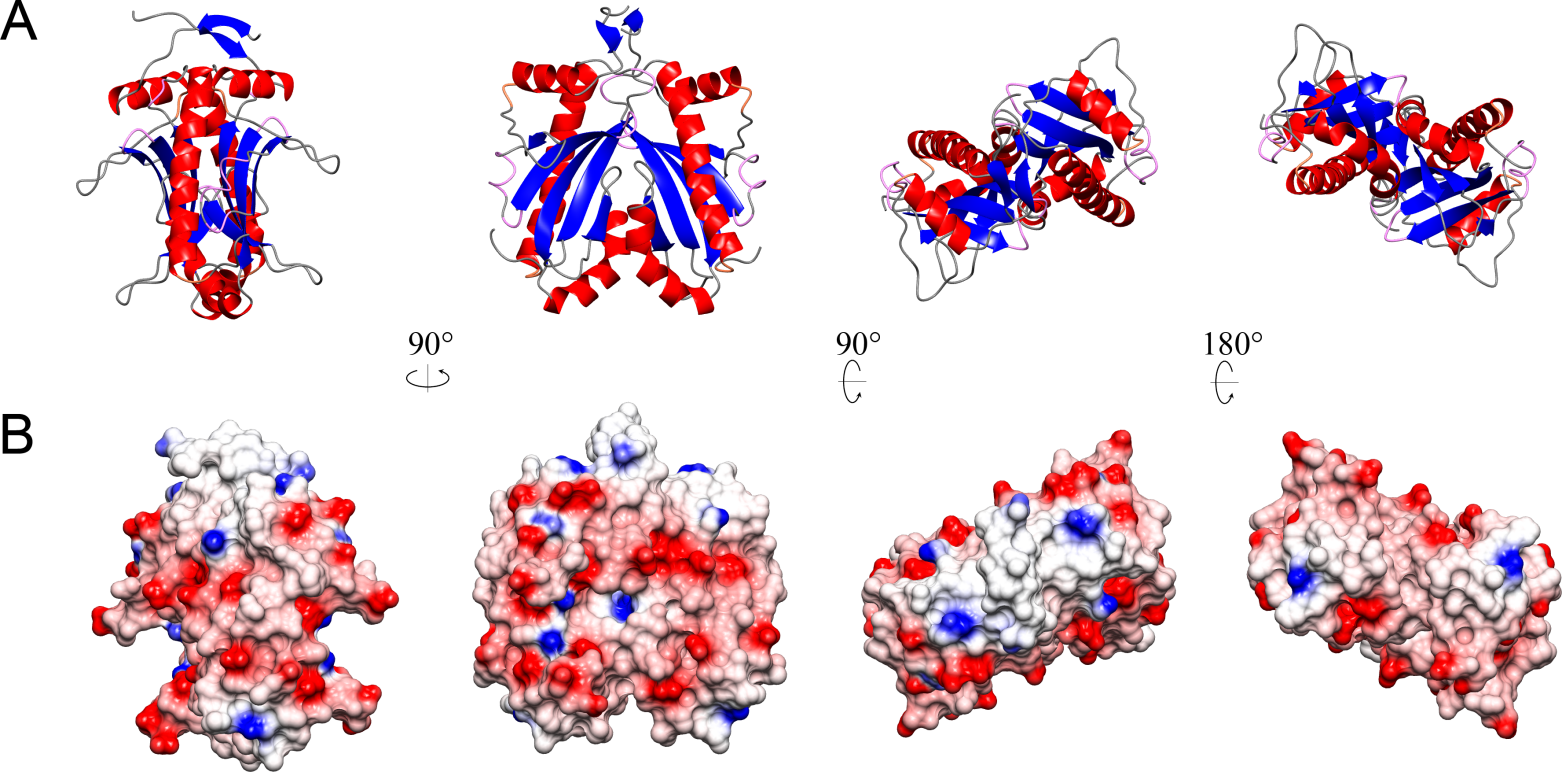
Rotated views of the *E*. *amylovora* AmyR dimer. (A) Ribbon diagram, coloured by secondary structure. (B) Surface topography, coloured by surface charge at pH 7.4, red = negative, blue = positive.

**Fig 3:**
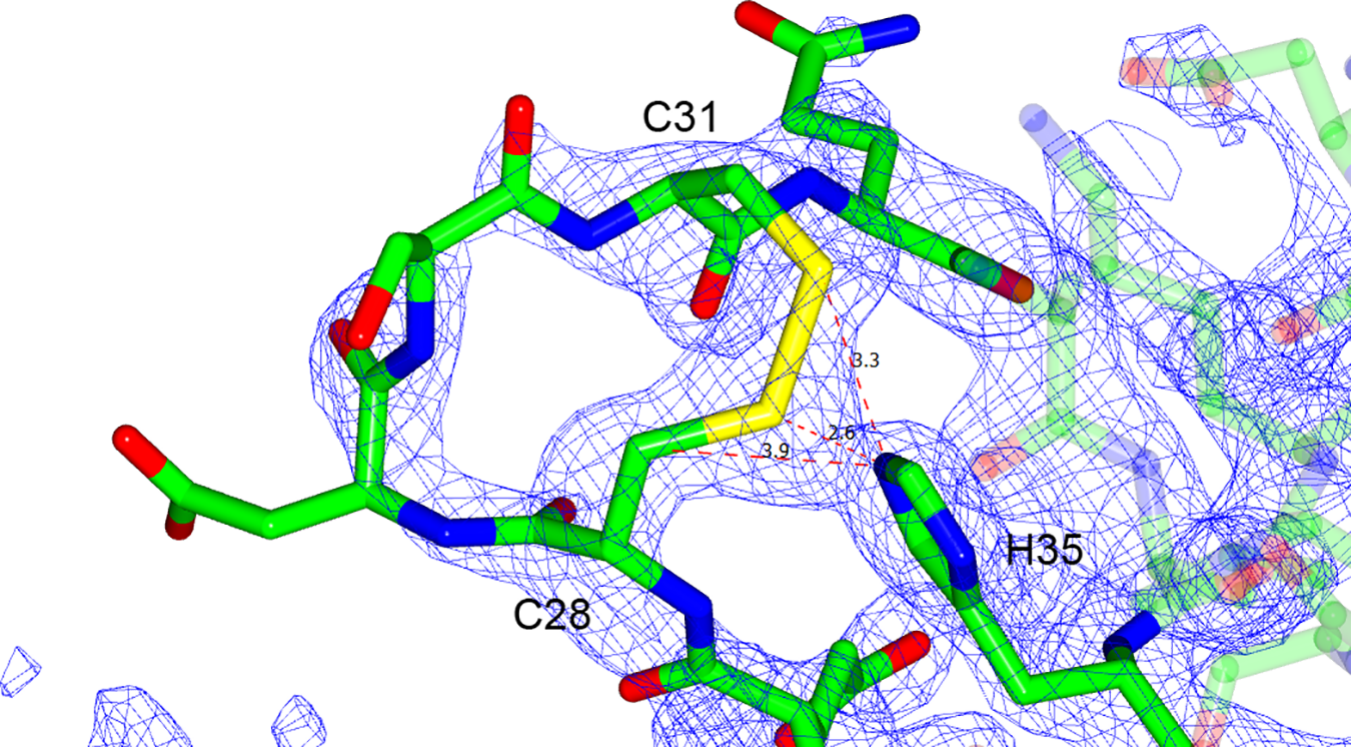
Coordination of residues in the β-sheet flanking loop. The loop of residues 27 to 33, showing the disulphide bond between C28 and C31 coordinated with H35. The electron density shows the coordinated region of the loop is well ordered, while the tip remains flexible. Atomic distances between H35NE2 and the disulphide bridge are indicated in angstroms.

**Fig 4:**
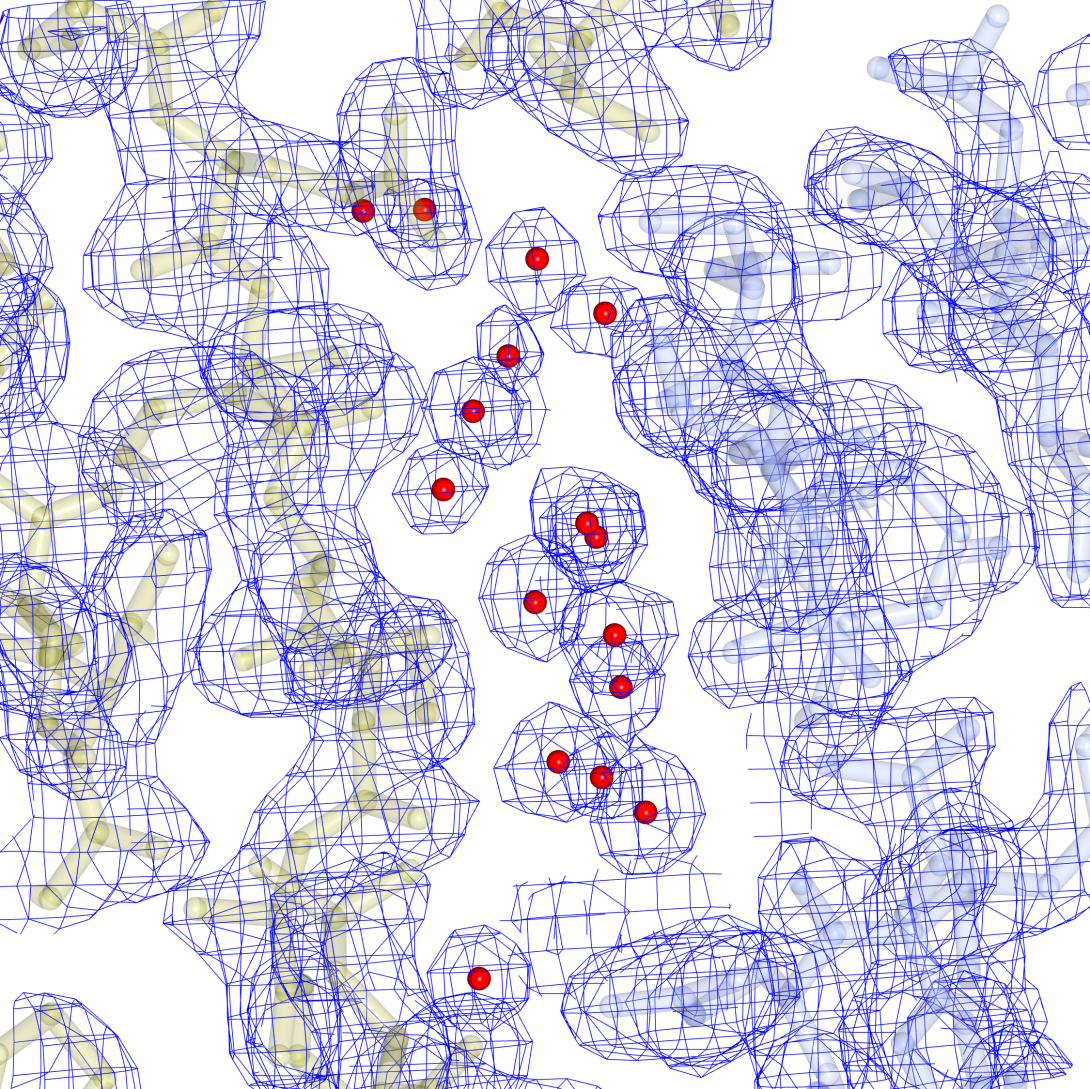
The water molecules between the dimer interface. Cross-section of the hydrophilic pocket at the dimer interface, showing the A and B chains coloured in gold and silver, with the highly ordered water molecules contained within.

AmyR forms a homodimer in the asymmetric unit with a solvent content of 60.12% (Vm = 3.08 Å^3^/Da). Residues 1 to 144 of chain A and 1 to 145 of chain B were modelled and proved an excellent fit to the density. The remaining C-terminal regions were mostly unstructured, lacked defined density, and could not be modelled. The protein contains 3 α-helices (residues 10–20; 67–82; 115–138) surrounding a 5 stranded antiparallel β-sheet (residues 34–35; 47–53; 56–64; 88–92; 100–106) with the concave surface facing the solvent and flanked by two protruding loops (residues 27–33; 93–99) to create a channel (Figs 1 & 2). Flanking loop_27-33_ is stabilised by a disulphide bridge between C28 and C31 which is coordinated by the side-chain of H35 from the β-strand (Fig 3). This stabilises the loop, which otherwise lacks coordinating interactions, resulting in a relatively rigid structure excluding residue S30 at the tip of the loop. The density for the disulphide bridge does not fit perfectly and it is likely some cysteines in the crystal were reduced, but the density favours the majority forming the bridge. Flanking loop_93-99_ on the other side of the β-sheet forms a crystal contact at its tip between D95 and T68 of the adjacent molecule, resulting in it being highly coordinated in the described structure. However, loop_93-99_ otherwise lacks coordinating interactions, so in solution could be quite flexible and capable of folding over to partially encase the channel formed by the β-sheet and flanking loops. Half of the dimer interface is characterized by hydrophobic interactions between the bent α-helix_67-82_ and β-strand_88-92_ of each chain, while the other half of the interaction face forms a hydrophilic pocket containing 18 highly ordered water molecules (Fig 4), and is capped by hydrogen bonding between the two N-terminal tails.

**Fig 5:**
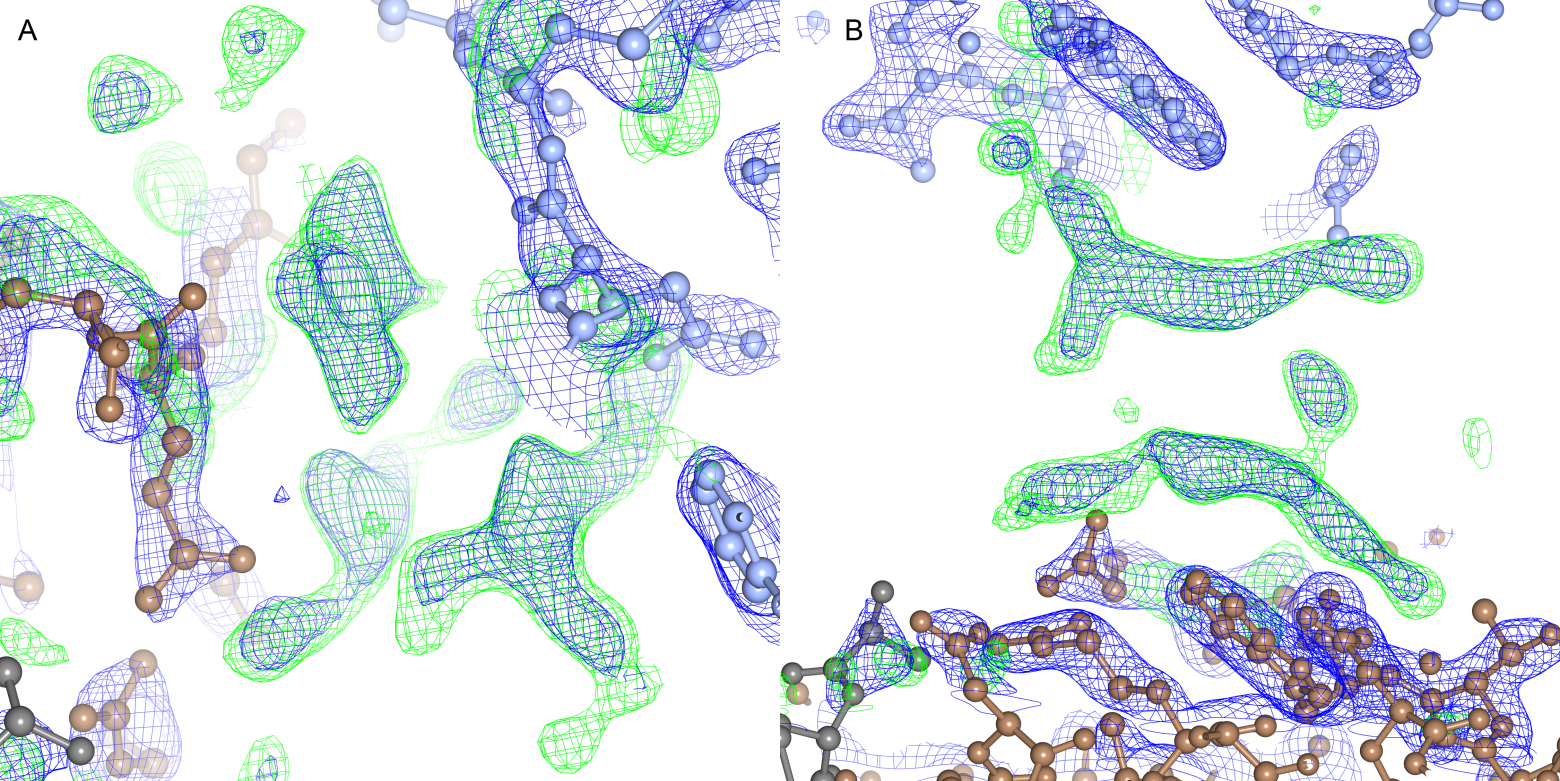
Unmodelled density at crystal contact between molecules. (A) Electron density and difference map of the unmodelled density at the crystal contact. The main crystal contact forming density is central, while the two other chains are seen below. (B) An alternative view of the two chains.

The carboxy-terminal 17 amino acids of AmyR form an unstructured chain, accounting for 10% of the protein sequence. The electron density is observed protruding into the solvent channel from both A and B chains of the dimer, and can be followed with decreasing accuracy for roughly 4 residues before the density becomes completely ambiguous. The chain appears to reconnect with the main structure to form a crystal contact between residues 26 to 28 of chain A with the equivalent residues of chain B on the symmetry related molecule, but density could not be unambiguously modelled because of the short length of the observed density without any clearly defined structural features (Fig 5).

### YbjN proteins share a fold with T3SS chaperone proteins but appear to function independently of the T3SS

**Fig 6:**
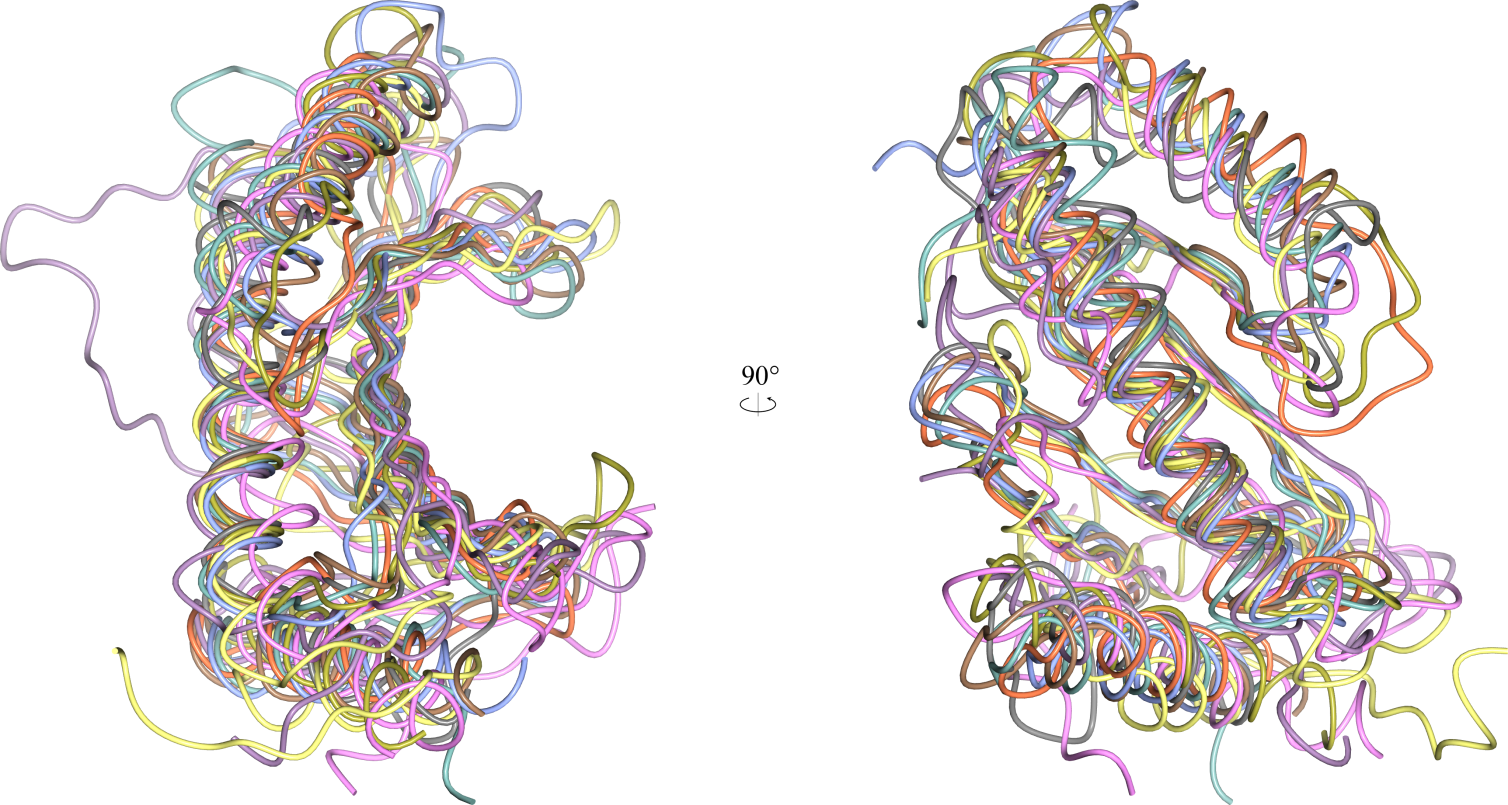
Structural alignment of AmyR monomer with other YbjN and T3C proteins. The two views are rotated horizontally 90°. The alignment includes the PDB entries 1JYO:D (*Salmonella enterica* SicP, ice blue), 1S28:A (*Pseudomonas savastanoi* AvrPphF ORF1, gold), 1TTW:A (*Yersinia pestis* SycH, coral), 1XKP:B (*Yersinia pestis* SycN, grey), 2PLG:B (*Synechococcus elongates* T110839, pink), 3EPU:A (*Salmonella enterica* STM2138, sea green), 3KXY:J (*Escherichia coli* ExsC, brown), and 4H5B:A (*Deinococcus radiodurans* DR_1245, lilac), and 5FR7:A (*Erwinia amylovora* AmyR, yellow).

A structural homology search with PDBeFold [43] showed the closest similar structures to AmyR to be *D*. *radiodurans* DR1245, *S*. *elongatus* T110839, and class I T3C proteins. Structural alignment shows how highly conserved the domain architecture is (Fig 6). There is some variation in the length and orientation of the periphery loops and secondary structural elements, but the core domain structures are almost identical. The main variation is the orientation of the dimer interaction, rather than the protein fold itself.

Despite the structural similarity with the class I T3C proteins, the genomic location of *amyR* does not conform with a class I T3C. The genes for class IA T3Cs are located adjacent to the genes of the type III effector which they partner with [51]. In contrast, the next downstream gene from *amyR* in the *E*. *amylovora* genome is *potF* (genbank CBA20255.1) [52,53]. This gene is annotated to code a periplasmic receptor component of the PotFGHI putrescine ABC transporter, with high similarity to other such proteins in Enterobacteriaceae species, including the *E*. *coli* homologue with 78% protein sequence identity, which has been structurally and functionally characterised [54]. Upstream of *amyR* is the *mdaA* gene, coding for an oxygen-insensitive nitroreductase [52,53]. Neither PotF or MdaA are T3SS associated proteins. PotF is targeted to the periplasm, which is bypassed by the T3SS, while MdaA is cytoplasmic. Therefore, AmyR cannot act as a class IA T3C for PotF or MdaA. Class IB T3Cs are located in T3SS related operons [55], which again does not apply to *amyR*.

To confirm this is a common feature of *ybjN* genes, the genomic locations of *ybjN* genes from 13 additional species were investigated. Species were selected on the criteria of containing both a *ybjN* gene and a T3SS, and that the whole genome sequence was available, including virulence plasmid sequences where applicable. Assuming that the genomic organisation of closely related species would be similar, the selection was limited to one member of any given Genus. The species *Escherichia coli*, *Yersinia pestis*, *Salmonella enterica*, *Shigella flexneri*, *Vibrio parahaemolyticus*, *Serratia plymuthica*, *Burkholderia glumae*, *Chlamydia trachomatis*, *Sodalis praecaptivus*, *Ralstonia insidiosa*, *Rhizobium phaseoli*, *Photorhabdus asymbiotica*, and *Desulfovibrio vulgaris* were included. In no case were any *ybjN* genes located in T3SS gene clusters, or adjacent to T3SS associated proteins (as some effector proteins are not located in T3SS operons, e.g. *E*. *amylovora* AvrRpt2). These results show that *ybjN* genes occupy distinct genomic locations from T3Cs.

The phenotypes observed by varying AmyR expression cannot be explained purely by the function of a T3C. Secretion chaperones facilitate secretion and/or stabilise cytoplasmic T3SS effector and gate proteins. The chaperoning effect should be the same when expressed sufficiently or to excess. While an *amyR* knockout mutation would be expected to have an observable effect, the overexpression of AmyR results in regulatory changes in *E*. *amylovora*, which cannot be explained by AmyR functioning solely as a T3C [16]. None of the proteins secreted via the T3SS have been shown to regulate *E*. *amylovora* exopolysaccharide levels, and T3SS mutants can still produce wild-type levels of exopolysaccharide [56]. Furthermore, *amyR* knockouts show increased virulence, rather than decreased, as would be expected for a T3C [16].

Some class I T3Cs have shown transcriptional regulation activity along with chaperoning of T3SS proteins, such as *Shigella flixneri* Spa15 [57], *Pseudomonas aeruginosa* ExsC [58], and *Pseudomonas syringae* HrpG [59]. Unlike AmyR and other YbjN proteins, the currently identified class I T3Cs with transcriptional regulation activity are located in T3SS operons, their interactions are with T3SS associated proteins, and their regulatory activity is restricted to T3SS gene clusters [57–60]. One notable exception is *Chlamydia trachomatis* Scc4 (CT663), which can trigger global changes in gene expression by directly interacting with RNA polymerase to inhibit σ^66^-dependent transcription [61,62]. However, Scc4 is classified in the CesT superfamily of T3Cs [45], genetically distinct from YbjN proteins, and is located in a cluster of T3SS associated genes in the bacterial genome [63].

The functions of related proteins also support YbjN proteins not performing a T3SS role, as YbjN proteins have been generally characterised as stress-response sensory transduction regulators. The reported functional analysis of the *E*. *coli* homolog YbjN has no relation to the T3SS [15,17]. The structural homolog DR1245 from *D*. *radiodurans* also performs functions and interactions entirely unrelated to infection or the T3SS [28]. Despite the clear structural homology, none of these proteins show sequence homology to T3SS chaperones, while *E*. *amylovora* AmyR and *E*. *coli* YbjN have 67% sequence identity and are functionally conserved [16,17]. Furthermore, the CDD shows that YbjN family proteins are present throughout Eubacteria, including in species which are non-pathogenic and lack a T3SS [45]. Even though pairwise alignments of distantly related YbjN proteins might lack sequence similarity, the CDD shows through multiple alignment that YbjN proteins classify into a distinct protein family, which excludes T3Cs. While a T3SS related role cannot be completely ruled out for all YbjN proteins, at this time there is no evidence for YbjN proteins, including AmyR, to function in conjunction with the T3SS. Therefore, the shared fold between YbjN proteins and T3Cs suggests a shared mechanism and ancestral origin while performing different cellular functions.

### Identification of conserved residues and regions in YbjN proteins

**Fig 7:**
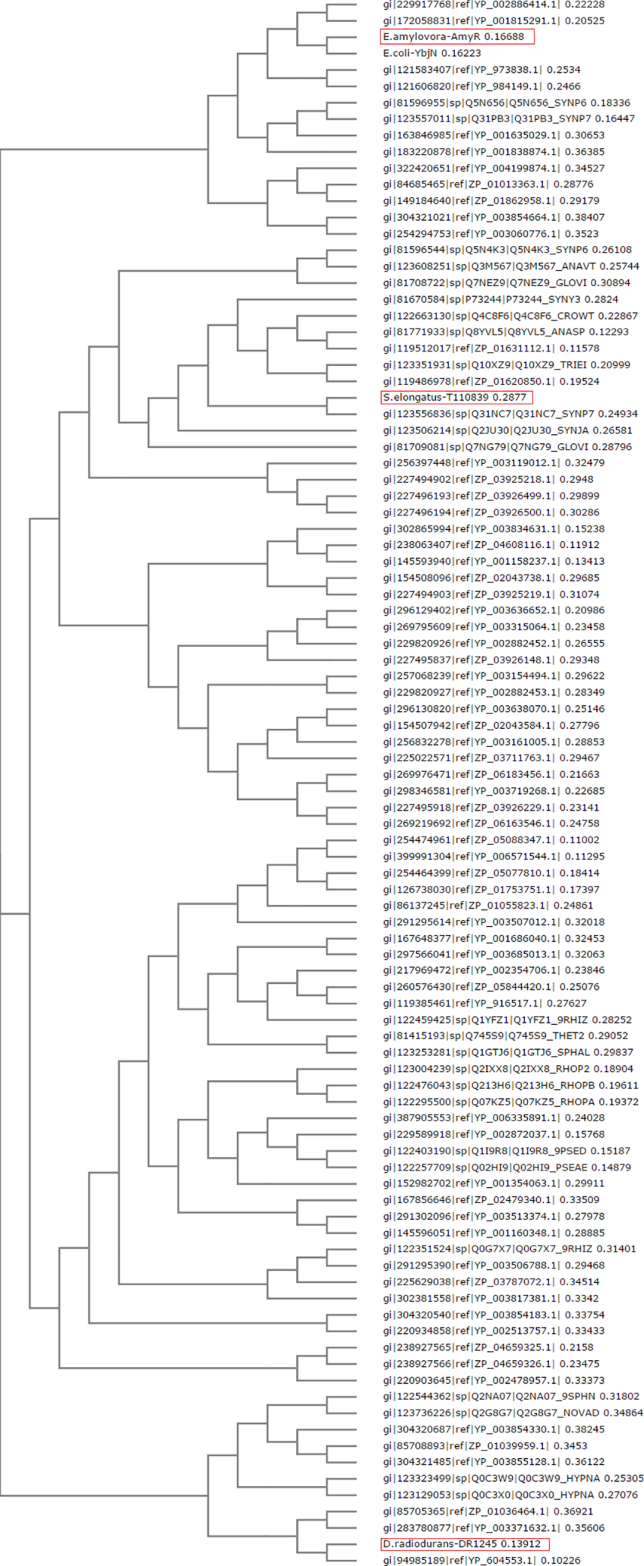
Phylogenetic tree of 100 YbjN protein sequences selected as described in methods. The three sequences with protein structures are boxed in red.

The sequences of 100 YbjN family proteins were aligned from a diverse selection of species to identify conserved regions or specific residues. The alignment showed consensus regions among species that were more closely evolutionarily related, but no overall consensus sequence for YbjN proteins. The Enterobacteriaceae species were the most divergent of the sequences, with *E*. *amylovora* and *E*. *coli* sharing the lowest identity score against the average of all sequences, while the majority of other sequences rated between 40 and 52. The sequences from Cyanobacteria made up the remainder of sequences with low identity to the consensus, and also lacking similarity to the Enterobacteriaceae sequences represent an independent variation from the YbjN consensus. A phylogenetic tree of the protein sequences (Fig 7) shows that the three presently determined YbjN protein structures (AmyR, DR1245, and T110839) are placed in each of the three major divisions of the tree. Therefore, these three structures represent three of the most divergent members of the YbjN protein family, with almost no sequence similarity between them, yet the alignment of their structures shows how extremely conserved the protein fold is within the YbjN family (Fig 6).

The M-Coffee alignment of the YbjN protein sequences (S2 Fig) revealed two regions which were the most highly conserved, which correlated to residues 13–24 and 75–88 of the AmyR sequence, while ESPript identified 21 highly conserved positions (S3 Fig) correlating to residues L14, L18, H38, L77, S78, I80, N81, T86, K88, F90, L91, L101, L103, L107, A109, L113, T114, F118, M122, I129, and A136. Residue N97 on the β-sheet flanking loop_93-99_ was identified but shown to be incorrectly assigned, because this loop is significantly longer in AmyR than for most YbjN proteins, and the correct alignment was with L101.

**Fig 8:**
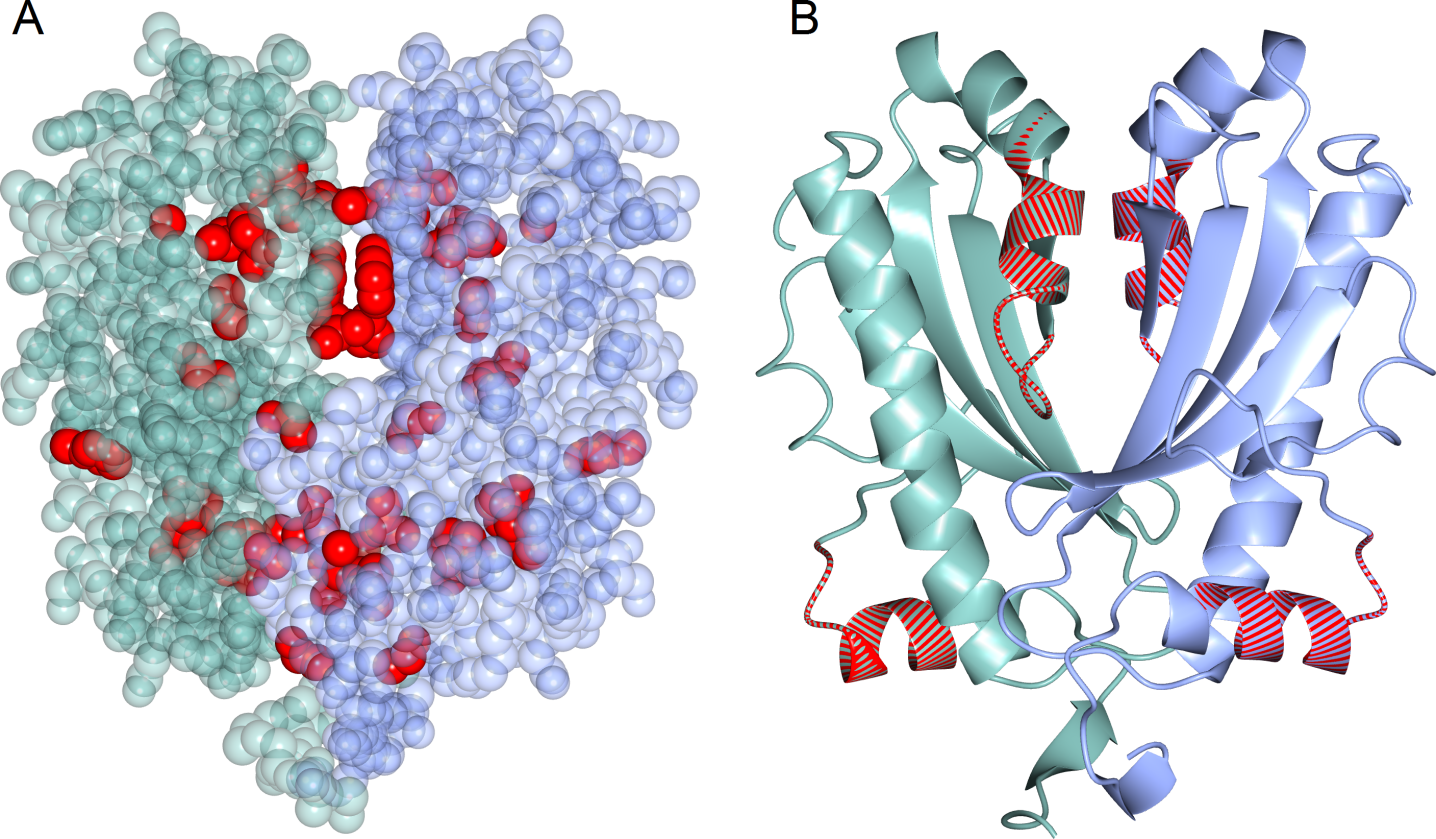
Location of conserved regions and residues of YbjN proteins superimposed on *E*. *amylovora* AmyR structure. (A) Sphere representation of AmyR displaying the two chains of the dimer in grey and green, with the atoms of conserved sidechains shown in red Van der Waals spheres. The main chains are partially transparent to show that the conserved residue side chains are mostly internalised or at the dimerization interface. (B) The same view as a ribbon diagram, with the YbjN protein conserved regions in red.

Almost all the conserved residues had internalised side chains mediating the hydrophobic interactions between secondary structure elements in the core of the protein and hydrophobic dimerization interface (Fig 8A). There will only be limited amino acids that could adopt these positions with side chains capable of fitting in the hydrophobic spaces between secondary structure elements without significantly altering their orientation, given the high structural conservation of the fold adopted by YbjN and T3C proteins. The few conserved hydrophilic residues also mediate structural interactions with minimal solvent exposure. The most highly conserved region was residues 75–88, which also contained around one quarter of the specific residues identified. This mapped to α-helix_67-82_ and the subsequent loop, which mediate the hydrophobic dimerization interface (Fig 8B). The other conserved region in the alignment, between residues 13–24, seemed to be poorly conserved in Enterobacteriaceae species, and the AmyR sequence was a poor match in this region. This region mapped to the C-terminal end of the first α-helix of the AmyR structure, which was not predicted to be involved in interactions. The two most highly conserved amino acids in this region were internally facing hydrophobic residues, with side chains facing the internal side of the β-sheet.

This analysis shows that there are no conserved surface areas in YbjN proteins across the entire protein family. This corresponds well with the idea that YbjN proteins, like T3Cs, share a common fold and common mechanism of action, but interact with different target proteins which are determined by the solvent exposed surface, particularly on and around the β-sheet. Therefore, closely related proteins like *E*. *amylovora* AmyR and *E*. *coli* YbjN are functionally conserved, while other distantly related YbjN proteins are active through different specific interactions.

**Fig 9:**
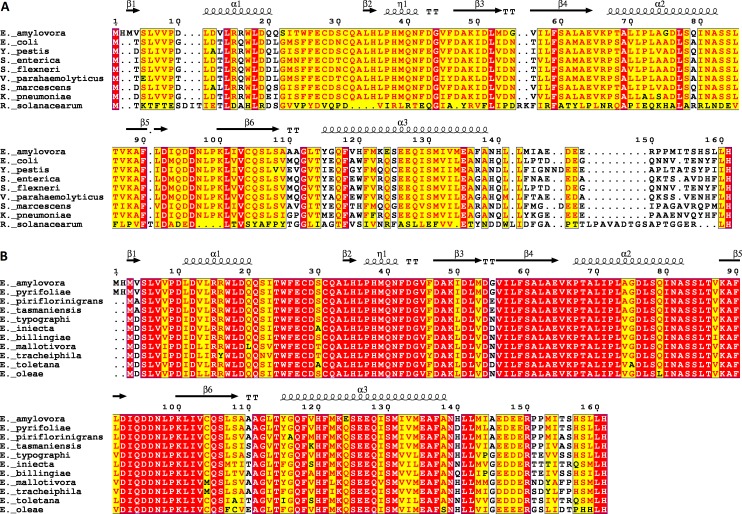
Sequence alignments of YbjN proteins. (A) Alignments of YbjN protein sequences from Enterobacteriaceae species, *Erwinia amylovora* (NCBI accession CBA20254); *Escherichia coli* (AJE55158); *Yersinia pestis* (ABP40742); *Salmonella enterica* (WP_000624814); *Shigella flexneri* (ABF03066); *Vibrio parahaemolyticus* (KKY40576)*; Serratia marcescens* (OKP20636); *Klebsiella pneumonia* (AHM80410); and Beta Proteobacteria *Ralstonia solanacearum* (WP_042591288). (B) Alignment of YbjN protein sequences from *Erwinia* species, *E*. *amylovora* (CBA20254); *E*. *pyrifoliae* (CAX56090); *E*. *piriflorinigrans* (WP_023654581); *E*. *tasmaniensis* (WP_012441893); *E*. *typographi* (WP_034891635); *E*. *iniecta* (WP_052896595); *E*. *billingiae* (WP_013201489); *E*. *mallotivora* (WP_034941268); *E*. *tracheiphila* (WP_016189698); *E*. *toletana* (WP_017802422); *E*. *oleae* (WP_034945603).

To better understand the important regions of Enterobacteriaceae specific YbjN proteins, an alignment was performed with protein sequences from 7 other Enterobacteriaceae species selected for their importance to infectious disease (*Escherichia coli*, *Yersinia pestis*, *Salmonella enterica*, *Shigella flexneri*, *Vibrio parahaemolyticus*, *Serratia marcescens*, *Klebsiella pneumoniae*), and one Beta Proteobacteria as an outlier (*Ralstonia solanacearum*) (Fig 9A). This highlighted the high sequence conservation within Enterobacteriaceae, with all sequences showing between 64% to 74% identity with *E*. *amylovora* AmyR. The main area of divergence was the C-terminal 24 amino acids, accounting for the tip of the last α-helix, proceeding loop, and unstructured tail in the AmyR structure. However, a conserved Leu-His motif was observed at the terminal two amino acids. The presence of disordered C-termini in similar structures indicates this is a conserved feature in YbjN proteins. There were also minor divergences in residues 10–21 and 110–125, corresponding to α-helix 1, and the preceding loop and start of α-helix 3, respectively.

Alignment of the YbjN protein sequences from 11 *Erwinia* species also only showed significant variance in the unstructured region of the C-terminus, with nearly identical folded domain sequences (Fig 9B). Since there is not significant variation in the protein interaction regions of YbjN proteins in Enterobacteriaceae, their interaction partners are also expected to be homologous. Differences in activity between Enterobacteriaceae species will be a result of variation in the downstream transcriptional regulation pathways, rather than the direct activity of the YbjN proteins. However, in more divergent bacteria this may not be the case.

### Protein interactions

Given their shared architecture, YbjN proteins will most likely interact with partner proteins in the same manner as Class I T3Cs. These function through protein-protein interactions by binding unfolded chains and secondary structure elements of partially or fully folded peptides in an ATP-independent manner [64,65]. Binding is strong and specific, and effector release is coupled to the activity of an independent ATPase at the T3SS gate [66].

The only reports of the observed fold in complex with interaction partners have been for T3Cs, co-crystallised with either effector proteins [67,68] or T3SS gate proteins [69]. The interaction is usually observed as the wrapping of unstructured or partially structured amino acid chains around the chaperone dimer perpendicular to the axis of the dimerization interface (PDB examples: 1TTW [67]; 1JYO [70], but interactions with folded domains are also possible (PDB examples: 2FM8 [71]; 1XKP [69]). Analysis of the surface charges of AmyR show many negatively charged groups surrounding the dimer in the plane of chain wrapping observed in the T3C structures (Fig 2B), but hydrophobic interactions are also likely to be important for a chaperone-like protein. Surface grooves are oriented to accommodate interactions with peptide chains in the direction similar to T3Cs, including the internal groove of the beta-sheet and flanking loops (Fig 2A), which is capable of fitting α-helical assemblies or unfolded chains.

Class I T3Cs are divided into two subclasses, class IA which associates with a single effector protein, and class IB which are able to associate with several effectors [55]. Both subclasses share the same fold and cannot be distinguished by comparing structural features. There was some indication that class IB chaperones adopt a wider dimerization conformation with a reduced interface [72]. However, there is significant variation at the dimer interface within class IA, class IB and YbjN proteins, from the increasing number of structures now available, and insufficient evidence to classify YbjN proteins as targeting single or multiple interaction partner proteins based on quaternary structure [65,73]. If AmyR has more than one interaction partner it could interact with the Rcs phosphorelay system, since matching changes in gene regulation are observed between *amyR* and *rcsC* mutants, but also interact with one or more other pathways, resulting in the observed phenotypic differences between *amyR* and *rcsC* mutants [16,26,27].

The functional significance of the disordered C-terminus is unknown. The sequence is not well conserved even in closely related species, yet the presence and relative length is conserved, along with the C-terminal LH motif (Fig 9). Its function may be similar to the P23 chaperone where specific binding interactions are determined by the folded domain, while the unstructured C-terminus only assists in non-specific interactions of proteome stabilization [74]. Alternatively, the conserved terminal LH residues could be a binding motif on a flexible arm, similar to the disordered C-terminus of the DnaK chaperone [75].

### Gene regulation by AmyR through specific interactions

The overexpression of AmyR under a non-native promoter showed that the changes in transcriptional levels of other genes are a direct result of the presence of the AmyR protein, and not just co-regulation of the AmyR promoter [16]. Transcriptional regulation may occur through an inhibitory binding, a stabilising binding, or a combination of the two, with one or more target proteins. Cases of some class I T3Cs provide examples of how the observed chaperone fold can regulate transcription through interactions with partner proteins, either by direct interaction with a transcriptional regulator (eg. *P*. *syringae* chaperone HrpG suppresses the negative regulator HrpV [59]), indirect interaction with a transcriptional regulator via a co-activator/antiactivator (e.g. *S*. *flixneri* Spa15 chaperone acts as a co-antiactivator in complex with OspD1 to sequester the transcriptional activator MxiE [57]), or to sequester an inhibitor of a transcriptional regulator (e.g., *P*. *aeruginosa* ExsC chaperone sequesters the antiactivator ExsD from the ExsD-ExsA complex, releasing and activating the transcriptional regulator ExsA [58]). In the case of AmyR, regulatory pathway proteins could be bound and sequestered in a dose dependent manner, explaining the observed variations between the wild-type, knockout and over expression *amyR* mutants [16].

Chaperone-like binding can also stabilise the target proteins, increasing their accumulation and subsequent activity. In mutation studies of the Enterobacteria *Yersinia*, *Salmonella* and *Shigella*, non-secreting mutants are shown to accumulate effector proteins in the cytoplasm. However, when the class I T3C associated with an effector protein is also knocked out the effector accumulation is reduced, despite equivalent transcript levels [55,76–82]. The inherent instability of the effectors in the cytoplasm appears to be caused by their chaperone binding region, the removal of which can stabilise the effector protein in the absence of the chaperone [83]. In the same manner AmyR could stabilise transcriptional regulatory pathway proteins or inhibitors, determining their abundance through association, and explaining the dose dependent transcriptional regulation observed for AmyR. Furthermore, the currently studied YbjN proteins are expressed in response to stress, and specific stabilization could have the dual effect of increasing the protein accumulation and protecting the target protein from stresses such as heat shock and radiation [15,28,29].

## Conclusions

AmyR from E. amylovora is a member of the YbjN family, which have been implicated in regulation of the stress-response in bacteria. The structure of AmyR was solved at 1.95 Å resolution, revealing a class I type III secretion (T3C) chaperone-like fold. Functional studies have previously shown AmyR as an essential regulator of virulence factors in *E*. *amylovora*. Class I T3C-type proteins such as AmyR are capable of forming very specific interactions with one or more partner proteins. Therefore, the crystal structure presented in this study points to a mechanism where AmyR could accomplish virulence regulation by either binding and blocking partner protein activity, or through the stabilisation of partner proteins promoting cytoplasmic accumulation. Knowledge of the fold, and consequently possible modes of action, of AmyR will guide future investigation into the virulence regulation mechanisms of *E*. *amylovora*.

The structure of AmyR is the first of a YbjN protein from Enterobacteriaceae, the most evolutionarily divergent and highly studied subgroup of YbjN proteins, and the first from the phylum Proteobacteria. The structures of YbjN proteins show exceptionally high conservation of tertiary structure, despite highly divergent primary structure. The conserved sequence features from YbjN proteins were identified and mapped to the AmyR structure. These revealed that only residues involved in the stabilisation of both the protein core and the dimerization interface were conserved, while surface exposed residues completely lacked similarity across the YbjN family. However, within closely related bacterial groupings there was very high sequence similarity even at the putative surface-exposed interaction sites, indicating that related YbjN proteins, such as those within the Enterobacteriaceae family, interact with homologous proteins.

Despite the shared fold with class I T3Cs, the current evidence suggests that YbjN proteins are not implicated in a T3SS associated role. This includes their genomic location not matching that of T3Cs, their presence in species lacking a T3SS, their classification as an independent protein family from T3Cs, the regulation of their expression being associated with stress rather than infection, their transcriptional regulation activity not being associated with T3SS genes, and specifically for AmyR, knockout mutants showing increased virulence rather than decreased. While a T3SS associated role cannot be ruled out for all YbjN proteins, at this time there is no evidence of YbjN proteins performing a T3SS associated role, despite their shared architecture with class I T3Cs. The presented structural and phylogenetic analyses of YbjN proteins provides a foundation for future studies into this poorly understood but increasingly important protein family.

## Supporting information

S1 Fig The two observed crystal forms of AmyR. (PDF)

S2 Fig MCoffee alignment of 100 YbjN protein sequences. (PDF)

S3 Fig ESPript alignment of 100 YbjN protein sequences. (PDF)
