# Supplementary figures and images for: The crystal structure of Erwinia amylovora AmyR, a member of the YbjN protein family, shows similarity to type III secretion chaperones but suggests different cellular functions
Source: PLoS One. 2017 Apr 20;12(4):e0176049. doi: 10.1371/journal.pone.0176049 (PMC5398634; doi:10.1371/journal.pone.0176049)

**S1 Fig.** The two observed crystal forms of AmyR.

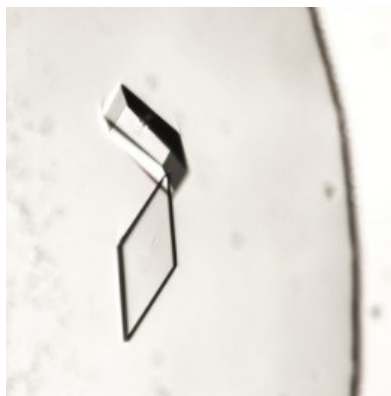

Supplement: S1 Fig — (PDF) [file pone.0176049.s001.pdf]
