# Supplementary material for: The crystal structure of Erwinia amylovora AmyR, a member of the YbjN protein family, shows similarity to type III secretion chaperones but suggests different cellular functions
Source: PLoS One. 2017 Apr 20;12(4):e0176049. doi: 10.1371/journal.pone.0176049 (PMC5398634; doi:10.1371/journal.pone.0176049)
Supplement: S2 Fig — (PDF) [file pone.0176049.s002.pdf]

BAD AVG GOOD
*

E.amylovora-Amy	:	22
D.radiodurans-D	:	40
S.elongatus-T11	:	34
E.coli-YbjN	:	22
gi|123253281|sp	:	55
gi|123129053|sp	:	46
gi|123323499|sp	:	48
gi|122544362|sp	:	50
gi|123736226|sp	:	51
gi|122459425|sp	:	51
gi|122351524|sp	:	51
gi|123004239|sp	:	49
gi|122476043|sp	:	49
gi|122295500|sp	:	49
gi|122403190|sp	:	49
gi|122257709|sp	:	49
gi|81415193|sp|	:	52
gi|81596955|sp|	:	48
gi|123557011|sp	:	50
gi|81709081|sp|	:	40
gi|123506214|sp	:	40
gi|123556836|sp	:	39
gi|81670584|sp|	:	41
gi|122663130|sp	:	39
gi|81771933|sp|	:	40
gi|123351931|sp	:	39
gi|81596544|sp|	:	25
gi|123608251|sp	:	22
gi|81708722|sp|	:	23
gi|298346581|re	:	49
gi|260576430|re	:	50
gi|296129402|re	:	45
gi|254464399|re	:	51
gi|269795609|re	:	48
gi|167648377|re	:	51
gi|302865994|re	:	48
gi|254474961|re	:	52
gi|238063407|re	:	48
gi|227495918|re	:	49
gi|269219692|re	:	49
gi|145593940|re	:	48
gi|269976471|re	:	49
gi|217969472|re	:	52
gi|399991304|re	:	51
gi|256832278|re	:	47
gi|225022571|re	:	48
gi|126738030|re	:	52
gi|254294753|re	:	52
gi|149184640|re	:	50
gi|257068239|re	:	49
gi|229820926|re	:	49
gi|227494902|re	:	49
gi|220934858|re	:	44
gi|291295614|re	:	45
gi|296130820|re	:	49
gi|84685465|ref	:	51
gi|229820927|re	:	47
gi|227496193|re	:	47
gi|154507942|re	:	50
gi|86137245|ref	:	51
gi|227494903|re	:	47
gi|152982702|re	:	50
gi|119486978|re	:	40
gi|145596051|re	:	47
gi|220903645|re	:	50
gi|297566041|re	:	51
gi|227496194|re	:	48
gi|119385461|re	:	48
gi|183220878|re	:	49
gi|291302096|re	:	46
gi|387905553|re	:	48
gi|238927566|re	:	40
gi|322420651|re	:	40
gi|225629038|re	:	49
gi|227495837|re	:	50
gi|291295390|re	:	49
gi|302381558|re	:	49
gi|229589918|re	:	49
gi|229917768|re	:	43
gi|304320687|re	:	49
gi|154508096|re	:	53
gi|172058831|re	:	43
gi|85705365|ref	:	41
gi|119512017|re	:	39
gi|256397448|re	:	43
gi|94985189|ref	:	60
gi|238927565|re	:	43
gi|304320540|re	:	50
gi|163846985|re	:	47
gi|121606820|re	:	36
gi|121583407|re	:	35
gi|167856646|re	:	46
gi|283780877|re	:	48
gi|85708893|ref	:	48
gi|304321485|re	:	48
gi|304321021|re	:	49
cons	:	47

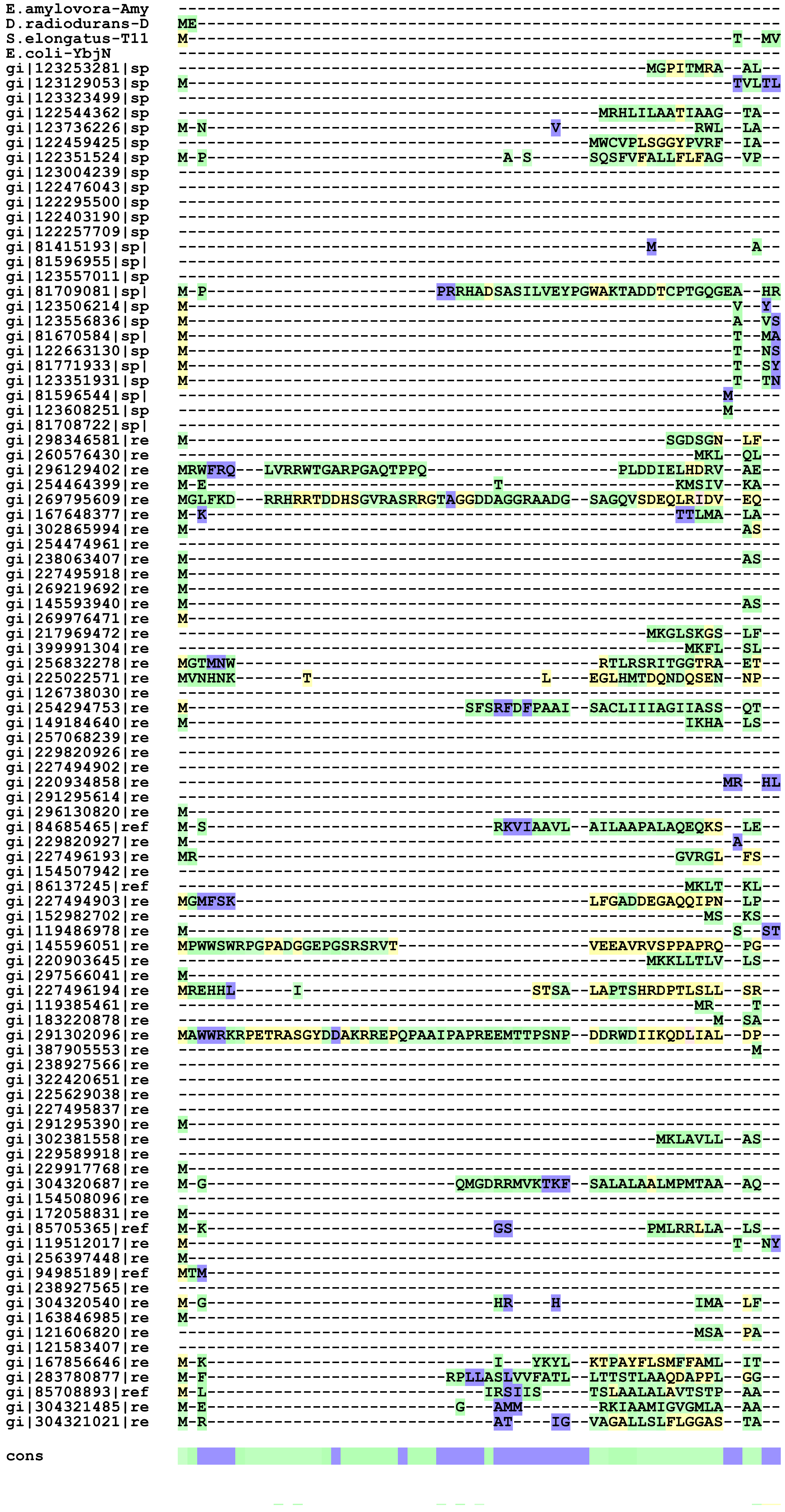

E.amylovora-Amy
D.radiodurans-D ME
S.elongatus-T11 M T MV
E.coli-YbjN
gi|123253281|sp MGPITMRA AL
gi|123129053|sp M TVLTL
gi|123323499|sp
gi|122544362|sp MRHLILAATIAAG TA
gi|123736226|sp M N V RWL LA
gi|122459425|sp MWCVPLSGGYPVRF IA
gi|122351524|sp M P A S SQSFVFALLFLFAG VP
gi|123004239|sp
gi|122476043|sp
gi|122295500|sp
gi|122403190|sp
gi|122257709|sp
gi|81415193|sp| M A
gi|81596955|sp|
gi|123557011|sp
gi|81709081|sp| M P PRRHADSASILVEYPGWAKTADDTCPTGQGEA HR
gi|123506214|sp M V Y
gi|123556836|sp M A VS
gi|81670584|sp| M T MA
gi|122663130|sp M T NS
gi|81771933|sp| M T SY
gi|123351931|sp M T TN
gi|81596544|sp| M
gi|123608251|sp M
gi|81708722|sp|
gi|298346581|re M SGDSGN LF
gi|260576430|re MKL QL
gi|296129402|re MRWFRQ LVRRWTGARPGAQTPPQ PLDDIELHDRV AE
gi|254464399|re M E T KMSIV KA
gi|269795609|re MGLFKD RRHRRTDDHSGVRASRRGTAGGDDAGGRAADG SAGQVSDEQLRIDV EQ
gi|167648377|re M K TTLMA LA
gi|302865994|re M AS
gi|254474961|re
gi|238063407|re M AS
gi|227495918|re M
gi|269219692|re M
gi|145593940|re M AS
gi|269976471|re M
gi|217969472|re MKGLSKGS LF
gi|399991304|re MKFL SL
gi|256832278|re MGTMNW RTLRSRITGGTRA ET
gi|225022571|re MVNHNK T L EGLHMTDQNDQSEN NP
gi|126738030|re
gi|254294753|re M SFSRFDFPAAI SACLIIIAGIIASS QT
gi|149184640|re M IKHA LS
gi|257068239|re
gi|229820926|re
gi|227494902|re
gi|220934858|re MR HL
gi|291295614|re
gi|296130820|re M
gi|84685465|ref M S RKVIAAVL AILAAPALAQEQKS LE
gi|229820927|re M A
gi|227496193|re MR GVRGL FS
gi|154507942|re
gi|86137245|ref MKLT KL
gi|227494903|re MGMFSK LFGADDEGAQQIPN LP
gi|152982702|re MS KS
gi|119486978|re M S ST
gi|145596051|re MPWWSWRPGPADGGEPGSRSRVT VEEAVRVSPPAPRQ PG
gi|220903645|re MKKLLTLV LS
gi|297566041|re M
gi|227496194|re MREHHL I STSA LAPTSHRDPTLSLL SR
gi|119385461|re MR T
gi|183220878|re M SA
gi|291302096|re MAWWRKRPETRASGYDDAKRREPQPAAIPAPREEMTTPSNP DDRWDIIKQDLIAL DP
gi|387905553|re M
gi|238927566|re
gi|322420651|re
gi|225629038|re
gi|227495837|re
gi|291295390|re M
gi|302381558|re MKLAVLL AS
gi|229589918|re
gi|229917768|re M
gi|304320687|re M G QMGDRRMVKTKF SALALAALMPMTAA AQ
gi|154508096|re
gi|172058831|re M
gi|85705365|ref M K GS PMLRRLLA LS
gi|119512017|re M T NY
gi|256397448|re M
gi|94985189|ref MTM
gi|238927565|re
gi|304320540|re M G HR H IMA LF
gi|163846985|re M
gi|121606820|re MSA PA
gi|121583407|re
gi|167856646|re M K I YKYL KTPAYFLSMFFAML IT
gi|283780877|re M F RPLLASLVVFATL LTTSTLAAQDAPPL GG
gi|85708893|ref M L IRSIIS TSLAALALAVTSTP AA
gi|304321485|re M E G AMM RKIAAMIGVGMLA AA
gi|304321021|re M R AT IG VAGALLSLFLGGAS TA
cons

```
E.amylovora-Amy     ----------M-H--------------M-V-S----------------------------LVV
D.radiodurans-D     -------------------------------T-----------------------------AL
S.elongatus-T11     SE-VQP-------VSP-------ASL---D-A----------------------------PLE
E.coli-YbjN         ---------------------------M-T-S----------------------------LVV
gi|123253281|sp     --------TL---AA--TALLWASPAHA-E-L----------------------------VNA
gi|123129053|sp     LG-A--------------AVLPAHADDS-R-L----------------------------LTS
gi|123323499|sp     ------------------------------------------------------------MRS
gi|122544362|sp     --------TM---AF--TPATAQSFSAS-R-T----------------------------LNS
gi|123736226|sp     --------AL---AA--GVMAPSVAEAA-T-T----------------------------VTA
gi|122459425|sp     --------TL---LA--CLCLAGASQAQ-T-L----------------------------VKP
gi|122351524|sp     --------QA---LA--VGEAAESPSGP-S-I----------------------------VST
gi|123004239|sp     -------------------------MSD-T-L----------------------------ISK
gi|122476043|sp     -------------------------MPD-T-N----------------------------IDK
gi|122295500|sp     -----------------------MIMSN-H-T----------------------------IAK
gi|122403190|sp     ------------------------MTEV-T-L----------------------------IET
gi|122257709|sp     ------------------------MNDI-T-L----------------------------IEH
gi|81415193|sp|     --------RL---FL--LALILVPALAQ-G-V----------------------------RTG
gi|81596955|sp|     ---------------------MMLTNDL-I-T----------------------------PDN
gi|123557011|sp     ---------------------MMLTNDL-I-T----------------------------SEN
gi|81709081|sp|     MT-T--------------HEEALSNA---E-L----------------------------HLA
gi|123506214|sp     ----SE-------PLIDRSPEGFPGL---M-E----------------------------EPP
gi|123556836|sp     SE-T--------------TLDSSALS---A-T----------------------------APL
gi|81670584|sp|     SP-T-------------PELANPTMD---E-M----------------------------TPA
gi|122663130|sp     TT-E---------------YTLPDNF---T-S----------------------------TEI
gi|81771933|sp|     QE-TLTN----------NECIDELIT---E-T----------------------------ASI
gi|123351931|sp     NP-ETVSVDN---ASIEEANINELTE---D-E----------------------------TSL
gi|81596544|sp|     ---------------------------------------------------------------
gi|123608251|sp     ---------------------------------------------------------------
gi|81708722|sp|     ---------------------------------------------------------------
gi|298346581|re     --------DN---PF--ESPFDSVPFSV-N-R----------------------------LPN
gi|260576430|re     --------FS---LV--LAATLAGP-AV-SGD----------------------------VLA
gi|296129402|re     --------LL---VR--ELGSAE--HAT-T-T----------------------------PSP
gi|254464399|re     --------IA---AC--VALVLPAAASA-Q-N----------------------------VVA
gi|269795609|re     --------VL---AR--ELGALVDSAPE-P-G----------------------------VTP
gi|167648377|re     --------AA---MA--LAAGGAAHAQA-I-H----------------------------NDG
gi|302865994|re     --------PE---IE--DGPDGPLAGHP-Q-E----------------------------LRP
gi|254474961|re     --------MT---AA--ALFALPGFAQA-Q-N----------------------------IVA
gi|238063407|re     --------PE---IE--DGPDGPLAGHP-G-A----------------------------LRP
gi|227495918|re     --------------G--FFSKDASTPSY-N-P----------------------------LAP
gi|269219692|re     --------------------AFVDNDGA-A-T----------------------------LAP
gi|145593940|re     --------PT----G--DIPDRLLGGYP-G-A----------------------------LSP
gi|269976471|re     --------------A--DYEEYFQSLER-N-I----------------------------VSP
gi|217969472|re     --------FC---AL--LSAAAGPAFAD-A-L----------------------------IDA
gi|399991304|re     --------LT---AA--ALFVLPGLGHA-E-N----------------------------ILA
gi|256832278|re     --------TV---TP--GRVHHQRHADA-S-V----------------------------PIP
gi|225022571|re     --------SI---SG--RVERIIPTSGE-D-V----------------------------VQP
gi|126738030|re     --------ML---AA--AIALSPVAVQA-Q-N----------------------------IVA
gi|254294753|re     --------SF---AQ--NTPASAERVRG-G-I----------------------------LDA
gi|149184640|re     --------GA---VI--AAGLLAASPLA-A-K----------------------------NIV
gi|257068239|re     ----------------------MGKDSA-D-D----------------------------LAP
gi|229820926|re     ------------------------------------------------------------MRA
gi|227494902|re     -----------------------MAKDK-D-L----------------------------VYP
gi|220934858|re     LPLVTA--LV---LS--LLLVLPAQADQ-T-I----------------------------ISS
gi|291295614|re     ----------------------MEMKRQ-H-Q----------------------------TVE
gi|296130820|re     --------GF---FT--KDTSVPLQPAP-P-A----------------------------LGP
gi|84685465|ref     --------SK---PL--DAPASVQAPMS-D-R----------------------------VIA
gi|229820927|re     ---G--------------TQRGSAARTS-G-G----------------------------VRP
gi|227496193|re     --------RA---RR--RDSGAASAPDA-Q-Q----------------------------VRP
gi|154507942|re     -------------------------------M----------------------------VRP
gi|86137245|ref     --------FA---VA--ALAISPVAVQA-E-N----------------------------LTA
gi|227494903|re     --------EE---AV--EEPRFQPAIPT-G-E----------------------------LAP
gi|152982702|re     --------TS---AA--AAAVAETTPAV-E-L----------------------------LNT
gi|119486978|re     TP-Q--N----------LEAISEDLE---P-E----------------------------TTI
gi|145596051|re     --------DN---YA--GVDRPPAVDAS-A-T----------------------------VAP
gi|220903645|re     --------LC---LT--CIFAAGAMADS-D-I----------------------------ITA
gi|297566041|re     ---------------------------Y-T-D----------------------------ASP
gi|227496194|re     --------FS---RP--DPTPRAGADSS-Q-V----------------------------LAP
gi|119385461|re     --------LV---PL--VLAALMQAPAA-QAQ----------------------------VMG
gi|183220878|re     --------QS---PK--GKESESASKSA-T-L----------------------------HHK
gi|291302096|re     --------DF---FN--DLEQLTDEVPT-Q-K----------------------------SAE
gi|387905553|re     --------EH---EQ--TNTADERAAHD-A-P----------------------------IEA
gi|238927566|re     ----------M-EGT--MGLFDGFQGDV-E-S----------------------------AGM
gi|322420651|re     -----------------------------M--------------------------------A
gi|225629038|re     ---------M---II--PRSLAPASVPE-Q-I----------------------------ILE
gi|227495837|re     ---------M---SS--STPTTSQVPVP-A-V----------------------------PSP
gi|291295390|re     ---------------------------D-T-V----------------------------LRE
gi|302381558|re     --------CA---VI--ASPAGAQTAAP-G-T----------------------------WKG
gi|229589918|re     ------------------------M--S-E-L----------------------------ITS
gi|229917768|re     ---------------------------------------------N---------------FE
gi|304320687|re     --------SN---AL--TAPDSLVSNPT-S-I----------------------------VPS
gi|154508096|re     -----------------MNVPYLVPEDE-V-T----------------------------PYP
gi|172058831|re     ---------------------------DQD-RNSKDTNDNKVTPINQNSQPETNENGVTAVQQ
gi|85705365|ref     --------LV---LL--ATPLTAQDTPP-A-P----------------------------EPP
gi|119512017|re     QE-TLPS--------------SEFNS---D-T----------------------------VSV
gi|256397448|re     ---------------------------A-N-------------------------------VE
gi|94985189|ref     -----------------------------E-T-----------------------------AL
gi|238927565|re     -----------------MGLFDGFSGSE-E-G----------------------------LKN
gi|304320540|re     --------AG---LL--LCAGSAAAEKR-E-V----------------------------HDT
gi|163846985|re     --------------------TGAQPEEL-V-Y----------------------------PAH
gi|121606820|re     --------LANKT-----KPENFAMSDF-I-P----------------------------ESD
gi|121583407|re     ------------------------MSIL-I-E----------------------------EKD
gi|167856646|re     --------TT---LV--STTAYAETKMV-E-V----------------------------YTK
gi|283780877|re     --------LI---GN--AGNGGGAGAAD-K-I----------------------------IEK
gi|85708893|ref     --------AQ---QA--PSQTQAQSARP-D-W----------------------------VGR
gi|304321485|re     --------PA---LA--LTQLSTDIDQQ-R-I----------------------------VET
gi|304321021|re     --------QS---ND--LTVEENPLIAG-G-V----------------------------VRS

cons
```

```
E.amylovora-Amy     PDLDVLRRWLDQQSIT-------WF----ECD---SC-QAL-H-L-P-HMQNFDGVFDAKIDL
D.radiodurans-D     LTLDTLAKYLQEKEVQ-------LD----IEENGG-----QR----F-IRM------------
S.elongatus-T11     NAVEIIETVISSLHQGDA---PLVG----QT-D--S----G---K--IWMF------------
E.coli-YbjN         PGLDTLRQWLDDLGMS-------FF----ECD---NC-QAL-H-L-P-HMQNFDGVFDAKIDL
gi|123253281|sp     ANPATIKAIVESQGWP-------AT----IVAK----EG-D-D-P-Y-IES------------
gi|123129053|sp     ATLADLQSIVVEEGYT-------IL----ST-G--N-D--G-E-V-SVRAK------------
gi|123323499|sp     VFLADLKAVVAQAGYT-------IS----SV-G--D-N--G-A-D-SVRGV------------
gi|122544362|sp     FDYDTLRATVTEIGGT----I---E----PGQN---------D-G-F-LIK------------
gi|123736226|sp     IRPDGVAEALTNLGYT----A---E----LAKD----AN-G-D-P-L-INA------------
gi|122459425|sp     GEVDQIVDIARGFGSG--------T----LEKD----E----T-E-Y-VRG------------
gi|122351524|sp     NEVDAIRELAEGYGDA----R---V----VELD-----N-G-DP--A-IVG------------
gi|123004239|sp     LDLDGLRELFQAAGYR----V---E----TASD--P-VA-S-L-T-Y-LRS------------
gi|122476043|sp     LSLDSLREVLQQAGYR----V---E----TVTD--P-IA-N-V-A-Y-LRS------------
gi|122295500|sp     MSLGGLRDIFQQAGYR----V---E----TVTD--P-VA-N-V-D-Y-LRS------------
gi|122403190|sp     VSADSLTKLLQDAGCR----V---N----RSEQ--N----A-V-V-Q-LLS------------
gi|122257709|sp     LSVDSLTEALQEAGYR----V---N----RSEQ--N----G-V-V-Q-LLS------------
gi|81415193|sp|     ITPGEMEALLKAGSYR-------YE----RVEE----G--G-R-V-Y-FHL------------
gi|81596955|sp|     VSKDLLKAIYDAAFIE-------TA----WDDD-G------D------LKI------------
gi|123557011|sp     VSKDLLKAIYDAAFME-------TA----WDDD-G------D------LKI------------
gi|81709081|sp|     THREEVEAVIASLAMEGSVRFAQLK----ES-D--K----G---L--MWIF------------
gi|123506214|sp     RYVEVVSAVVSSLKED-A---AY-E----NH-E--Q----G---H--TWKF------------
gi|123556836|sp     DYIDIIETVISSLADADS---AQVS----HT-G--S----G---T--IWKF------------
gi|81670584|sp|     SHHDVVETVISGMAQENS---AFVQ----DN-D--Q----G---S--IWKF------------
gi|122663130|sp     SHQEAIETVIDSLQENDS---AMVH----HD-E--Q----G---Y--LWKF------------
gi|81771933|sp|     NHVEVIENVIDSLEQDDS---AMVS----HTPE--G----G---Y--LWKF------------
gi|123351931|sp     GYIENIETVIAGMAEEQK---VMVA----QN-E--A----G---H--LWKF------------
gi|81596544|sp|     -QVAEISPLLIELFGAD-----RLE----AN-P--P----E---S---WQI------------
gi|123608251|sp     -TPEVIAQTLAELFSTA-----DVQ----AI-A--P----G---S---WQV------------
gi|81708722|sp|     -------------MAGG-----TLS----EM-A--P----G---Q---WQI------------
gi|298346581|re     LTRDRVKKVLESHKWS-------YQ----VNHD-G------D------VGG------------
gi|260576430|re     DNPAAIAELMRNFGYR-------AN----LGTD----DQ-G-D-P-K-IDS------------
gi|296129402|re     VSPARIASWMSDNQFS-------YF----IDND-G------D------LGG------------
gi|254464399|re     STGDSVADFFKDEGAK-------VE----LTTD----SV-G-D-P-N-VKV------------
gi|269795609|re     VTRARVIEWITDYGYS-------YF----VDSD-G------D------VGG------------
gi|167648377|re     MTGPEVAAWLQKGGYK----A---E----LTKD----DG-G-D-P-L-INS------------
gi|302865994|re     LTGELIAAVLGHRGYA-------VV----EEPD-G------A------LVG------------
gi|254474961|re     KNATSIANFFDEEGVA-------FE----VTTD----DV-G-D-P-K-IKV------------
gi|238063407|re     LTDELIAAVLRHRGVT-------VA----ADPV-G------R------LVG------------
gi|227495918|re     LSLDRVAKVLEDDELK-------YG----YDED-G------D------LAC------------
gi|269219692|re     LSQERIVAALERDKII-------YA----YDSD-N------D------LAA------------
gi|145593940|re     LSGELIATVLTHRGYL-------VE----TDPA-G------E------LVG------------
gi|269976471|re     LNRARVKKILERNGWS-------YQ----VGNE-G------D------IAG------------
gi|217969472|re     TKPERLYEIARGFGSA--------E----LDKD----SQ-G-D-P-R-ITG------------
gi|399991304|re     KDATTLARFFETEGVE-------FE----VTTD----DV-G-D-P-K-LKV------------
gi|256832278|re     LSQDHITTWLTAHDLP-------YF----IDSD-G------T------AGA------------
gi|225022571|re     LSADRIAAYLKAKELM-------FN----RDDD-G------D------VFI------------
gi|126738030|re     TTAYSAKSFFENEGAE-------VE----ATTD----NV-G-D-P-K-LKV------------
gi|254294753|re     SDANRLSRFMTSLGYQ----A---D----MATG----PA-G-D-P-I-ISG------------
gi|149184640|re     ADVDQIANLLRAEGYK----A---K----VEGE----G--G-D-R-H-IKT------------
gi|257068239|re     LSLTRVEESLTRHGYA-------FV----EDEE-----H-P---E-I-LRAR-----------
gi|229820926|re     VDRKRVQTALDALGYR-------YF----VDSA-G------E------VGG------------
gi|227494902|re     LSKERLQALFEAEGYK-------YY----VDSD-G------D------LGG------------
gi|220934858|re     TNAKQVEGLLKELGFTG-----TRI----DE-D--D--------D---VIV------------
gi|291295614|re     FNRALIRQYVEERGLR-------YL----TDQD----GD-Y-V-A---IMA------------
gi|296130820|re     LSRDRITGALDARDMQ-------YG----IDDD-G------D------VGG------------
gi|84685465|ref     SDPQTFVAFFEDAGMP-------AR----LTED----TV-G-D-P-L-VEY------------
gi|229820927|re     VTSERLEDHLRDRRLL-------VS----RR-E--D----G-V-----LLG------------
gi|227496193|re     LTRERVQELLERHGWV-------YE----TDCD-G------D------LVG------------
gi|154507942|re     ITQERLGLLFDSEGWA-------WR----IDSDG-------D------LCG------------
gi|86137245|ref     RHSSSIEGFFLDEGSS-------VE----VLVD----NV-G-D-P-Q-LNV------------
gi|227494903|re     LTLERVTQHFDEEGYN-------YG----IIDADPA-E--GSR-A-K-IES------------
gi|152982702|re     VNAEQVSDAIKAAGCA----V---T----TTEQ--D----G-T-T-W-LHS------------
gi|119486978|re     TYTEEIETVIVGLAKDQK---VMVG----QS-D--A----G---H--LWKF------------
gi|145596051|re     VTLSRICAALDGLDVR-------YL----ADGD-------G---N---LLAM-----------
gi|220903645|re     SDQEKILEIAKGFG-S-------AE----LSST----SK-G-T-P-L-IQG------------
gi|297566041|re     LTADELRVVLDQLHYQ-------VE----RLEE----D--R-F-S-I-V--Y-----------
gi|227496194|re     LSQDRLLALFKERDWR-------YF----IDSD-G------D------LGG------------
gi|119385461|re     D-PEVIRLMMMDFGLP-------TK----LTTD----AA-G-D-P-L-IES------------
gi|183220878|re     VEKNLIKKLLVELGYQ-------II----S--D----ED---K-L---LIV------------
gi|291302096|re     LTDERLIASLRRLNIR-------YL----VDEA-------G---A---LLAM-----------
gi|387905553|re     VSAERLAEILRRAGYR----V---T----VAEQ--N----G-A-M-Q-LMS------------
gi|238927566|re     GIRKAIRAELDRNEWN-------YE----ESEE--SDETRK-E-A-Y-FYMRNE---------
gi|322420651|re     QNALAFEEYLKSQEIS-------LEK---NTHE-----D-N---T-M-YVIR-----------
gi|225629038|re     FSMPTLLQFLQGEGWC----V---R----PDQH----SL---F-P-Q-IIA------------
gi|227495837|re     VDVDRVLACVRDLGLR-------YF----VDDE-G------D------IGI------------
gi|291295390|re     VTTEEVRILLDQFGLN-------AE----QVKE----D--L-F-N-L-E--L-----------
gi|302381558|re     LTVAEITGQLTASGLV----V---E----APQ-----AQ-G-D-RVY-IPV------------
gi|229589918|re     VTTQRLTELLQEAGYR----V---N----PSEQ--N----G-V-V-Q-LLS------------
gi|229917768|re     EKVESFRALAAERNMT-------FK----ESDT----E--T-H-V-T-FLTR-----------
gi|304320687|re     LHAEALLPILDELGLD----Y---Q----GATL----PD-G-R-RII-LAA------------
gi|154508096|re     ADFERVVQAVREMGYA----L---D----VIEK---------GR--A-AGA------------
gi|172058831|re     QYLEEFQAFLVEKGIP-------ME----AREN----E--T-H-V-F-FMTQ-----------
gi|85705365|ref     MTLERLDTIIRALDPE----A---E-------SN-----G-S-----V-WQL------------
gi|119512017|re     NHVEVIENVIGTLEQDDS---AMVS----HNPK--D----G---Y--LWKF------------
gi|256397448|re     MIRAFIARVLEDGGLV-------DG----RAEA--D-SD-G-D-Y-Q-L--R-----------
gi|94985189|ref     LTLDTLAKYLREKEVQ-------LD----IEENNG-----QR----F-IRM------------
gi|238927565|re     SAYEAIKAEFEKQGLN-------YG----ED----TSEDGE-D-H-I-LRMRQQ---------
gi|304320540|re     ISAEELAAALTAAGLA----P---T----VLTD--R-AT-G-A-P-V-VTG------------
gi|163846985|re     VNRAWLFQLYNDAYFD-------VE----LDSD-G------D------VCL------------
gi|121606820|re     VTPVTLSLHLERAVVQ-------HR----L------EDDEA-----I-YVTED----------
gi|121583407|re     VTAVNLAVELEQAVIG-------HV----L------DEDKS-----I-YVTED----------
gi|167856646|re     FSDQQLIDIVKKKYPK-------VE----LIEK----GR-I-R-----ITS------------
gi|283780877|re     LSPDVVIEVLKAEGYK----D---LQHVPSTKE-----D-S-P-D-A-IFV------------
gi|85708893|ref     FDDQLMRMLMQDLRAT----W---T----VEQS----AE-G-LTT-Y-RAS------------
gi|304321485|re     LSFGDILPVLQRYGVN----Y---Q----VGDM----G--G-R-P-V-AKA------------
gi|304321021|re     LDLDQLSEAIATAGFL----P---T----ISHS----AT-G-L-PYI-VAE------------

cons
```

```
E.amylovora-Amy     MDGVI-------L------------------------------------FSA-----LA----E
D.radiodurans-D     ----GWRFEM--G----D--AAV-LVSV-NDGP--NNTS--R-----LEITC----VT----Q
S.elongatus-T11     ----R----Y--G----S--AEV-FVQL-SGHT--E--EDF------LTIWS----PV-----
E.coli-YbjN         IDNTI-------L------------------------------------FSA-----MA----E
gi|123253281|sp     ----S----R--N----G--LKF-LVLF-MNCE--E--G--Q-----KCKTLQYYM-------
gi|123129053|sp     ----T----A--E----G--LVF-NVIG-TVCD--S--ENAD-----GCLGI----NM----Q
gi|123323499|sp     ----T----A--D----G--LIF-NVDG-AVCE--N--EIRP-----GCLGI----NI----N
gi|122544362|sp     ----F----P--N----G--TAA-TATF-TAC--AT-----G-----KCLGT----NI----S
gi|123736226|sp     ----D----I--G----G--WQA-ALMF-YECN--E--KT-H---D-GCQSL----QF----V
gi|122459425|sp     ----R----I--D----G--TLY-VIFF-NDCNS-D--R--S-----ECQSLQFYA-------
gi|122351524|sp     ----D----I--N----G--TAY-QLFF-LECE--D--G--K-----DCGAL----NFYAIW-
gi|123004239|sp     ----A----T--N----G--LAF-DIRP-GNRLSDG-----Q-----GIIDI----AL----V
gi|122476043|sp     ----A----T--N----G--LAF-DIRA-GNRLADG--S--A-----EFVDI----AF----T
gi|122295500|sp     ----A----T--G----G--LAF-DIRP-GNRLSAA--D--D-----SFVDV----AF----T
gi|122403190|sp     ----A----S--Q----G--VGY-AVRF-GNRAQGQ--E--G-----EFLDF----TF----S
gi|122257709|sp     ----A----S--Q----G--IGY-AVRF-GNPA-AT--P--A-----SYLDF----TF----S
gi|81415193|sp|     ----R----L--A----G--LRA-VLFL-QDCR--E--G--S------CESLLLYA-------
gi|81596955|sp|     ---------K--D----E--LNC-TVMI-S-EN--Q--T--R-----IMIFT----IF----E
gi|123557011|sp     ---------N--E----D--IGC-SVFI-S-DN--Q--E--K-----ITFLR----VF----R
gi|81709081|sp|     ----K----Y--G----T--VRV-FVVM-SGES--N--DDT------LTVWA----PV-----
gi|123506214|sp     ----T----Y--G----S--VEV-FVHL-SGES--P--EDT------LLVWS----PV-----
gi|123556836|sp     ----R----Y--G----Q--LEV-FVQL-TGSS--E--DDL------FTVWA----KV-----
gi|81670584|sp|     ----A----Y--G----S--VEV-LVQL-TGEG--E--NDL------FRVWA----EV-----
gi|122663130|sp     ----Q----Y--G----S--VET-YVQL-TGEK--E--EDL------LTVWS----PV-----
gi|81771933|sp|     ----K----Y--G----T--VEV-FVQL-TGKS--D--EDT------ITVWS----VV-----
gi|123351931|sp     ----K----Y--G----S--VDV-YVRL-TGET--D--DDN------LTVWS----YV-----
gi|81596544|sp|     ----Q----T--P----E--CRL-LLLL-SASG------EW------LRVLL----PL-----
gi|123608251|sp     ----D----T--S----T--FRL-LVLL-SEDS------SW------LRVLL----PI-----
gi|81708722|sp|     ----E----T--G----Q--WRL-LVLT-SVER------DW------LRMMI----PL-----
gi|298346581|re     ----A----W--Q----N--GIY-YFQV-TGEK--D--T--I-----LCVRG----TW----R
gi|260576430|re     ----S----S--G----G--SNF-SIYF-YGCT--N--G--K-----ACDSIQFSS-------
gi|296129402|re     ----L----W--R----G--RLF-YFFL-FGQQ--A--E--I-----LQVRG----QW----H
gi|254464399|re     ----E----Y--Y----G--SDF-SVYY-YGCG--N--N--T-----DCSAIQFFS-------
gi|269795609|re     ----L----W--H----S--RLF-YFLL-FGAQ--T--E--I-----LQVRG----QW----N
gi|167648377|re     ----A----A--E----G--QTF-KIYF-YDCK--D--AR--------CKAL----QF----S
gi|302865994|re     ----R----W--E----R--NLI-WFHR-RGVA--G--E--L-----LQVRT----VV----A
gi|254474961|re     ----D----Y--Y----G--NDF-SIYF-YGCT--D--N--T-----NCDAIQFFS-------
gi|238063407|re     ----R----W--D----D--GLI-WFLR-IGAA--G--E--I-----LQVRT----IA----A
gi|227495918|re     ----G----W--D----T--GIF-FFMR-GGEQ--G--E--I-----LRIQG----RF----H
gi|269219692|re     ----G----W--E----H--GGF-FFLI-GGES--D--E--Y-----LRVQG----RW----Y
gi|145593940|re     ----R----W--A----D--SLI-WFLR-PGTA--G--E--L-----LQVRT----VV----A
gi|269976471|re     ----A----W--E----N--GIY-HFEV-TGNR--D--S--V-----LCVRG----TW----R
gi|217969472|re     ----R----I--E----G--TKY-GIYF-YGCV--K--G--A-----DCDDIQFSA-------
gi|399991304|re     ----D----Y--Y----G--NDF-SIYF-YGCE--N--N--K-----NCDAIQFFS-------
gi|256832278|re     ----I----H--T----A--RIF-TFAL-IGQH--K--E--V-----LHIRG----RW----N
gi|225022571|re     ----N----F--D----G--NGF-VLLA-TGEK--R--E--I-----LVVRG----AW----Q
gi|126738030|re     ----N----Y--Y----G--NEF-SVYY-YGCD--N--N--S-----DCNAVQFFS-------
gi|254294753|re     ----R----I--S----S--SDY-TIHF-YECE--N--GE-F------CNSI----QF----L
gi|149184640|re     ----G----M--A----G--YSF-LILP-YDCN--D--KG-D---A--CKSV----QF----Y
gi|257068239|re     ---------F--D----D--YRF-QFMV-SGDE--H--G--V-----LQTRG----RW-----
gi|229820926|re     ----L----W--D----N--RLF-SFYL-IGSG--A--Q--M-----LQVRG----RW----P
gi|227494902|re     ----F----W--D----F--NTF-HFVL-AGEN--Q--E--L-----LHILC----RS----R
gi|220934858|re     ----M----M--H----G--RPV-VVIVGSGNG------RQ------LQMNV----AF-----
gi|291295614|re     ----M----E--H----CPQKLF---TI-FAAS--G--L--R-----E-DVFSITMRV----E
gi|296130820|re     ----Y----W--D----G--HLF-YFFL-LGGN--G--E--F-----LQTRG----RW----N
gi|84685465|ref     ----R----S--N----G--DKM-SLFF-YDCE--D--N--V-----DCQAVQFYA-------
gi|229820927|re     ----E----W--Q----D--MPF-SIGL-GGRE--D--SVL-------QVRG----RW----P
gi|227496193|re     ----R----W--D----D--DLL-TFMV-RGES--G--E--M-----LNVMG----YM----L
gi|154507942|re     ----F----W--E----G--HLF-CFRF-LGDS--R--E--V-----LSIVA----FM----K
gi|86137245|ref     ----E----H--Y----G--SEF-TIFY-YGCE--D--N--T-----NCDSIQFYS-------
gi|227494903|re     ----G----W--D----G--IPF-LFNF-NGQE--G--E--I-----LTVFA----QS----S
gi|152982702|re     ----A----S--H----G--IGF-QVLW-GNA--IT--P--G-----QYADL----TL----S
gi|119486978|re     ----Q----Y--G----S--AEV-FVQL-TGDT--E--DDT------LTVWS----PV-----
gi|145596051|re     ---------W--E----R--HAV-LVAL-EGPD--D--E--I-----LVLRV----RP-----
gi|220903645|re     ----R----M--D----G--IKY-AIWF-SGCT--D--G--K-----SCSALQFIG-------
gi|297566041|re     ---------T--T----G--LKA-SLVT-YGEP--S--K--L-------RSL----QL----R
gi|227496194|re     ----I----W--D----D--ATF-YFNL-GGQK--Q--E--V-----LRIWA----QL----P
gi|119385461|re     ----R----I--D----G--TSF-QVYF-YQCE--------K-----TCAAIQFSA-------
gi|183220878|re     ----S----Y--Q----D--FRV-GLVS-GN----------------DTSIQFYS-------
gi|291302096|re     ---------W--E----R--HVV-QIRR-EGPA--G--E--I-----LVLRA----RA-----
gi|387905553|re     ----A----S--Q----G--VGF-AVRF-GNPAVGV-PPA-IDAARIVYLDY----TL----S
gi|238927566|re     --LEN----D--E----P--VTV-VIAV-REYN--H--A--D-----GFIKI----KIYD--I
gi|322420651|re     ---------DNLE-EVGP--VSL-MALF-NDSD--R--Y--V-----T-LIC----YK-----
gi|225629038|re     ----S----L--P----D--VEC-IIRF-GTLA--R--SG-T---G--WSDF----TL----S
gi|227495837|re     ----P----W--R----Y--VTV-HVIF---QD--D--R--A-----IQLRG----VW----H
gi|291295390|re     ---------P--N----G--LRG-HLIT-YGG---S--H--V--------SSL----QL----R
gi|302381558|re     ----T----D--G----A--VRS-IVTL-FSCS--D--G--A------CPDV----QF----T
gi|229589918|re     ----A----S--Q----G--IGF-AVRF-GNPA-AE--Q--G-----SYVDF----TY----S
gi|229917768|re     ---------E--TTKSGA--QPVMIIAF-NKKT--T--D--A-----E-LYA----AT----V
gi|304320687|re     ----A----P--N----G--LKF-QLTP-TACE--N--NN-K-----RCRGL----HL----L
gi|154508096|re     ----I----F--D----E--IPF-LVSF-D--A--A--GR-F-----LSIRA----LWESDL-
gi|172058831|re     ---------E--KTPSGA--EPMMMIVF-SKTA--T--D--V-----E-LFA----RT----I
gi|85705365|ref     ----T----V--N----D--VQV-LIVT-DESA--D--R-------------M-------RAMTPVA
gi|119512017|re     ----K----Y--G----S--VEV-FIQL-TGES--D--EDT------ITVWS----AV-----
gi|256397448|re     ---------S--G----S--ALY-WVSA-GE-------A--D-------DGL----GLVQV-F
gi|94985189|ref     ----GWRFEM--G----D--AAV-LISV-NDGP--NNTS--R-----LEITC----VT----Q
gi|238927565|re     --LDN----G--S----V--VSI-AIVV-TENG--D--T--N-----DFIKI----KYFG--M
gi|304320540|re     ----I----S--D----G--LVF-VVRA-LECS--G--LP-A---R--CEQL----VL----F
gi|163846985|re     ---------R--Q----G--FRV-WVFP-I-RE--G--E--Q-----IRFMS----QF----R
gi|121606820|re     ---DW---------------IPF-WIRI-L-----K--K--P-----GFVGF----VTYF--N
gi|121583407|re     ---GW---------------FPF-WIRI-Q-----E--S--A-----GYVTF----KTYT--N
gi|167856646|re     ---------D--S----G--IKL-V--I-YNQR--------D------DGSLTFRM-------
gi|283780877|re     ----T----Y--E----G--RRV-LVVV-P-----N--G---------GDLL----QV----N
gi|85708893|ref     ----A----E--G----G--INF-VAVP-RSCE--D--Q--N-----GCVGL----VVIALFN
gi|304321485|re     ----Y----M--G----G--TEV-TLVP-NVCT--A--DH-R------CAGL----SL----Y
gi|304321021|re     ----K----A--N----G--ARL-FATL-GACDMPQ--AG-K---G--CQLI----EY----L

cons
```

```
E.amylovora-Amy    -VK--P-----T---ALIPLAGD-LSQINASSLTVKAFL--D-----I---QD------DNLP
D.radiodurans-D    -K---TY----AD---RRAEVAMM-LNDRNRERAFARS-I-D------Q-EG--------NV--
S.elongatus-T11    LP-LP-----VA---DELALYRK-LLTLNWLTTFEAHFA--I-----A---EE------QV--
E.coli-YbjN        -VR--P-----S---AVLPLAAD-LSAINASSLTVKAFL--D-----M---QD------DNLP
gi|123253281|sp    -----GFS----D---AKDMPLER-FNQWNREKRFARAYK-------DD---AGD------P--
gi|123129053|sp    VR-Y------DA---DGKETLER-INDVNLMWAATSAWYSVG-----G----TDGK-T-PTV--
gi|123323499|sp    VR-Y-------DG---DDRVTYQK-INDANLMWPAVSVSV-EG---------NMGE-TGSTV--
gi|122544362|sp    -A--RFGKPSDK---TDSQVAEL-VRDYNRRWSAGKSYL--T-----D---DG------RA--
gi|123736226|sp    -A--NFT----P---EKKFTAED-AVKFMRNTRFASVSL--T-----Q---DQ------SV--
gi|122459425|sp    -----SWD--------GVPVDQAA-INGWNRDKRFAKAYV-------DA---END------P--
gi|122351524|sp    ------D--------VPTVSVGA-LNVWNRTAPFNKAYL--T-----E---EN------RP--
gi|123004239|sp    -A--VIQ----V---QGELPLAI-VNRWNASRRFGRLQL--S---------GP------FL--
gi|122476043|sp    -A--ILQ----V---QGDLPLDL-VNRWNASRRFARLQL--S---------QP------FL--
gi|122295500|sp    -A--VLQ----I---QGELPLDL-VNRWNATRRFARLQV--S---------QP------FL--
gi|122403190|sp    -C--ALR----I---QGELPAGL-AERWNASRRFARLSV--Q---------GE------FL--
gi|122257709|sp    -C--ALR----V---QGELPAGL-TERWNLSRRFGRLSQ--Q---------GE------FL--
gi|81415193|sp|    ----GFS----T---DNPPSLER-VNEWNREKRFSRAYL-------DE---EGD------P--
gi|81596955|sp|    -F--EEG----F---SREQKLDL-ANKINDEYAFVRASL-T------E-YD--------TL--
gi|123557011|sp    -F--EEG----V---SRADRLEF-VNRINDKYSFVRAML-T------T-YD--------TL--
gi|81709081|sp|    MK-LPEN----SA---KTGELYAH-LLRSNWLETWEARFC--L-----R---EN------EV--
gi|123506214|sp    LS-LP-----VA---EPAKLMQK-LLEKNWSETLEARFC--I-----W---NN------QV--
gi|123556836|sp    FP-LS-----ES---TDATLLQR-LLTLNWADTLEARFA--L-----Y---DD------HI--
gi|81670584|sp|    MP-LP------T---DPGQLLAE-VMQLNWSDTFEACFA--L-----R---EN------HL--
gi|122663130|sp    LT-LP-----AQ---DELGLMRK-LLEMNGEETLETRFG--I-----M---NN------QI--
gi|81771933|sp|    LK-LP-----AK---NEPKLLRH-LLELNCSSTFEARFG--I-----I---ED------QV--
gi|123351931|sp    LK-LP-----AK---NEPELMRK-LLQMNWLSTMESHFA--I-----V---DN------QI--
gi|81596544|sp|    LP-AV----------DAAPFHRQ-ILEANFDATGPVRHA--L-----H---QN------VL--
gi|123608251|sp    VP-LQ----------EAQAFLAQ-FLEANFDDTQEVRYA--L-----Y---DG------VI--
gi|81708722|sp|    VP-QE----------EAMPLAAQ-LLEANFDRTREARFA--F-----Q---NG------LL--
gi|298346581|re    -G--EPE---LD---DFILINSI-CNRWNTEYYWPKAYA-R------V-TE-DRE-V--YV--
gi|260576430|re    ----GFD----L---TKGSSLQV-VNDWNRDKRYGKAYL-------DN---EMD------P--
gi|296129402|re    -R--ELA---IE---RLEEVLDL-CNEWNAERIWPKAYV-R------V-RD-NGR-V--HV--
gi|254464399|re    ----GYQ----T---DGSVRLAK-INEWNTENRFARGYI-------SE---EGA------A--
gi|269795609|re    -R--EIT---IE---RIEEVLDF-CNDWNTDRIWPKAYF-R------V-RD-NGM-I--QV--
gi|167648377|re    -A--GFD----L---KEPLKYEK-INEWNRKNRYLKAYL--D-----D---DG------DP--
gi|302865994|re    -D--HFG---IE---RVPELHAF-CNTWNHDRLWPKAYV-H------V-AD-DGS-A--QV--
gi|254474961|re    ----GYQ----T---DGGVRLSK-VNEWNTENRFGRAYI-------SE---EGS------A--
gi|238063407|re    -P--TFP---IE---YVPTLHAF-CNAWNHDRYWPKAFV-H------V-DD-DGR-A--LV--
gi|227495918|re    -L--PLP---AD---ELPKAMQA-CNDWNTRALWPKTFA-A------L-NN-ADQ-T--VV--
gi|269219692|re    -A--SLS---TD---RLGEAIQA-CNEWNIHTIWPKTYA-V------A-ND-VNE-V--VI--
gi|145593940|re    -P--TFP---IE---YVPALHAF-CNAWNHDRLWPKAFV-H------V-ED-DGR-A--RV--
gi|269976471|re    -G--KLE---LD---DFMLVSSL-CNRWNTEYYWPKTYA-R------V-TD-NRE-L--FV--
gi|217969472|re    ----SWS--------GPKVSLEK-INDWNRTKRFGKAYL-------DK---DGD------P--
gi|399991304|re    ----GYQ----T---DGGVRVVK-INEWNTENRFGRAYI-------SD---EGA------A--
gi|256832278|re    -R--HAT---IE---RAEQIRAL-CNDWNTTKIWPKAYH-R------V-RD-DGI-I--EV--
gi|225022571|re    -V--TAP---IE---MRDRLVDL-CNDWNRDKLWPKTYV-T------V-DD-AGA-V--RV--
gi|126738030|re    ----GYK----T---DGSVRLSK-INTWNAENRYARAYI-------SD---SGA------A--
gi|254294753|re    -A--DTP----I---PPAMTMEK-VNAFNARWRYVRASL--T-----S----N------VV--
gi|149184640|re    -K--AFT----P---KDKPTLEE-MNSYAAENRFGRIYL--D-----Q---DR------DP--
gi|257068239|re    SH--SVD---VS---RKVQMVKL-CNEWNMNRIWPKVYV-R------R-E-SEGL-L--GV--
gi|229820926|re    -R--RVA---LE---RLGAMLEF-TDAWNRERIFPKCYV-R------V-LD-DGM-V--HV--
gi|227494902|re    -R--VLT---MK---YLDVVRAK-IKEFNAEKIFPTCFY-R------I-SD-EGL-L--TV--
gi|220934858|re    V--------------GMGITLEQ-VNAWNRNRILAKAYL--D-----R---DG------DT--
gi|291295614|re    -P--SPE----M---SELEALRL-VNRWNTQRRWPRAYH-------TE---DG-------F--
gi|296130820|re    -R--KVG---PD---QLGRLLEL-VNEWNATHLWPKGYV-R------L-ED-E-V-V--GV--
gi|84685465|ref    ----GYR----A---EN-VTLET-INSWNTDRRFVRSYL-------TD---EGV------A--
gi|229820927|re    RL-LP-----AR---AGAGLAQL-INDWNRDRVLPKVYS--V-----P---EGDA-V--AV--
gi|227496193|re    -E--DLP---LE---RLDEVRFA-LEDWHREHIWPTCFW-R------E-ND-DALTF--SV--
gi|154507942|re    -S--LVP---IE---CGEDLRDF-LQAWHGEFLWPKAYV-A------D-QD-EG--D--RV--
gi|86137245|ref    ----GYA----T---DGSVRLKT-VNDFNAEKRWVRAYV-------AD---NGS------T--
gi|227494903|re    -V--DIP---LD---AEDQLDAF-IENWHREHYFPKVYT-R------R-ASADTA-L--RV--
gi|152982702|re    -C--PLR---VQ---GGVLPEGV-LTEWHRTKRFARVAL--H---------GD------FV--
gi|119486978|re    LQ-LP-----AK---NEATLMRK-LLEMNWLSTYESHFA--I-----N---NQ------QV--
gi|145596051|re    HA--TVP---PD---WEDRAYRV-VNEWNHTRRFCKAYI--G-----D-PSERGQ-L--PI--
gi|220903645|re    ----MWK----T---E-DFPMAE-VNKWNAQKVYTRTYL-------DK---DQD------L--
gi|297566041|re    -A--GFT---GF---NRIE-LRH-INTWNRRYRFSKAYL--D-----G---DN------DP--
gi|227496194|re    -G--TID---AS---HLEQVRTV-LDTSHRRSAWPTACY-R------I-DD-DGE-V--RV--
gi|119385461|re    ----GFD----L---DDSMSESM-ANRWNREKRFGKVWL-------DE---TGD------P--
gi|183220878|re    ----SFN----T---DKKNKMEL-ANKWNQKMRYSRSYM-------DA---EGR------L--
gi|291302096|re    YS--TLP---AE---WNDRAFRA-VNEWNRTRRFLKAYV--G-----E-PTESGS-V--PL--
gi|387905553|re    -C--VLQ----V---QGELPAEL-VANWNRTKRFARLAS--H---------GA------FL--
gi|238927566|re    -AYLEES----S---NRAELLAK-LNGWNAEYRYVKFCL--D-----S---DQ------DV--
gi|322420651|re    YF--AFP---AE---KKPAVLEM-INTLNAEYTMVK-YV--E-----T-G-N-------AL--
gi|225629038|re    -A--PFI--------VDSEVSPFIGALWNRKNRFGRVYR--I-----N---K-------SL--
gi|227495837|re    -R--ISD---TE---HLARLRGL-VEEWNATRIGPKAYL-T------I-AD-GGI-V--RL--
gi|291295390|re    -A--GFS---GF---DRVEPRHL-LN-WNRRYRFSKAYF--D-----A---DN------DP--
gi|302381558|re    -A--AFA--------GPQATPDI-VTRWNAERRFVKAFY--AVSGEAE----EG------QA--
gi|229589918|re    -C--ALR----I---QGELPEGL-AQVWNASRRFARLSV--Q---------GE------FL--
gi|229917768|re    -T--HVPD--YV---EDLPILNA-LNEFNQEFKYFRCVL--D-----S---DR------DV--
gi|304320687|re    -S--LFE-----------TNAPSRT-VAAFNYRYAFVSTGV--D-----D---SG------VA--
gi|154508096|re    -------P---AES--AEPALFAT-ADNWNREKYFPTVYT--A-----TSP-EGT--L--GV--
gi|172058831|re    -T--HVPD--GV---EDLPILNA-INDFNQEFKYFRVVL--D-----S---DR------DV--
gi|85705365|ref    -K--A-T----D---LTPEDLNR-ILQANFDTALDARYA--I-----A---KD------LL--
gi|119512017|re    LN-LP-----AK---EELKLMRH-LLEMNCSNTLEARFG--I-----I---EN------RV--
gi|256397448|re    -G-VVLED--VP---LSKKLLTA-VNEINSGYLWVRCYW--------A---QG------RL--
gi|94985189|ref    -K---QY----AD---RRQEVMAL-LNDRNRERAFSRS-I-D------A-DG--------NV--
gi|238927565|re    -VRLDEK----S---DPTVFHEK-LNEWNSEYRYVKFVV--D-----D---EQ------DV--
gi|304320540|re    -A--NFD-----LGRAVTDADFRV-VNGFNDSSVHGRAYV--L-----EN---RR------QI--
gi|163846985|re    -A--NPD----R---DLADRILY-VNRINDELHIVRSYV-D------R-SG--------DI--
gi|121606820|re    -FRKS------S---TSLQRLEL-ANEFNRETYMSSAFV-------K---DD------IL--
gi|121583407|re    -FKKS------T---SHLQRLEL-CNELNAKNYLVTSCV-------K---DD------HL--
gi|167856646|re    ----YFD----G---D-NTSLKS-VNKWNATKRFLTAYI-------DK---DGD------L--
gi|283780877|re    -Y--FVE--------EHNATLKK-VNDWNRNHYITKACI--L-----S---DT------SV--
gi|85708893|ref    -D--V-N----VD---NAARLDAF-INAFNDRQPTAKVMR--D-----P---QG------MV--
gi|304321485|re    -A--FSP----------MAGTTAV-MTDFNRTTPVARVTP--G-----P---DG------GA--
gi|304321021|re    -V--LMN----L----PASSPAL-VNNFNIERRTVSAGF--L-----E---GS------LT--

cons
```

```
E.amylovora-Amy    KLIVCQSLS--AAAG---LTYGQFV--------------------------------------
D.radiodurans-D    --WLEYVGFYPTL--A-EMPQETFD--------------------------------------
S.elongatus-T11    --QVVASRT--L---G-GITAGEIS--------------------------------------
E.coli-YbjN        KLVVCQSLS--VMQG---VTYEQFA--------------------------------------
gi|123253281|sp    --VLEMDVD--LD--FAGIPRENVG--------------------------------------
gi|123129053|sp    --GITRYVI--LD--R-GATIGNIK--------------------------------------
gi|123323499|sp    --VITRYVI--LD--G-GMTMRNVS--------------------------------------
gi|122544362|sp    --VVQAYMI--AD--G-GISMENYR--------------------------------------
gi|123736226|sp    --TMTWDVV--TG--K-GIDLEVFS--------------------------------------
gi|122459425|sp    --VLEMDVN--LR---HGVTRGNLE--------------------------------------
gi|122351524|sp    --VIEMNLP--AE--G-GFVREQLD--------------------------------------
gi|123004239|sp    --ALSLDLL--LA--G-GVSRDHLR--------------------------------------
gi|122476043|sp    --VLSLDVC--VA--G-GVAPNHLR--------------------------------------
gi|122295500|sp    --VLCLDVS--MA--G-GVTQDYLR--------------------------------------
gi|122403190|sp    --VMEKDVV--VA--D-GVSEKHLL--------------------------------------
gi|122257709|sp    --VMEMDVI--VA--G-GVSLANLR--------------------------------------
gi|81415193|sp|    --VLEADLD--LA---GGVADGAIR--------------------------------------
gi|81596955|sp|    --LFDYSFY--LK--G-GLTKKNLV--------------------------------------
gi|123557011|sp    --AFDHDLF--IK--G-GISKKNLV--------------------------------------
gi|81709081|sp|    --ALHTMRT--L---D-SLDPAEIS--------------------------------------
gi|123506214|sp    --VLNHHRT--L---E-GLSAGEIS--------------------------------------
gi|123556836|sp    --TMVASRT--V---A-DLSAGEIS--------------------------------------
gi|81670584|sp|    --VALHQRT--V---A-DLSPSEIS--------------------------------------
gi|122663130|sp    --VVLTQRT--V---E-DLSPGEIS--------------------------------------
gi|81771933|sp|    --VVISTRT--L---A-ELSPGEVS--------------------------------------
gi|123351931|sp    --VVVATRT--V---A-ELSPGEIS
gi|81596544|sp|    --WGVFQHD--LA----SLTSGDLYQAIASLFDLAQRGLDPFFTALAETQLRQIVRAAKQQGQ
gi|123608251|sp    --WAVYQHN--SS----TLTQDDLSSAIARLISLYEAGLNDVFNRLIESRIRQIVQAAKQQGQ
gi|81708722|sp|    --WGMYQHR--LS----TIGSEDLLAAVEQLQQLHDRGFQQAFEDLADAKLRQIVVALQDQAK
gi|298346581|re    --HTELPIS--WR--N-GLTDAQLD--------------------------------------
gi|260576430|re    --FIEMDFN--LD--FGGISDENFR--------------------------------------
gi|296129402|re    --VAEVATD--LE--H-GATDAQLS--------------------------------------
gi|254464399|re    --RIEMDVF--LG--ADGMSPDDFA--------------------------------------
gi|269795609|re    --YAEVSVD--LE--H-GATDEQLD--------------------------------------
gi|167648377|re    --YVQYDIN--VN--A-GRTVSGLD--------------------------------------
gi|302865994|re    --CGEVTTD--LE--R-GVTPHQLD--------------------------------------
gi|254474961|re    --RIEFDIY--LG--NDGMDPDDFA--------------------------------------
gi|238063407|re    --CGEVIAD--LE--R-GVTPHQLD--------------------------------------
gi|227495918|re    --MAEHNVD--FE--L-GATDEQIA--------------------------------------
gi|269219692|re    --MTEHSVD--YE--H-GVTDAQIS--------------------------------------
gi|145593940|re    --CGEVITD--LE--R-GVTPHQLD--------------------------------------
gi|269976471|re    --HTELPIS--YH--A-GLTDSQLD--------------------------------------
gi|217969472|re    --NLEMEVN--LD---YGVSAKNIE--------------------------------------
gi|399991304|re    --RVELDVY--LG--DTGMNPDDFA--------------------------------------
gi|256832278|re    --YGESNMD--FE--H-GATYDQVG--------------------------------------
gi|225022571|re    --RTELNID--ME--Y-GASDLQLK--------------------------------------
gi|126738030|re    --RIEMDIF--LG--RDGVSADDFA--------------------------------------
gi|254294753|re    --RLQMDVN--LD--G-GVTAGNIE--------------------------------------
gi|149184640|re    --AIEMDID--LE-AG-GMSKELFI--------------------------------------
gi|257068239|re    --YGELAAD--FR---AGALDSQID--------------------------------------
gi|229820926|re    --TTEVSVP--IQ--H-GLNDVQLE--------------------------------------
gi|227494902|re    --HTQLTYD--WE--H-GVSDSQLE--------------------------------------
gi|220934858|re    --IFESDLD--LD--G-GITVDRLK--------------------------------------
gi|291295614|re    --TLDWHID--LE---MGISPALFA--------------------------------------
gi|296130820|re    --YAEHTVD--YE--H-GVADAQVD--------------------------------------
gi|84685465|ref    --RIEMDVA--TS--NDGLSTGDFD--------------------------------------
gi|229820927|re    --VAETNLG--VR--F-GASDEQLT--------------------------------------
gi|227496193|re    --GASHMVD--WE--H-GVADCQLE--------------------------------------
gi|154507942|re    --VAEVNAD--YE--Y-GATDAQLV--------------------------------------
gi|86137245|ref    --KLEMDLY--MG--KDGISADDFA--------------------------------------
gi|227494903|re    --CCEHSID--LE--H-GVTDKQLA--------------------------------------
gi|152982702|re    --VLEMDVM--VA--G-GVSPAHLA--------------------------------------
gi|119486978|re    --IVLSNRT--L---A-GITPAEIS--------------------------------------
gi|145596051|re    --YAELQVP--FA---AGVHDALLV--------------------------------------
gi|220903645|re    --TMEMDVF--MR---YGMTQKNLE--------------------------------------
gi|297566041|re    --VLEFDLW--LE----GASPELIM--------------------------------------
gi|227496194|re    --FACHAVD--YE--Y-GLSDLQLA--------------------------------------
gi|119385461|re    --FIEMDIG--VA--GDGIGRKNFD--------------------------------------
gi|183220878|re    --VLESDFD--YS---GGVSEEAIK--------------------------------------
gi|291302096|re    --YGETQVP--LR---PGVPDDLLD--------------------------------------
gi|387905553|re    --ALEMDVV--VA--G-GVSERHLR--------------------------------------
gi|238927566|re    --VVDIDLP--LDLHKGEFKPNAVI--------------------------------------
gi|322420651|re    --SIQIVVP--FH---DNFSSEVIV--------------------------------------
gi|225629038|re    --FLEMDVV--LE--G-GVTQTYLQ--------------------------------------
gi|227495837|re    --HGEVTYP--LG--A-GMTDAQLE--------------------------------------
gi|291295390|re    --VLESDLR--LE----GVTPEAIH--------------------------------------
gi|302381558|re    --VAQYDIL--LV--P-AVGPSQLD--------------------------------------
gi|229589918|re    --LMEMDVV--VA--G--VGATHLR--------------------------------------
gi|229917768|re    --TLASTLD--LD--L-GFSPEIIL--------------------------------------
gi|304320687|re    --YVSRYDI--AD--Y-GTPKGNIA--------------------------------------
gi|154508096|re    --YADFVVD--TE--A-GLSDVQLR--------------------------------------
gi|172058831|re    --TIASTID--LD--H-GFNPAAIF--------------------------------------
gi|85705365|ref    --WAAFIHP--LK----PLEKDEFI--------------------------------------
gi|119512017|re    --VVISTRT--L---E-DLSPAEVS--------------------------------------
gi|256397448|re    --TVARDLA--AD----TLTADQLT--------------------------------------
gi|94985189|ref    --WLEYVGFYPTL--A-EMPQETFD--------------------------------------
gi|238927565|re    --VVDIDLP--LDLHDGVFQVDSFM--------------------------------------
gi|304320540|re    --GVDFTID--MV--G-GVTSDHVD--------------------------------------
gi|163846985|re    --GFDGYLV--VS--G-GVTRRNII--------------------------------------
gi|121606820|re    --KITHVIS--YRDG---LLTETLI--------------------------------------
gi|121583407|re    --LFDYILN--YRDG---LLRETFI--------------------------------------
gi|167856646|re    --ALKDDLD--VE---EGISEAYLL--------------------------------------
gi|283780877|re    --SMGADLV--VA----GSKKTTMA--------------------------------------
gi|85708893|ref    --ALQAYIN--AA--N-GITYRNLL--------------------------------------
gi|304321485|re    --MLYHYVI--GD--H-GVTQGSLM--------------------------------------
gi|304321021|re    --GLTYRQV--LR--G-GVLAENVG--------------------------------------

cons
```

```
E.amylovora-Amy     -----------------------------HFMKESEEQISMIVMEAFANHLLMIAEDEERPPM
D.radiodurans-D     -----------------------------TLFGGVLMHFQDDYAALEG-YV----PQEGMQI-
S.elongatus-T11     -----------------------------RLITIVATLADDYDDALRA-EF----K-G-----
E.coli-YbjN         -----------------------------WFVRQSEEQISMVILEANAHQLLLPTDDEGQNN-
gi|123253281|sp     -----------------------------ETFNTWASLMDSFRDHVFE---------------
gi|123129053|sp     -----------------------------DNLLNVLAIAPNAANYIWQ-AG----EYAPGY--
gi|123323499|sp     -----------------------------DNLTNALAIATSVADYVWE-VG----DYAPGA--
gi|122544362|sp     -----------------------------MQLVVYSQMLKKMRETIYK-DG------------
gi|123736226|sp     -----------------------------NAVDMFRSAMDTLGTEVF----------------
gi|122459425|sp     -----------------------------DTFDWWRLSLREFKSGVLK---------------
gi|122351524|sp     -----------------------------YIFSQWTIALAEFSRDVIA-EA----P-------
gi|123004239|sp     -----------------------------AEIEIWDHLVQQLIVFLRE-EL----AGLAQP--
gi|122476043|sp     -----------------------------AQIEIWDHLVQQLITYLRE-EL----PKLAPV--
gi|122295500|sp     -----------------------------GQIEIWDQLVQQLIGYLRE-EL----KQLSVS--
gi|122403190|sp     -----------------------------GSLLLWDRLLQEFIVYLRD-YS----RNVAEQ--
gi|122257709|sp     -----------------------------SHIELWDRLLQEFIAYLRD-YS----RLAAEQ--
gi|81415193|sp|     -----------------------------AFLDLFEENLRAFAAWIGW---------------
gi|81596955|sp|     -----------------------------LGTRFFQSIPLEAIHEHGK-EM----VV------
gi|123557011|sp     -----------------------------LATRFFLSIPLDAINDCGE-EL----VA------
gi|81709081|sp|     -----------------------------RAITIVATLADEYDEPLIE-QF----GGTAPF--
gi|123506214|sp     -----------------------------RAITLVASLADEYDEQLQA-EF----PKA-----
gi|123556836|sp     -----------------------------RAITLVASIAATYAEGLP---------S------
gi|81670584|sp|     -----------------------------RAITLVATLADDHDDRLKE-KY----GA------
gi|122663130|sp     -----------------------------LAITLVATIADDNDEKLIE-TY----GCNSVT--
gi|81771933|sp|     -----------------------------RIITIVATIADDNDEALQS-EF----GA------
gi|123351931|sp     -----------------------------RAITIVATLADDNDELLVA-EF----G-------
gi|81596544|sp|     SLPATLQTLTHLYEEGVLGDLSNGPEIRRFTLDRWREQLERLWPEVEV-D-------------
gi|123608251|sp     SLEGTMQNLERFYAEGLLGEINQTVDSREEVLAAWKRQLERLWNEVN----------------
gi|81708722|sp|     TLEEALQLLERLYDEGVLGTLQSTREDRELTLQVWRLKLTRLWHEADS-P-------------
gi|298346581|re     -----------------------------EQVRCALESSEDFFEQLEQ-QL----PNAMPQP-
gi|260576430|re     -----------------------------DSLDIWERLVGDFKKHIN----------------
gi|296129402|re     -----------------------------QILFCGLSTGSMLFDALDE-RY----PDPAGAA-
gi|254464399|re     -----------------------------QTVSLWTRAMQDFEEFIG---W------------
gi|269795609|re     -----------------------------RLLACGLSTSAMLFDTLDG-LY----PDPAAVA-
gi|167648377|re     -----------------------------DDFGVWTGMLGDFTKFID----------------
gi|302865994|re     -----------------------------RLVDCGVTTGCQLAAAVDE-LA----GGTL----
gi|254474961|re     -----------------------------ETLGIWSRIVQDFETLID---W------------
gi|238063407|re     -----------------------------QLLDCGISTGCQLAAAAGQ-LA----GGVR----
gi|227495918|re     -----------------------------LAVHCAMTTGLRFFDHLTT-EI----FPEEWKV-
gi|269219692|re     -----------------------------QHIACAISTGTQFFEYLNE-TF----PQEWEAY-
gi|145593940|re     -----------------------------RLLDRGITTGCQLAADVAR-LP----GGVR----
gi|269976471|re     -----------------------------EHLHCALESSEDFFEKLGE-KF----PQFDEGT-
gi|217969472|re     -----------------------------DSFNWWTKALKEYKKVVLE---------------
gi|399991304|re     -----------------------------ELLGYWSRIVQEFEEFIN---W------------
gi|256832278|re     -----------------------------TTLACAIQTTTQFFDELDR-HI----PDPALHR-
gi|225022571|re     -----------------------------QSLDCGIATSMSFFESLDK-KF----PDTRFA--
gi|126738030|re     -----------------------------SMVSLWARSMSEFEDMID---W------------
gi|254294753|re     -----------------------------DTLDIWRRLLETYETEFAA-QA----PA------
gi|149184640|re     -----------------------------DNLLYWESVMVGFAEFSFS-KD----N-------
gi|257068239|re     -----------------------------SAITCGLSTVIAFFHSLEE-RL----GAEIDE--
gi|229820926|re     -----------------------------HHVQLGLMSGMMVFDHLDE-MF----PDPVALQ-
gi|227494902|re     -----------------------------MQLQCAVTTSRQFFEELEA-AL----D-------
gi|220934858|re     -----------------------------DWLQTFNMLIDMFVREVVH-GQ----GVDDSK--
gi|291295614|re     -----------------------------DMCNTVMLAAHQFVMELNP-GR----GQGVAE--
gi|296130820|re     -----------------------------LQLACGISTALQLFEHLDE-QY----PAEAAAA-
gi|84685465|ref     -----------------------------ALLELWLDSVVLFEDHIK---W------------
gi|229820927|re     -----------------------------ETITLALSVTAQFFAGLEA-SV----PPAREE--
gi|227496193|re     -----------------------------QQVACAMGTIADAFADVRS-RL----ALRADNL-
gi|154507942|re     -----------------------------QQVMCALATTLQLFRALEE-RY----GLDDDEG-
gi|86137245|ref     -----------------------------TTVGLWSRLMGDFEELID---Y------------
gi|227494903|re     -----------------------------LQLHCAIATSLDAIRTGYA-EL----GISLEEE-
gi|152982702|re     -----------------------------VSLQLWTQMMGQFFLHLRN-FK----PESVPA--
gi|119486978|re     -----------------------------RIITVVATIADDNDDDLQA-EF----G-------
gi|145596051|re     -----------------------------EMLDCGAAVATSFVDWLHD-EG----AL------
gi|220903645|re     -----------------------------EIFDLWKTSLRHLSEVIAA-QS----KKNPS---
gi|297566041|re     -----------------------------TFVREFEDSCNLFFSYIRM-ID----AELV----
gi|227496194|re     -----------------------------QHVDCAIATVNGLFADLNE-VL----G-------
gi|119385461|re     -----------------------------DALDTWRIVLSDFRDFID----------------
gi|183220878|re     -----------------------------EFLQKFQILNSQFSTLLIL-AE------------
gi|291302096|re     -----------------------------EFVDCAAAVSGAYIDWLHG-EA----GI------
gi|387905553|re     -----------------------------STVELWDRLIQEFLLHLRD-RP----TLAEQE--
gi|238927566|re     -----------------------------AMMAVGMRAVEQVHESLIG-LC----ERGHRP--
gi|322420651|re     -----------------------------DMVALIFRAMKEEHPRLLK-VV------------
gi|225629038|re     -----------------------------YVLAIWADLIQAFLVHLRD-DQ----NILLRY--
gi|227495837|re     -----------------------------SFVFTSCRLIVALMREAEN-LF----PDPLRGN-
gi|291295390|re     -----------------------------AFVHDFGDQVTLFFSYLRM-ID----AGLV----
gi|302381558|re     -----------------------------DPIQVWRSLSNDLGRTVTV-AA----NAAPAV--
gi|229589918|re     -----------------------------SQLELWDRLLQEFIVYLRE-YS----QQTAQL--
gi|229917768|re     -----------------------------QHSFMMFQAADEVNEYMQK-LY----TQV-----
gi|304320687|re     -----------------------------VSIANYLHMASTFDRHLFE-AT----QTVQKE--
gi|154508096|re     -----------------------------DAISSGISTGIAAIQYVKE-SA----SEALGL--
gi|172058831|re     -----------------------------GHSVMMFQASDEVFAYLTK-LY----DQV-----
gi|85705365|ref     -----------------------------SGLGQVVNLAQSYGTLYSG-GA----LHYGGG--
gi|119512017|re     -----------------------------RLVTIVATIADENDEVLQA-EF----GL------
gi|256397448|re     -----------------------------GACAQIGRIADEYDDRFKE-LV----GAGHTVF-
gi|94985189|ref     -----------------------------TLFGGVLMHFQDDYAALEG-Y------VPGPQL-
gi|238927565|re     -----------------------------SMVGVGLQVLEEVYPALMK-LR----W-------
gi|304320540|re     -----------------------------ERLKRWPELIRTFRDQMRN-AQ----TG------
gi|163846985|re     -----------------------------FATRTFIDHVRAALAKDET-DV----IA------
gi|121606820|re     -----------------------------RACRQFSGGISLAIDEFDP-EY----KILMRL--
gi|121583407|re     -----------------------------RSCRQFARNLEKGLELVDP-EN----DFVLPP--
gi|167856646|re     -----------------------------DTLTRIFVGAYLFASEMKK-E-------------
gi|283780877|re     -----------------------------KFLNYYFSQLAPFYKEVCE---------------
gi|85708893|ref     -----------------------------AQMVVFGQNITTLSRALIE-LE----N-------
gi|304321485|re     -----------------------------VNLRAFASSTQKWESVAGR-GS----SRVVSF--
gi|304321021|re     -----------------------------FTFGLFLEDANTIQEMVIE-QS----GPGPFE--

cons
```

```
E.amylovora-Amy   ITSHSLL-------H------------------------------------------------
D.radiodurans-D   ---QQP-------Q--------A----------------------------------------
S.elongatus-T11   ---------------------------------------------------------------
E.coli-YbjN       VTENYFL-------H------------------------------------------------
gi|123253281|sp   -----------------E---------------------------------------------
gi|123129053|sp   E--AEYEDDEDYFYED-----------------------------------------------
gi|123323499|sp   E--DEYDGD------------------------------------------------------
gi|122544362|sp   ---------------------------------------------------------------
gi|123736226|sp   ---------------------------------------------------------------
gi|122459425|sp   -----------------L---------------------------------------------
gi|122351524|sp   ---------------------------------------------------------------
gi|123004239|sp   T--NANGGGAAASPAVEPGPSPAATM-------------------------------------
gi|122476043|sp   N--TPSPGAALNHQPKVASPGDDAGESASS-VSA-----------------------------
gi|122295500|sp   S--DAAARPENQAPQSQEPSRDRTAQLAAS-ALI-----------------------------
gi|122403190|sp   T--GSEAVSA-----------------------------------------------------
gi|122257709|sp   Q--GMAQDAGQPVDGE-----------------------------------------------
gi|81415193|sp|   -----------------E---------------------------------------------
gi|81596955|sp|   ---------------------------------------------------------------
gi|123557011|sp   ---------------------------------------------------------------
gi|81709081|sp|   P--------------------------------------------------------------
gi|123506214|sp   ---------------------------------------------------------------
gi|123556836|sp   ---------------------------------------------------------------
gi|81670584|sp|   ---------------------------------------------------------------
gi|122663130|sp   ---------------------------------------------------------------
gi|81771933|sp|   ---------------------------------------------------------------
gi|123351931|sp   ---------------------------------------------------------------
gi|81596544|sp|   ---------------SW-EQ-------------------------------------------
gi|123608251|sp   ---------------------------------------------------------------
gi|81708722|sp|   ---------------Q-----------------------------------------------
gi|298346581|re   ---DEDS--------------------------------------------------------
gi|260576430|re   -----------------F---------------------------------------------
gi|296129402|re   ---------------------------------------------------------------
gi|254464399|re   ---------------------------------------------------------------
gi|269795609|re   ---------------------------------------------------------------
gi|167648377|re   ---------------------------------------------------------------
gi|302865994|re   ---------------------------------------------------------------
gi|254474961|re   ---------------------------------------------------------------
gi|238063407|re   ---------------------------------------------------------------
gi|227495918|re   ---FEAEQAEREALG------------------------------------------------
gi|269219692|re   ---QVQAEAQAIAVAEAQAVAEAEADARYN-FKGD--------SDRPEEDLTRPDSLALPEPP
gi|145593940|re   ---------------------------------------------------------------
gi|269976471|re   ---PDEE--------------------------------------------------------
gi|217969472|re   -----------------E---------------------------------------------
gi|399991304|re   ---------------------------------------------------------------
gi|256832278|re   ---------------------------------------------------------------
gi|225022571|re   ----------------------Q----------------------------------------
gi|126738030|re   ---------------------------------------------------------------
gi|254294753|re   ---------------------------------------------------------------
gi|149184640|re   ---------------------------------------------------------------
gi|257068239|re   L---------DS---------------------------------------------------
gi|229820926|re   ---PD-G--------------------------------------------------------
gi|227494902|re   ---------------------------------------------------------------
gi|220934858|re   P--VTPRRDRELRAGISPTV-------------------------------------------
gi|291295614|re   M--QDLFRTLLERLRERR---------------------------------------------
gi|296130820|re   ---KAAQEQ------------------------------------------------------
gi|84685465|ref   ---------------------------------------------------------------
gi|229820927|re   ---------------------------------------------------------------
gi|227496193|re   ---NESQ--------------------------------------------------------
gi|154507942|re   ---HGPAGGHQRGFDGPAWLPES----------------------------------------
gi|86137245|ref   ---------------------------------------------------------------
gi|227494903|re   ---E-----------------------------------------------------------
gi|152982702|re   T--DAAAAVTADESVA-----------------------------------------------
gi|119486978|re   ---------------------------------------------------------------
gi|145596051|re   -----------L---------------------------------------------------
gi|220903645|re   ---------------------------------------------------------------
gi|297566041|re   --------------E------------------------------------------------
gi|227496194|re   ---------------------------------------------------------------
gi|119385461|re   -----------------W---------------------------------------------
gi|183220878|re   ---------------------------------------------------------------
gi|291302096|re   -----------L---------------------------------------------------
gi|387905553|re   A--AARAADADRPDAPAAVTASSGAAVP-----------------------------------
gi|238927566|re   R--ARAS-------E------------------------------------------------
gi|322420651|re   -----------Q---------------------------------------------------
gi|225629038|re   K--RRKQAEQRIQILATCTSGKLRIQPI-----------------------------------
gi|227495837|re   ---LE----------------------------------------------------------
gi|291295390|re   --------------E------------------------------------------------
gi|302381558|re   P--PA----------------------------------------------------------
gi|229589918|re   Q--AAAVGEEAPV--------------------------------------------------
gi|229917768|re   --------------Q------------------------------------------------
gi|304320687|re   A--LDTDLAANGLNMQGILADGDLAAQIGL-SPSS--------HQVSFEAMTDVVDTFIKADG
gi|154508096|re   G--ESGR--------------E-----------------------------------------
gi|172058831|re   --------------Q------------------------------------------------
gi|85705365|ref   D--SGALQRALIDELLKKGEGI-----------------------------------------
gi|119512017|re   ---------------------------------------------------------------
gi|256397448|re   ---DGDPGDDDSVEV------------------------------------------------
gi|94985189|ref   ---QQP-------Q--------A----------------------------------------
gi|238927565|re   --------------T------------------------------------------------
gi|304320540|re   ---------------------------------------------------------------
gi|163846985|re   ---------------------------------------------------------------
gi|121606820|re   M--KTEPESESSENE------------------------------------------------
gi|121583407|re   G--KIEPEDEETP--------------------------------------------------
gi|167856646|re   ---------------------------------------------------------------
gi|283780877|re   ---------------------------------------------------------------
gi|85708893|ref   ---------------------G-----------------------------------------
gi|304321485|re   T--PLVPDGPP---VSEGKEDTHLKEATAIAAPADPADPHLVTLSPGQTSSDSARGNALMIKQ
gi|304321021|re   V--SVPPDERFPRLASAVAATGWDVSRLVA-RFGP--------LSPERASPQVPFPSITGFGL

cons
```

```
E.amylovora-Amy    --------------------
D.radiodurans-D    --------------------
S.elongatus-T11    --------------------
E.coli-YbjN        --------------------
gi|123253281|sp    --------------------
gi|123129053|sp    -------------------W
gi|123323499|sp    -------------------W
gi|122544362|sp    --------------------
gi|123736226|sp    -------------------K
gi|122459425|sp    --------------------
gi|122351524|sp    --------------------
gi|123004239|sp    -------------------Q
gi|122476043|sp    -------------------A
gi|122295500|sp    -------------------Q
gi|122403190|sp    -------------------S
gi|122257709|sp    -------------------A
gi|81415193|sp|    --------------------
gi|81596955|sp|    --------------------
gi|123557011|sp    --------------------
gi|81709081|sp|    -------------------A
gi|123506214|sp    --------------------
gi|123556836|sp    --------------------
gi|81670584|sp|    --------------------
gi|122663130|sp    -------------------K
gi|81771933|sp|    -------------------A
gi|123351931|sp    -------------------Q
gi|81596544|sp|    -------------------S
gi|123608251|sp    -------------------S
gi|81708722|sp|    -------------------N
gi|298346581|re    -------------------E
gi|260576430|re    --------------------
gi|296129402|re    -------------------P
gi|254464399|re    --------------------
gi|269795609|re    -------------------P
gi|167648377|re    -------------------W
gi|302865994|re    -------------------R
gi|254474961|re    --------------------
gi|238063407|re    -------------------P
gi|227495918|re    -------------------E
gi|269219692|re    FHDSSYQPQAEEDDSRKSED
gi|145593940|re    -------------------S
gi|269976471|re    -------------------A
gi|217969472|re    --------------------
gi|399991304|re    --------------------
gi|256832278|re    -------------------P
gi|225022571|re    --------------------
gi|126738030|re    --------------------
gi|254294753|re    -------------------D
gi|149184640|re    -------------------D
gi|257068239|re    --------------------
gi|229820926|re    -------------------A
gi|227494902|re    -------------------A
gi|220934858|re    -------------------G
gi|291295614|re    --------------------
gi|296130820|re    -------------------D
gi|84685465|ref    --------------------
gi|229820927|re    -------------------S
gi|227496193|re    -------------------W
gi|154507942|re    --------------------
gi|86137245|ref    --------------------
gi|227494903|re    -------------------A
gi|152982702|re    -------------------A
gi|119486978|re    -------------------Q
gi|145596051|re    --------------------
gi|220903645|re    --------------------
gi|297566041|re    --------------------
gi|227496194|re    -------------------R
gi|119385461|re    --------------------
gi|183220878|re    --------------------
gi|291302096|re    --------------------
gi|387905553|re    -------------------S
gi|238927566|re    --------------------
gi|322420651|re    --------------------
gi|225629038|re    -------------------W
gi|227495837|re    -------------------P
gi|291295390|re    --------------------
gi|302381558|re    -------------------Q
gi|229589918|re    -------------------L
gi|229917768|re    --------------------
gi|304320687|re    LAPGRIVN------AVTGKR
gi|154508096|re    --------------------
gi|172058831|re    --------------------
gi|85705365|ref    --------------------
gi|119512017|re    -------------------A
gi|256397448|re    --------------------
gi|94985189|ref    --------------------
gi|238927565|re    --------------------
gi|304320540|re    -------------------S
gi|163846985|re    --------------------
gi|121606820|re    --------------------
gi|121583407|re    --------------------
gi|167856646|re    --------------------
gi|283780877|re    -------------------E
gi|85708893|ref    --------------------
gi|304321485|re    IAFEPTLS------PKE--R
gi|304321021|re    AVQE---------------D

cons
```
