# Supplementary material for: The crystal structure of Erwinia amylovora AmyR, a member of the YbjN protein family, shows similarity to type III secretion chaperones but suggests different cellular functions
Source: PLoS One. 2017 Apr 20;12(4):e0176049. doi: 10.1371/journal.pone.0176049 (PMC5398634; doi:10.1371/journal.pone.0176049)
Supplement: S3 Fig — (PDF) [file pone.0176049.s003.pdf]

```
                                                                                                                  1
E.amylovora-AmyR                      .......................................................................M.H............
D.radiodurans-DR1245                  ME....................................................................................
S.elongatus-T110839                   M.......................................................T..MVSE.VQP.......VSP.......AS
E.coli-YbjN                           ......................................................................................
gi|123253281|sp|Q1GTJ6|Q1GTJ6_SPHAL   ................................................MGPITMRA..AL.........TL...AA..TALLWASP
gi|123129053|sp|Q0C3X0|Q0C3X0_HYPNA   M.......................................................TVLTLLG.A..............AVLPAHA
gi|123323499|sp|Q0C3W9|Q0C3W9_HYPNA   ......................................................................................
gi|122544362|sp|Q2NA07|Q2NA07_9SPHN   ...........................................MRHLILAATIAAG..TA.........TM...AF..TPATAQSF
gi|123736226|sp|Q2G8G7|Q2G8G7_NOVAD   M.N...................................V..............RWL..LA.........AL...AA..GVMAPSVA
gi|122459425|sp|Q1YFZ1|Q1YFZ1_9RHIZ   ..........................................MWCVPLSGGYPVRF..IA.........TL...LA..CLCLAGAS
gi|122351524|sp|Q0G7X7|Q0G7X7_9RHIZ   M.P..............................A.S......SQSFVFALLFLFAG..VP.........QA...LA..VGEAAESP
gi|123004239|sp|Q2IXX8|Q2IXX8_RHOP2   ......................................................................................
gi|122476043|sp|Q213H6|Q213H6_RHOPB   ......................................................................................
gi|122295500|sp|Q07KZ5|Q07KZ5_RHOPA   ....................................................................................MI
gi|122403190|sp|Q1I9R8|Q1I9R8_9PSED   .....................................................................................M
gi|122257709|sp|Q02HI9|Q02HI9_PSEAE   .....................................................................................M
gi|81415193|sp|Q745S9|Q745S9_THET2    ................................................M.........A..........RL...FL..LALILVPA
gi|81596955|sp|Q5N656|Q5N656_SYNP6    ..................................................................................MMLT
gi|123557011|sp|Q31PB3|Q31PB3_SYNP7   ..................................................................................MMLT
gi|81709081|sp|Q7NG79|Q7NG79_GLOVI    M.P.....................PRRHADSASILVEYPGWAKTADDTCPTGQGEA..HRMT.T...............HEEALSN
gi|123506214|sp|Q2JU30|Q2JU30_SYNJA   M.......................................................V..Y.....SE.......PLIDRSPEGFPG
gi|123556836|sp|Q31NC7|Q31NC7_SYNP7   M.......................................................A..VSSE.T..............TLDSSAL
gi|81670584|sp|P73244|P73244_SYNY3    M.......................................................T..MASP.T.............PELANPTM
gi|122663130|sp|Q4C8F6|Q4C8F6_CROWT   M.......................................................T..NSTT.E...............YTLPDN
gi|81771933|sp|Q8YVL5|Q8YVL5_ANASP    M.......................................................T..SYQE.TLTN..........NECIDELI
gi|123351931|sp|Q10XZ9|Q10XZ9_TRIEI   M.......................................................T..TNNP.ETVSVDN...ASIEEANINELT
gi|81596544|sp|Q5N4K3|Q5N4K3_SYNP6    ........................................................M.............................
gi|123608251|sp|Q3M567|Q3M567_ANAVT   ........................................................M.............................
gi|81708722|sp|Q7NEZ9|Q7NEZ9_GLOVI    ......................................................................................
gi|298346581|ref|YP_003719268.1|      M.................................................SGDSGN..LF.........DN...PF..ESPFDSVP
gi|260576430|ref|ZP_05844420.1|       .....................................................MKL..QL.........FS...LV..LAATLAGP
gi|296129402|ref|YP_003636652.1|      MRWFRQ...LVRRWTGARPGAQTPPQ...................PLDDIELHDRV..AE.........LL...VR..ELGSAE..
gi|254464399|ref|ZP_05077810.1|       M.E.............................T..................KMSIV..KA.........IA...AC..VALVLPAA
gi|269795609|ref|YP_003315064.1|      MGLFKD...RRHRRTDDHSGVRASRRGTAGGDDAGGRAADG..SAGQVSDEQLRIDV..EQ.........VL...AR..ELGALVDS
gi|167648377|ref|YP_001686040.1|      M.K................................................TTLMA..LA.........AA...MA..LAAGGAAH
gi|302865994|ref|YP_003834631.1|      M.........................................................AS.........PE...IE..DGPDGPLA
gi|254474961|ref|ZP_05088347.1|       .....................................................................MT...AA..ALFALPGF
gi|238063407|ref|ZP_04608116.1|       M.........................................................AS.........PE...IE..DGPDGPLA
gi|227495918|ref|ZP_03926229.1|       M..........................................................................G..FFSKDAST
gi|269219692|ref|ZP_06163546.1|       M................................................................................AFVDN
gi|145593940|ref|YP_001158237.1|      M.........................................................AS.........PT....G..DIPDRLLG
gi|269976471|ref|ZP_06183456.1|       M..........................................................................A..DYEEYFQS
gi|217969472|ref|YP_002354706.1|      ................................................MKGLSKGS..LF.........FC...AL..LSAAAGPA
gi|399991304|ref|YP_006571544.1|      ....................................................MKFL..SL.........LT...AA..ALFVLPGL
gi|256832278|ref|YP_003161005.1|      MGTMNW.....................................RTLRSRITGGTRA..ET.........TV...TP..GRVHHQRH
gi|225022571|ref|ZP_03711763.1|       MVNHNK.......T.......................L....EGLHMTDQNDQSEN..NP.........SI...SG..RVERIIPT
gi|126738030|ref|ZP_01753751.1|       .....................................................................ML...AA..AIALSPVA
gi|254294753|ref|YP_003060776.1|      M............................SFSRFDFPAAI..SACLIIIAGIIASS..QT.........SF...AQ..NTPASAER
gi|149184640|ref|ZP_01862958.1|       M...................................................IKHA..LS.........GA...VI..AAGLLAAS
gi|257068239|ref|YP_003154494.1|      ...................................................................................MGK
gi|229820926|ref|YP_002882452.1|      ......................................................................................
gi|227494902|ref|ZP_03925218.1|       ....................................................................................MA
gi|220934858|ref|YP_002513757.1|      .......................................................MR..HLLPLVTA..LV...LS..LLLVLPAQ
gi|291295614|ref|YP_003507012.1|      ...................................................................................MEM
gi|296130820|ref|YP_003638070.1|      M....................................................................GF...FT..KDTSVPLQ
gi|84685465|ref|ZP_01013363.1|        M.S.............................RKVIAAVL..AILAAPALAQEQKS..LE.........SK...PL..DAPASVQA
gi|229820927|ref|YP_002882453.1|      M.......................................................A.......G..............TQRGSAA
gi|227496193|ref|ZP_03926499.1|       MR.................................................GVRGL..FS.........RA...RR..RDSGAASA
gi|154507942|ref|ZP_02043584.1|       ......................................................................................
gi|86137245|ref|ZP_01055823.1|        ....................................................MKLT..KL.........FA...VA..ALAISPVA
gi|227494903|ref|ZP_03925219.1|       MGMFSK....................................LFGADDEGAQQIPN..LP.........EE...AV..EEPRFQPA
gi|152982702|ref|YP_001354063.1|      ......................................................MS..KS.........TS...AA..AAAVAETT
gi|119486978|ref|ZP_01620850.1|       M.......................................................S..STTP.Q..N..........LEAISEDL
gi|145596051|ref|YP_001160348.1|      MPWWSWRPGPADGGEPGSRSRVT...................VEEAVRVSPPAPRQ..PG.........DN...YA..GVDRPPAV
gi|220903645|ref|YP_002478957.1|      ................................................MKKLLTLV..LS.........LC...LT..CIFAAGAM
gi|297566041|ref|YP_003685013.1|      M.....................................................................................
gi|227496194|ref|ZP_03926500.1|       MREHHL......I.......................STSA..LAPTSHRDPTLSLL..SR.........FS...RP..DPTPRAGA
gi|119385461|ref|YP_916517.1|         .....................................................MR...T..........LV...PL..VLAALMQA
gi|183220878|ref|YP_001838874.1|      .......................................................M..SA.........QS...PK..GKESESAS
gi|291302096|ref|YP_003513374.1|      MAWWRKRPETRASGYDDAKRREPQPAAIPAPREEMTTPSNP..DDRWDIIKQDLIAL..DP.........DF...FN..DLEQLTDE
gi|387905553|ref|YP_006335891.1|      ..........................................................M..........EH...EQ..TNTADERA
gi|238927566|ref|ZP_04659326.1|       .......................................................................M.EGT..MGLFDGFQ
gi|322420651|ref|YP_004199874.1|      ......................................................................................
gi|225629038|ref|ZP_03787072.1|       ......................................................................M...II..PRSLAPAS
gi|227495837|ref|ZP_03926148.1|       ......................................................................M...SS..STPTTSQV
gi|291295390|ref|YP_003506788.1|      M.....................................................................................
gi|302381558|ref|YP_003817381.1|      .................................................MKLAVLL..AS.........CA...VI..ASPAGAQT
gi|229589918|ref|YP_002872037.1|      .....................................................................................M
gi|229917768|ref|YP_002886414.1|      M.....................................................................................
gi|304320687|ref|YP_003854330.1|      M.G.........................QMGDRRMVKTKF..SALALAALMPMTAA..AQ.........SN...AL..TAPDSLVS
gi|154508096|ref|ZP_02043738.1|       ..............................................................................MNVPYLVP
gi|172058831|ref|YP_001815291.1|      M.....................................................................................
gi|85705365|ref|ZP_01036464.1|        M.K.............................GS..............PMLRRLLA..LS.........LV...LL..ATPLTAQD
gi|119512017|ref|ZP_01631112.1|       M.......................................................T..NYQE.TLPS..............SEFN
gi|256397448|ref|YP_003119012.1|      M.....................................................................................
gi|94985189|ref|YP_604553.1|          MTM...................................................................................
gi|238927565|ref|ZP_04659325.1|       ..............................................................................MGLFDGFS
gi|304320540|ref|YP_003854183.1|      M.G.............................HR....H..............IMA..LF.........AG...LL..LCAGSAAA
gi|163846985|ref|YP_001635029.1|      M................................................................................TGAQP
gi|121606820|ref|YP_984149.1|         .....................................................MSA..PA.........LANKT.....KPENFAM
gi|121583407|ref|YP_973838.1|         .....................................................................................M
gi|167856646|ref|ZP_02479340.1|       M.K.............................I...YKYL..KTPAYFLSMFFAML..IT.........TT...LV..STTAYAET
gi|283780877|ref|YP_003371632.1|      M.F........................RPLLASLVVFATL..LTTSTLAAQDAPPL..GG.........LI...GN..AGNGGGAG
gi|85708893|ref|ZP_01039959.1|        M.L............................IRSIIS.....TSLAALALAVTSTP..AA.........AQ...QA..PSQTQAQS
gi|304321485|ref|YP_003855128.1|      M.E.........................G..AMM.........RKIAAMIGVGMLA..AA.........PA...LA..LTQLSTDI
gi|304321021|ref|YP_003854664.1|      M.R.............................AT....IG..VAGALLSLFLGGAS..TA.........QS...ND..LTVEENPL
consensus>70                          ......................................................................................
```

```
                                                                             10        20                      30
E.amylovora-AmyR                      ..M.V.S............................LVVPDLDVLRRWLDQQSIT.......WF....ECD...SC.QAL.H.L.P.HM
D.radiodurans-DR1245                  ......T.............................ALLTLDTLAKYLQEKEVQ.......LD....IEENGG....QR.....F.IR
S.elongatus-T110839                   L...D.A............................PLENAVEIIETVISSLHQGDA...PLVG....QT.D..S....G...K..IWM
E.coli-YbjN                           ..M.T.S............................LVVPGLDTLRQWLDDLGMS.......FF....ECD...NC.QAL.H.L.P.HM
gi|123253281|sp|Q1GTJ6|Q1GTJ6_SPHAL   AHA.E.L............................VNAANPATIKAIVESQGWP.......AT....IVAK....EG.D.D.P.Y.IE
gi|123129053|sp|Q0C3X0|Q0C3X0_HYPNA   DDS.R.L............................LTSATLADLQSIVVEEGYT.......IL....ST.G..N.D..G.E.V.SVRA
gi|123323499|sp|Q0C3W9|Q0C3W9_HYPNA   ...................................MRSVFLADLKAVVAQAGYT.......IS....SV.G..D.N..G.A.D.SVRG
gi|122544362|sp|Q2NA07|Q2NA07_9SPHN   SAS.R.T............................LNSFDYDTLRATVTEIGGT....I..E.....PGQN.........D.G.F.LI
gi|123736226|sp|Q2G8G7|Q2G8G7_NOVAD   EAA.T.T............................VTAIRPDGVAEALTNLGYT....A..E.....LAKD....AN.G.D.P.L.IN
gi|122459425|sp|Q1YFZ1|Q1YFZ1_9RHIZ   QAQ.T.L............................VKPGEVDQIVDIARGFGSG.......T.....LEKD....E....T.E.Y.VR
gi|122351524|sp|Q0G7X7|Q0G7X7_9RHIZ   SGP.S.I............................VSTNEVDAIRELAEGYGDA....R..V.....VELD.....N.G.DP..A.IV
gi|123004239|sp|Q2IXX8|Q2IXX8_RHOP2   MSD.T.L............................ISKLDLDGLRELFQAAGYR....V..E.....TASD..P.VA.S.L.T.Y.LR
gi|122476043|sp|Q213H6|Q213H6_RHOPB   MPD.T.N............................IDKLSLDSLREVLQQAGYR....V..E.....TVTD..P.IA.N.V.A.Y.LR
gi|122295500|sp|Q07KZ5|Q07KZ5_RHOPA   MSN.H.T............................IAKMSLGGLRDIFQQAGYR....V..E.....TVTD..P.VA.N.V.D.Y.LR
gi|122403190|sp|Q1I9R8|Q1I9R8_9PSED   TEV.T.L............................IETVSADSLTKLLQDAGCR....V..N.....RSEQ..N....A.V.V.Q.LL
gi|122257709|sp|Q02HI9|Q02HI9_PSEAE   NDI.T.L............................IEHLSVDSLTEALQEAGYR....V..N.....RSEQ..N....G.V.V.Q.LL
gi|81415193|sp|Q745S9|Q745S9_THET2    LAQ.G.V............................RTGITPGEMEALLKAGSYR.......YE....RVEE....G..G.R.V.Y.FH
gi|81596955|sp|Q5N656|Q5N656_SYNP6    NDL.I.T............................PDNVSKDLLKAIYDAAFIE.......TA....WDDD.G.......D.....LK
gi|123557011|sp|Q31PB3|Q31PB3_SYNP7   NDL.I.T............................SENVSKDLLKAIYDAAFME.......TA....WDDD.G.......D.....LK
gi|81709081|sp|Q7NG79|Q7NG79_GLOVI    A...E.L............................HLATHREEVEAVIASLAMEGSVRFAQLK....ES.D..K....G...L..MWI
gi|123506214|sp|Q2JU30|Q2JU30_SYNJA   L...M.E............................EPPRYVEVVSAVVSSLKED.A...AY.E....NH.E..Q....G...H..TWK
gi|123556836|sp|Q31NC7|Q31NC7_SYNP7   S...A.T............................APLDYIDIIETVISSLADADS...AQVS....HT.G..S....G...T..IWK
gi|81670584|sp|P73244|P73244_SYNY3    D...E.M............................TPASHHDVVETVISGMAQENS...AFVQ....DN.D..Q....G...S..IWK
gi|122663130|sp|Q4C8F6|Q4C8F6_CROWT   F...T.S............................TEISHQEAIETVIDSLQENDS...AMVH....HD.E..Q....G...Y..LWK
gi|81771933|sp|Q8YVL5|Q8YVL5_ANASP    T...E.T............................ASINHVEVIENVIDSLEQDDS...AMVS....HTPE..G....G...Y..LWK
gi|123351931|sp|Q10XZ9|Q10XZ9_TRIEI   E...D.E............................TSLGYIENIETVIAGMAEEQK...VMVA....QN.E..A....G...H..LWK
gi|81596544|sp|Q5N4K3|Q5N4K3_SYNP6    ...................................QVAEISPLLIELFGAD.....RLE....AN.P..P....E...S...WQ
gi|123608251|sp|Q3M567|Q3M567_ANAVT   ...................................TPEVIAQTLAELFSTA.....DVQ....AI.A..P....G...S...WQ
gi|81708722|sp|Q7NEZ9|Q7NEZ9_GLOVI    ....................................................MAGG....TLS....EM.A..P....G...Q...WQ
gi|298346581|ref|YP_003719268.1|      FSV.N.R............................LPNLTRDRVKKVLESHKWS.......YQ....VNHD.G.......D.....VG
gi|260576430|ref|ZP_05844420.1|       .AV.SGD............................VLADNPAAIAELMRNFGYR.......AN....LGTD....DQ.G.D.P.K.ID
gi|296129402|ref|YP_003636652.1|      HAT.T.T............................PSPVSPARIASWMSDNQFS.......YF....IDND.G.......D.....LG
gi|254464399|ref|ZP_05077810.1|       ASA.Q.N............................VVASTGDSVADFFKDEGAK.......VE....LTTD....SV.G.D.P.N.VK
gi|269795609|ref|YP_003315064.1|      APE.P.G............................VTPVTRARVIEWITDYGYS.......YF....VDSD.G.......D.....VG
gi|167648377|ref|YP_001686040.1|      AQA.I.H............................NDGMTGPEVAAWLQKGGYK....A..E.....LTKD....DG.G.D.P.L.IN
gi|302865994|ref|YP_003834631.1|      GHP.Q.E............................LRPLTGELIAAVLGHRGYA.......VV....EEPD.G.......A.....LV
gi|254474961|ref|ZP_05088347.1|       AQA.Q.N............................IVAKNATSIANFFDEEGVA.......FE....VTTD....DV.G.D.P.K.IK
gi|238063407|ref|ZP_04608116.1|       GHP.G.A............................LRPLTDELIAAVLRHRGVT.......VA....ADPV.G.......R.....LV
gi|227495918|ref|ZP_03926229.1|       PSY.N.P............................LAPLSLDRVAKVLEDDELK.......YG....YDED.G.......D.....LA
gi|269219692|ref|ZP_06163546.1|       DGA.A.T............................LAPLSQERIVAALERDKII.......YA....YDSD.N.......D.....LA
gi|145593940|ref|YP_001158237.1|      GYP.G.A............................LSPLSGELIATVLTHRGYL.......VE....TDPA.G.......E.....LV
gi|269976471|ref|ZP_06183456.1|       LER.N.I............................VSPLNRARVKKILERNGWS.......YQ....VGNE.G.......D.....IA
gi|217969472|ref|YP_002354706.1|      FAD.A.L............................IDATKPERLYEIARGFGSA........E....LDKD....SQ.G.D.P.R.IT
gi|399991304|ref|YP_006571544.1|      GHA.E.N............................ILAKDATTLARFFETEGVE.......FE....VTTD....DV.G.D.P.K.LK
gi|256832278|ref|YP_003161005.1|      ADA.S.V............................PIPLSQDHITTWLTAHDLP.......YF....IDSD.G.......T.....AG
gi|225022571|ref|ZP_03711763.1|       SGE.D.V............................VQPLSADRIAAYLKAKELM.......FN....RDDD.G.......D.....VF
gi|126738030|ref|ZP_01753751.1|       VQA.Q.N............................IVATTAYSAKSFFENEGAE.......VE....ATTD....NV.G.D.P.K.LK
gi|254294753|ref|YP_003060776.1|      VRG.G.I............................LDASDANRLSRFMTSLGYQ....A..D.....MATG....PA.G.D.P.I.IS
gi|149184640|ref|ZP_01862958.1|       PLA.A.K............................NIVADVDQIANLLRAEGYK....A..K.....VEGE....G..G.D.R.H.IK
gi|257068239|ref|YP_003154494.1|      DSA.D.D............................LAPLSLTRVEESLTRHGYA.......FV....EDEE.....H.P...E.I.LR
gi|229820926|ref|YP_002882452.1|      ...................................MRAVDRKRVQTALDALGYR.......YF....VDSA.G.......E.....VG
gi|227494902|ref|ZP_03925218.1|       KDK.D.L............................VYPLSKERLQALFEAEGYK.......YY....VDSD.G.......D.....LG
gi|220934858|ref|YP_002513757.1|      ADQ.T.I............................ISSTNAKQVEGLLKELGFTG.....TRI....DE.D..D........D...VI
gi|291295614|ref|YP_003507012.1|      KRQ.H.Q............................TVEFNRALIRQYVEERGLR.......YL....TDQD....GD.Y.V.A...IM
gi|296130820|ref|YP_003638070.1|      PAP.P.A............................LGPLSRDRITGALDARDMQ.......YG....IDDD.G.......D.....VG
gi|84685465|ref|ZP_01013363.1|        PMS.D.R............................VIASDPQTFVAFFEDAGMP.......AR....LTED....TV.G.D.P.L.VE
gi|229820927|ref|YP_002882453.1|      RTS.G.G............................VRPVTSERLEDHLRDRRLL.......VS....RR.E..D....G.V.....LL
gi|227496193|ref|ZP_03926499.1|       PDA.Q.Q............................VRPLTRERVQELLERHGWV.......YE....TDCD.G.......D.....LV
gi|154507942|ref|ZP_02043584.1|       ......M............................VRPITQERLGLLFDSEGWA.......WR....IDSDG........D.....LC
gi|86137245|ref|ZP_01055823.1|        VQA.E.N............................LTARHSSSIEGFFLDEGSS.......VE....VLVD....NV.G.D.P.Q.LN
gi|227494903|ref|ZP_03925219.1|       IPT.G.E............................LAPLTLERVTQHFDEEGYN.......YG....IIDADPA.E..GSR.A.K.IE
gi|152982702|ref|YP_001354063.1|      PAV.E.L............................LNTVNAEQVSDAIKAAGCA....V..T.....TTEQ..D....G.T.T.W.LH
gi|119486978|ref|ZP_01620850.1|       E...P.E............................TTITYTEEIETVIVGLAKDQK...VMVG....QS.D..A....G...H..LWK
gi|145596051|ref|YP_001160348.1|      DAS.A.T............................VAPVTLSRICAALDGLDVR.......YL....ADGD.......G....N..LL
gi|220903645|ref|YP_002478957.1|      ADS.D.I............................ITASDQEKILEIAKGFG.S.......AE....LSST....SK.G.T.P.L.IQ
gi|297566041|ref|YP_003685013.1|      ..Y.T.D............................ASPLTADELRVVLDQLHYQ.......VE....RLEE....D..R.F.S.I.V.
gi|227496194|ref|ZP_03926500.1|       DSS.Q.V............................LAPLSQDRLLALFKERDWR.......YF....IDSD.G.......D.....LG
gi|119385461|ref|YP_916517.1|         PAA.QAQ............................VMGD.PEVIRLMMMDFGLP.......TK....LTTD....AA.G.D.P.L.IE
gi|183220878|ref|YP_001838874.1|      KSA.T.L............................HHKVEKNLIKKLLVELGYQ.......II....S..D....ED...K.L...LI
gi|291302096|ref|YP_003513374.1|      VPT.Q.K............................SAELTDERLIASLRRLNIR.......YL....VDEA.......G...A...LL
gi|387905553|ref|YP_006335891.1|      AHD.A.P............................IEAVSAERLAEILRRAGYR....V..T.....VAEQ..N....G.A.M.Q.LM
gi|238927566|ref|ZP_04659326.1|       GDV.E.S............................AGMGIRKAIRAELDRNEWN.......YE....ESEE..SDETRK.E.A.Y.FY
gi|322420651|ref|YP_004199874.1|      ....M................................AQNALAFEEYLKSQEIS......LEK....NTHE.....D.N...T.M.YV
gi|225629038|ref|ZP_03787072.1|       VPE.Q.I............................ILEFSMPTLLQFLQGEGWC....V..R.....PDQH.....SL..F.P.Q.II
gi|227495837|ref|ZP_03926148.1|       PVP.A.V............................PSPVDVDRVLACVRDLGLR.......YF....VDDE.G.......D.....IG
gi|291295390|ref|YP_003506788.1|      ..D.T.V............................LREVTTEEVRILLDQFGLN.......AE....QVKE....D..L.F.N.L.E.
gi|302381558|ref|YP_003817381.1|      AAP.G.T............................WKGLTVAEITGQLTASGLV....V..E.....APQ.....AQ.G.D.RVY.IP
gi|229589918|ref|YP_002872037.1|      ..S.E.L............................ITSVTTQRLTELLQEAGYR....V..N.....PSEQ..N....G.V.V.Q.LL
gi|229917768|ref|YP_002886414.1|      ....................N...............FEEKVESFRALAAERNMT.......FK....ESDT....E..T.H.V.T.FL
gi|304320687|ref|YP_003854330.1|      NPT.S.I............................VPSLHAEALLPILDELGLD....Y..Q.....GATL....PD.G.R.RII.LA
gi|154508096|ref|ZP_02043738.1|       EDE.V.T............................PYPADFERVVQAVREMGYA....L..D.....VIEK.........GR..A.AG
gi|172058831|ref|YP_001815291.1|      ..DQD.RNSKDTNDNKVTPINQNSQPETNENGVTAVQQQYLEEFQAFLVEKGIP.......ME....AREN....E..T.H.V.F.FM
gi|85705365|ref|ZP_01036464.1|        TPP.A.P............................EPPMTLERLDTIIRALDPE....A..E.......SN.....G.S.....V.WQ
gi|119512017|ref|ZP_01631112.1|       S...D.T............................VSVNHVEVIENVIGTLEQDDS...AMVS....HNPK..D....G...Y..LWK
gi|256397448|ref|YP_003119012.1|      ..A.N...............................VEMIRAFIARVLEDGGLV.......DG....RAEA..D.SD.G.D.Y.Q.L.
gi|94985189|ref|YP_604553.1|          ....E.T.............................ALLTLDTLAKYLREKEVQ.......LD....IEENNG....QR.....F.IR
gi|238927565|ref|ZP_04659325.1|       GSE.E.G............................LKNSAYEAIKAEFEKQGLN.......YG....ED....TSEDGE.D.H.I.LR
gi|304320540|ref|YP_003854183.1|      EKR.E.V............................HDTISAEELAAALTAAGLA....P..T.....VLTD..R.AT.G.A.P.V.VT
gi|163846985|ref|YP_001635029.1|      EEL.V.Y............................PAHVNRAWLFQLYNDAYFD.......VE....LDSD.G.......D.....VC
gi|121606820|ref|YP_984149.1|         SDF.I.P............................ESDVTPVTLSLHLERAVVQ.......HR....L......EDDEA.....I.YV
gi|121583407|ref|YP_973838.1|         SIL.I.E............................EKDVTAVNLAVELEQAVIG.......HV....L......DEDKS.....I.YV
gi|167856646|ref|ZP_02479340.1|       KMV.E.V............................YTKFSDQQLIDIVKKKYPK.......VE....LIEK....GR.I.R.....IT
gi|283780877|ref|YP_003371632.1|      AAD.K.I............................IEKLSPDVVIEVLKAEGYK....D...LQHVPSTKE.....D.S.P.D.A.IF
gi|85708893|ref|ZP_01039959.1|        ARP.D.W............................VGRFDDQLMRMLMQDLRAT....W..T.....VEQS....AE.G.LTT.Y.RA
gi|304321485|ref|YP_003855128.1|      DQQ.R.I............................VETLSFGDILPVLQRYGVN....Y..Q.....VGDM....G..G.R.P.V.AK
gi|304321021|ref|YP_003854664.1|      IAG.G.V............................VRSLDLDQLSEAIATAGFL....P..T.....ISHS....AT.G.L.PYI.VA
consensus>70                          ........................................................................................
```

```
                                      40        50                                  60
E.amylovora-AmyR                    QNFDGVFDAKIDLMDGVI.......L.............................FSA.....LA....E.VK..P.....T
D.radiodurans-DR1245                M................GWRFEM..G....D..AAV.LVSV.NDGP..NNTS..R.....LEITC....VT....Q.K...TY...AD
S.elongatus-T110839                 F................R....Y..G....S..AEV.FVQL.SGHT..E..EDF......LTIWS....PV.....LP.LP.....VA
E.coli-YbjN                         QNFDGVFDAKIDLIDNTI.......L.............................FSA.....MA....E.VR..P.....S
gi|123253281|sp|Q1GTJ6|Q1GTJ6_SPHAL S................S....R..N....G..LKF.LVLF.MNCE..E..G..Q.....KCKTLQYYM...........GFS....D
gi|123129053|sp|Q0C3X0|Q0C3X0_HYPNA K................T....A..E....G..LVF.NVIG.TVCD..S..ENAD....GCLGI....NM....QVR.Y......DA
gi|123323499|sp|Q0C3W9|Q0C3W9_HYPNA V................T....A..D....G..LIF.NVDG.AVCE..N..EIRP....GCLGI....NI....NVR.Y......DG
gi|122544362|sp|Q2NA07|Q2NA07_9SPHN K................F....P..N....G..TAA.TATF.TAC..AT.....G.....KCLGT....NI....S.A..RFGKPSDK
gi|123736226|sp|Q2G8G7|Q2G8G7_NOVAD A................D....I..G....G..WQA.ALMF.YECN..E..KT.H...D.GCQSL....QF....V.A..NFT....P
gi|122459425|sp|Q1YFZ1|Q1YFZ1_9RHIZ G................R....I..D....G..TLY.VIFF.NDCNS.D..R..S.....ECQSLQFYA............SWD.....
gi|122351524|sp|Q0G7X7|Q0G7X7_9RHIZ G................D....I..N....G..TAY.QLFF.LECE..D..G..K.....DCGAL....NFYAIW.......D.....
gi|123004239|sp|Q2IXX8|Q2IXX8_RHOP2 S................A....T..N....G..LAF.DIRP.GNRLSDG.....Q.....GIIDI....AL....V.A..VIQ....V
gi|122476043|sp|Q213H6|Q213H6_RHOPB S................A....T..N....G..LAF.DIRA.GNRLADG..S..A.....EFVDI....AF....T.A..ILQ....V
gi|122295500|sp|Q07KZ5|Q07KZ5_RHOPA S................A....T..G....G..LAF.DIRP.GNRLSAA..D..D.....SFVDV....AF....T.A..VLQ....I
gi|122403190|sp|Q1I9R8|Q1I9R8_9PSED S................A....S..Q....G..VGY.AVRF.GNRAQGQ..E..G.....EFLDF....TF....S.C..ALR....I
gi|122257709|sp|Q02HI9|Q02HI9_PSEAE S................A....S..Q....G..IGY.AVRF.GNPA.AT..P..A.....SYLDF....TF....S.C..ALR....V
gi|81415193|sp|Q745S9|Q745S9_THET2  L................R....L..A....G..LRA.VLFL.QDCR..E..G..S......CESLLLYA...........GFS....T
gi|81596955|sp|Q5N656|Q5N656_SYNP6  I.....................K..D....E..LNC.TVMI.S.EN..Q..T..R.....IMIFT....IF....E.F..EEG....F
gi|123557011|sp|Q31PB3|Q31PB3_SYNP7 I.....................N..E....D..IGC.SVFI.S.DN..Q..E..K.....ITFLR....VF....R.F..EEG....V
gi|81709081|sp|Q7NG79|Q7NG79_GLOVI  F................K....Y..G....T..VRV.FVVM.SGES..N..DDT......LTVWA....PV.....MK.LPEN...SA
gi|123506214|sp|Q2JU30|Q2JU30_SYNJA F................T....Y..G....S..VEV.FVHL.SGES..P..EDT......LLVWS....PV.....LS.LP.....VA
gi|123556836|sp|Q31NC7|Q31NC7_SYNP7 F................R....Y..G....Q..LEV.FVQL.TGSS..E..DDL......FTVWA....KV.....FP.LS.....ES
gi|81670584|sp|P73244|P73244_SYNY3  F................A....Y..G....S..VEV.LVQL.TGEG..E..NDL......FRVWA....EV.....MP.LP......T
gi|122663130|sp|Q4C8F6|Q4C8F6_CROWT F................Q....Y..G....S..VET.YVQL.TGEK..E..EDL......LTVWS....PV.....LT.LP.....AQ
gi|81771933|sp|Q8YVL5|Q8YVL5_ANASP  F................K....Y..G....T..VEV.FVQL.TGKS..D..EDT......ITVWS....VV.....LK.LP.....AK
gi|123351931|sp|Q10XZ9|Q10XZ9_TRIEI F................K....Y..G....S..VDV.YVRL.TGET..D..DDN......LTVWS....YV.....LK.LP.....AK
gi|81596544|sp|Q5N4K3|Q5N4K3_SYNP6  I................Q....T..P....E..CRL.LLLL.SASG.......EW.....LRVLL....PL.....LP.AV.......
gi|123608251|sp|Q3M567|Q3M567_ANAVT V................D....T..S....T..FRL.LVLL.SEDS.......SW.....LRVLL....PI.....VP.LQ.......
gi|81708722|sp|Q7NEZ9|Q7NEZ9_GLOVI  I................E....T..G....Q..WRL.LVLT.SVER.......DW.....LRMMI....PL.....VP.QE.......
gi|298346581|ref|YP_003719268.1|    G................A....W..Q....N..GIY.YFQV.TGEK..D..T..I.....LCVRG....TW....R.G..EPE...LD
gi|260576430|ref|ZP_05844420.1|     S................S....S..G....G..SNF.SIYF.YGCT..N..G..K.....ACDSIQFSS...........GFD....L
gi|296129402|ref|YP_003636652.1|    G................L....W..R....G..RLF.YFFL.FGQQ..A..E..I.....LQVRG....QW....H.R..ELA...IE
gi|254464399|ref|ZP_05077810.1|     V................E....Y..Y....G..SDF.SVYY.YGCG..N..N..T.....DCSAIQFFS...........GYQ....T
gi|269795609|ref|YP_003315064.1|    G................L....W..H....S..RLF.YFLL.FGAQ..T..E..I.....LQVRG....QW....N.R..EIT...IE
gi|167648377|ref|YP_001686040.1|    S................A....A..E....G..QTF.KIYF.YDCK..D..AR........CKAL....QF....S.A..GFD....L
gi|302865994|ref|YP_003834631.1|    G................R....W..E....R..NLI.WFHR.RGVA..G..E..L.....LQVRT....VV....A.D..HFG...IE
gi|254474961|ref|ZP_05088347.1|     V................D....Y..Y....G..NDF.SIYF.YGCT..D..N..T.....NCDAIQFFS...........GYQ....T
gi|238063407|ref|ZP_04608116.1|     G................R....W..D....D..GLI.WFLR.IGAA..G..E..I.....LQVRT....IA....A.P..TFP...IE
gi|227495918|ref|ZP_03926229.1|     C................G....W..D....T..GIF.FFMR.GGEQ..G..E..I.....LRIQG....RF....H.L..PLP...AD
gi|269219692|ref|ZP_06163546.1|     A................G....W..E....H..GGF.FFLI.GGES..D..E..Y.....LRVQG....RW....Y.A..SLS...TD
gi|145593940|ref|YP_001158237.1|    G................R....W..A....D..SLI.WFLR.PGTA..G..E..L.....LQVRT....VV....A.P..TFP...IE
gi|269976471|ref|ZP_06183456.1|     G................A....W..E....N..GIY.HFEV.TGNR..D..S..V.....LCVRG....TW....R.G..KLE...LD
gi|217969472|ref|YP_002354706.1|    G................R....I..E....G..TKY.GIYF.YGCV..K..G..A.....DCDDIQFSA...........SWS.....
gi|399991304|ref|YP_006571544.1|    V................D....Y..Y....G..NDF.SIYF.YGCE..N..N..K.....NCDAIQFFS...........GYQ....T
gi|256832278|ref|YP_003161005.1|    A................I....H..T....A..RIF.TFAL.IGQH..K..E..V.....LHIRG....RW....N.R..HAT...IE
gi|225022571|ref|ZP_03711763.1|     I................N....F..D....G..NGF.VLLA.TGEK..R..E..I.....LVVRG....AW....Q.V..TAP...IE
gi|126738030|ref|ZP_01753751.1|     V................N....Y..Y....G..NEF.SVYY.YGCD..N..N..S.....DCNAVQFFS...........GYK....T
gi|254294753|ref|YP_003060776.1|    G................R....I..S....S..SDY.TIHF.YECE..N..GE.F......CNSI....QF....L.A..DTP....I
gi|149184640|ref|ZP_01862958.1|     T................G....M..A....G..YSF.LILP.YDCN..D..KG.D...A..CKSV....QF....Y.K..AFT....P
gi|257068239|ref|YP_003154494.1|    AR...................F..D....D..YRF.QFMV.SGDE..H..G..V.....LQTRG....RW.....SH..SVD...VS
gi|229820926|ref|YP_002882452.1|    G................L....W..D....N..RLF.SFYL.IGSG..A..Q..M.....LQVRG....RW....P.R..RVA...LE
gi|227494902|ref|ZP_03925218.1|     G................F....W..D....F..NTF.HFVL.AGEN..Q..E..L.....LHILC....RS....R.R..VLT...MK
gi|220934858|ref|YP_002513757.1|    V................M....M..H....G..RPV.VVIVGSGNG......RQ.....LQMNV....AF.....V..........
gi|291295614|ref|YP_003507012.1|    A................M....E..H....CPQKLF...TI.FAAS..G..L..R.....E.DVFSITMRV....E.P..SPE....M
gi|296130820|ref|YP_003638070.1|    G................Y....W..D....G..HLF.YFFL.LGGN..G..E..F.....LQTRG....RW....N.R..KVG...PD
gi|84685465|ref|ZP_01013363.1|      Y................R....S..N....G..DKM.SLFF.YDCE..D..N..V.....DCQAVQFYA...........GYR....A
gi|229820927|ref|YP_002882453.1|    G................E....W..Q....D..MPF.SIGL.GGRE..D..SVL.......QVRG....RW.....PRL.LP.....AR
gi|227496193|ref|ZP_03926499.1|     G................R....W..D....D..DLL.TFMV.RGES..G..E..M.....LNVMG....YM....L.E..DLP...LE
gi|154507942|ref|ZP_02043584.1|     G................F....W..E....G..HLF.CFRF.LGDS..R..E..V.....LSIVA....FM....K.S..LVP...IE
gi|86137245|ref|ZP_01055823.1|      V................E....H..Y....G..SEF.TIFY.YGCE..D..N..T.....NCDSIQFYS...........GYA....T
gi|227494903|ref|ZP_03925219.1|     S................G....W..D....G..IPF.LFNF.NGQE..G..E..I.....LTVFA....QS....S.V..DIP...LD
gi|152982702|ref|YP_001354063.1|    S................A....S..H....G..IGF.QVLW.GNA..IT..P..G.....QYADL....TL....S.C..PLR...VQ
gi|119486978|ref|ZP_01620850.1|     F................Q....Y..G....S..AEV.FVQL.TGDT..E..DDT......LTVWS....PV.....LQ.LP.....AK
gi|145596051|ref|YP_001160348.1|    AM...................W..E....R..HAV.LVAL.EGPD..D..E..I.....LVLRV....RP.....HA..TVP...PD
gi|220903645|ref|YP_002478957.1|    G................R....M..D....G..IKY.AIWF.SGCT..D..G..K.....SCSALQFIG...........MWK....T
gi|297566041|ref|YP_003685013.1|    .Y....................T..T....G..LKA.SLVT.YGEP..S..K..L.......RSL....QL....R.A..GFT...GF
gi|227496194|ref|ZP_03926500.1|     G................I....W..D....D..ATF.YFNL.GGQK..Q..E..V.....LRIWA....QL....P.G..TID...AS
gi|119385461|ref|YP_916517.1|       S................R....I..D....G..TSF.QVYF.YQCE.........K.....TCAAIQFSA...........GFD....L
gi|183220878|ref|YP_001838874.1|    V................S....Y..Q....D..FRV.GLVS.GN.................DTSIQFYS...........SFN....T
gi|291302096|ref|YP_003513374.1|    AM...................W..E....R..HVV.QIRR.EGPA..G..E..I.....LVLRA....RA.....YS..TLP...AE
gi|387905553|ref|YP_006335891.1|    S................A....S..Q....G..VGF.AVRF.GNPAVGV.PPA.IDAARIVYLDY....TL....S.C..VLQ....V
gi|238927566|ref|ZP_04659326.1|     MRNE............LEN....D..E....P..VTV.VIAV.REYN..H..A..D.....GFIKI....KIYD..I.AYLEES....S
gi|322420651|ref|YP_004199874.1|    IR.....................DNLE.EVGP..VSL.MALF.NDSD..R..Y..V.....T.LIC....YK.....YF..AFP...AE
gi|225629038|ref|ZP_03787072.1|     A................S....L..P....D..VEC.IIRF.GTLA..R..SG.T...G..WSDF....TL....S.A..PFI.....
gi|227495837|ref|ZP_03926148.1|     I................P....W..R....Y..VTV.HVIF...QD..D..R..A.....IQLRG....VW....H.R..ISD...TE
gi|291295390|ref|YP_003506788.1|    .L....................P..N....G..LRG.HLIT.YGG...S..H..V.......SSL....QL....R.A..GFS...GF
gi|302381558|ref|YP_003817381.1|    V................T....D..G....A..VRS.IVTL.FSCS..D..G..A......CPDV....QF....T.A..AFA.....
gi|229589918|ref|YP_002872037.1|    S................A....S..Q....G..IGF.AVRF.GNPA.AE..Q..G.....SYVDF....TY....S.C..ALR....I
gi|229917768|ref|YP_002886414.1|    TR...................E..TTKSGA..QPVMIIAF.NKKT..T..D..A.....E.LYA....AT....V.T..HVPD..YV
gi|304320687|ref|YP_003854330.1|    A................A....P..N....G..LKF.QLTP.TACE..N..NN.K.....RCRGL....HL....L.S..LFE.....
gi|154508096|ref|ZP_02043738.1|     A................I....F..D....E..IPF.LVSF.D..A..A..GR.F.....LSIRA....LWESDL........P...AE
gi|172058831|ref|YP_001815291.1|    TQ...................E..KTPSGA..EPMMMIVF.SKTA..T..D..V.....E.LFA....RT....I.T..HVPD..GV
gi|85705365|ref|ZP_01036464.1|      L................T....V..N....D..VQV.LIVT.DESA..D..R..........M.....RAMTPVA.K..A.T....D
gi|119512017|ref|ZP_01631112.1|     F................K....Y..G....S..VEV.FIQL.TGES..D..EDT......ITVWS....AV.....LN.LP.....AK
gi|256397448|ref|YP_003119012.1|    .R....................Y..G....S..ALY.WVSA.GE........A..D.......DGL....GLVQV.F.G..VVLED..VP
gi|94985189|ref|YP_604553.1|        M................GWRFEM..G....D..AAV.LISV.NDGP..NNTS..R.....LEITC....VT....Q.K...QY...AD
gi|238927565|ref|ZP_04659325.1|     MRQQ............LDN....G..S....V..VSI.AIVV.TENG..D..T..N.....DFIKI....KYFG..M.VRLDEK....S
gi|304320540|ref|YP_003854183.1|    G................I....S..D....G..LVF.VVRA.LECS..G..LP.A...R..CEQL....VL....F.A..NFD....L
gi|163846985|ref|YP_001635029.1|    L.....................R..Q....G..FRV.WVFP.I.RE..G..E..Q.....IRFMS....QF....R.A..NPD....R
gi|121606820|ref|YP_984149.1|       TED.............DW...............IPF.WIRI.L.....K..K..P.....GFVGF....VTYF..N.FRKS......S
gi|121583407|ref|YP_973838.1|       TED.............GW...............FPF.WIRI.Q.....E..S..A.....GYVTF....KTYT..N.FKKS......T
gi|167856646|ref|ZP_02479340.1|     S.....................D..S....G..IKL.V..I.YNQR........D......DGSLTFRM...........YFD....G
gi|283780877|ref|YP_003371632.1|    V................T....Y..E....G..RRV.LVVV.P.....N..G.........GDLL....QV....N.Y..FVE.....
gi|85708893|ref|ZP_01039959.1|      S................A....E..G....G..INF.VAVP.RSCE..D..Q..N.....GCVGL....VVIALFN.D..V.N....V
gi|304321485|ref|YP_003855128.1|    A................Y....M..G....G..TEV.TLVP.NVCT..A..DH.R......CAGL....SL....Y.A..FSP.....
gi|304321021|ref|YP_003854664.1|    E................K....A..N....G..ARL.FATL.GACDMPQ..AG.K...G..CQLI....EY....L.V..LMN....L
consensus>70                        ........................................................................................
```

```
                                         70          80         90                        100          110
E.amylovora-AmyR                     ...ALIPLAGD.LSQINASSLTVKAFL..D.....I...QD......DNLPKLIVCQSLS..AAAG...LTYGQFV.............
D.radiodurans-DR1245                 ...RRAEVAMM.LNDRNRERAFARS.I.D......Q.EG........NV....WLEYVGFYPTL..A.EMPQETFD.............
S.elongatus-T110839                  ...DELALYRK.LLTLNWLTTFEAHFA..I.....A...EE......QV....QVVASRT..L...G.GITAGEIS.............
E.coli-YbjN                          ...AVLPLAAD.LSAINASSLTVKAFL..D.....M...QD......DNLPKLVVCQSLS..VMQG...VTYEQFA.............
gi|123253281|sp|Q1GTJ6|Q1GTJ6_SPHAL ...AKDMPLER.FNQWNREKRFARAYK.......DD...AGD......P....VLEMDVD..LD..FAGIPRENVG.............
gi|123129053|sp|Q0C3X0|Q0C3X0_HYPNA ...DGKETLER.INDVNLMWAATSAWYSVG.....G...TDGK.T.PTV....GITRYVI..LD..R.GATIGNIK.............
gi|123323499|sp|Q0C3W9|Q0C3W9_HYPNA ...DDRVTYQK.INDANLMWPAVSVSV.EG.........NMGE.TGSTV....VITRYVI..LD..G.GMTMRNVS.............
gi|122544362|sp|Q2NA07|Q2NA07_9SPHN ...TDSQVAEL.VRDYNRRWSAGKSYL..T.....D...DG......RA....VVQAYMI..AD..G.GISMENYR.............
gi|123736226|sp|Q2G8G7|Q2G8G7_NOVAD ...EKKFTAED.AVKFMRNTRFASVSL..T.....Q...DQ......SV....TMTWDVV..TG..K.GIDLEVFS.............
gi|122459425|sp|Q1YFZ1|Q1YFZ1_9RHIZ ...GVPVDQAA.INGWNRDKRFAKAYV.......DA...END......P....VLEMDVN..LR...HGVTRGNLE.............
gi|122351524|sp|Q0G7X7|Q0G7X7_9RHIZ ...VPTVSVGA.LNVWNRTAPFNKAYL..T.....E...EN......RP....VIEMNLP..AE..G.GFVREQLD.............
gi|123004239|sp|Q2IXX8|Q2IXX8_RHOP2 ...QGELPLAI.VNRWNASRRFGRLQL..S.........GP......FL....ALSLDLL..LA..G.GVSRDHLR.............
gi|122476043|sp|Q213H6|Q213H6_RHOPB ...QGDLPLDL.VNRWNASRRFARLQL..S.........QP......FL....VLSLDVC..VA..G.GVAPNHLR.............
gi|122295500|sp|Q07KZ5|Q07KZ5_RHOPA ...QGELPLDL.VNRWNATRRFARLQV..S.........QP......FL....VLCLDVS..MA..G.GVTQDYLR.............
gi|122403190|sp|Q1I9R8|Q1I9R8_9PSED ...QGELPAGL.AERWNASRRFARLSV..Q.........GE......FL....VMEKDVV..VA..D.GVSEKHLL.............
gi|122257709|sp|Q02HI9|Q02HI9_PSEAE ...QGELPAGL.TERWNLSRRFGRLSQ..Q.........GE......FL....VMEMDVI..VA..G.GVSLANLR.............
gi|81415193|sp|Q745S9|Q745S9_THET2  ...DNPPSLER.VNEWNREKRFSRAYL.......DE...EGD......P....VLEADLD..LA...GGVADGAIR.............
gi|81596955|sp|Q5N656|Q5N656_SYNP6  ...SREQKLDL.ANKINDEYAFVRASL.T......E.YD........TL....LFDYSFY..LK..G.GLTKKNLV.............
gi|123557011|sp|Q31PB3|Q31PB3_SYNP7 ...SRADRLEF.VNRINDKYSFVRAML.T......T.YD........TL....AFDHDLF..IK..G.GISKKNLV.............
gi|81709081|sp|Q7NG79|Q7NG79_GLOVI  ...KTGELYAH.LLRSNWLETWEARFC..L.....R...EN......EV....ALHTMRT..L...D.SLDPAEIS.............
gi|123506214|sp|Q2JU30|Q2JU30_SYNJA ...EPAKLMQK.LLEKNWSETLEARFC..I.....W...NN......QV....VLNHHRT..L...E.GLSAGEIS.............
gi|123556836|sp|Q31NC7|Q31NC7_SYNP7 ...TDATLLQR.LLTLNWADTLEARFA..L.....Y...DD......HI....TMVASRT..V...A.DLSAGEIS.............
gi|81670584|sp|P73244|P73244_SYNY3  ...DPGQLLAE.VMQLNWSDTFEACFA..L.....R...EN......HL....VALHQRT..V...A.DLSPSEIS.............
gi|122663130|sp|Q4C8F6|Q4C8F6_CROWT ...DELGLMRK.LLEMNGEETLETRFG..I.....M...NN......QI....VVLTQRT..V...E.DLSPGEIS.............
gi|81771933|sp|Q8YVL5|Q8YVL5_ANASP  ...NEPKLLRH.LLELNCSSTFEARFG..I.....I...ED......QV....VVISTRT..L...A.ELSPGEVS.............
gi|123351931|sp|Q10XZ9|Q10XZ9_TRIEI ...NEPELMRK.LLQMNWLSTMESHFA..I.....V...DN......QI....VVVATRT..V...A.ELSPGEIS.............
gi|81596544|sp|Q5N4K3|Q5N4K3_SYNP6  ...DAAPFHRQ.ILEANFDATGPVRHA..L.....H...QN......VL....WGVFQHD..LA....SLTSGDLYQAIASLFDLAQR
gi|123608251|sp|Q3M567|Q3M567_ANAVT ...EAQAFLAQ.FLEANFDDTQEVRYA..L.....Y...DG......VI....WAVYQHN..SS....TLTQDDLSSAIARLISLYEA
gi|81708722|sp|Q7NEZ9|Q7NEZ9_GLOVI  ...EAMPLAAQ.LLEANFDRTREARFA..F.....Q...NG......LL....WGMYQHR..LS....TIGSEDLLAAVEQLQQLHDR
gi|298346581|ref|YP_003719268.1|    ...DFILINSI.CNRWNTEYYWPKAYA.R......V.TE.DRE.V..YV....HTELPIS..WR..N.GLTDAQLD.............
gi|260576430|ref|ZP_05844420.1|     ...TKGSSLQV.VNDWNRDKRYGKAYL.......DN...EMD......P....FIEMDFN..LD..FGGISDENFR.............
gi|296129402|ref|YP_003636652.1|    ...RLEEVLDL.CNEWNAERIWPKAYV.R......V.RD.NGR.V..HV....VAEVATD..LE..H.GATDAQLS.............
gi|254464399|ref|ZP_05077810.1|     ...DGSVRLAK.INEWNTENRFARGYI.......SE...EGA......A....RIEMDVF..LG..ADGMSPDDFA.............
gi|269795609|ref|YP_003315064.1|    ...RIEEVLDF.CNDWNTDRIWPKAYF.R......V.RD.NGM.I..QV....YAEVSVD..LE..H.GATDEQLD.............
gi|167648377|ref|YP_001686040.1|    ...KEPLKYEK.INEWNRKNRYLKAYL..D.....D...DG......DP....YVQYDIN..VN..A.GRTVSGLD.............
gi|302865994|ref|YP_003834631.1|    ...RVPELHAF.CNTWNHDRLWPKAYV.H......V.AD.DGS.A..QV....CGEVTTD..LE..R.GVTPHQLD.............
gi|254474961|ref|ZP_05088347.1|     ...DGGVRLSK.VNEWNTENRFGRAYI.......SE...EGS......A....RIEFDIY..LG..NDGMDPDDFA.............
gi|238063407|ref|ZP_04608116.1|     ...YVPTLHAF.CNAWNHDRYWPKAFV.H......V.DD.DGR.A..LV....CGEVIAD..LE..R.GVTPHQLD.............
gi|227495918|ref|ZP_03926229.1|     ...ELPKAMQA.CNDWNTRALWPKTFA.A......L.NN.ADQ.T..VV....MAEHNVD..FE..L.GATDEQIA.............
gi|269219692|ref|ZP_06163546.1|     ...RLGEAIQA.CNEWNIHTIWPKTYA.V......A.ND.VNE.V..VI....MTEHSVD..YE..H.GVTDAQIS.............
gi|145593940|ref|YP_001158237.1|    ...YVPALHAF.CNAWNHDRLWPKAFV.H......V.ED.DGR.A..RV....CGEVITD..LE..R.GVTPHQLD.............
gi|269976471|ref|ZP_06183456.1|     ...DFMLVSSL.CNRWNTEYYWPKTYA.R......V.TD.NRE.L..FV....HTELPIS..YH..A.GLTDSQLD.............
gi|217969472|ref|YP_002354706.1|    ...GPKVSLEK.INDWNRTKRFGKAYL.......DK...DGD......P....NLEMEVN..LD...YGVSAKNIE.............
gi|399991304|ref|YP_006571544.1|    ...DGGVRVVK.INEWNTENRFGRAYI.......SD...EGA......A....RVELDVY..LG..DTGMNPDDFA.............
gi|256832278|ref|YP_003161005.1|    ...RAEQIRAL.CNDWNTTKIWPKAYH.R......V.RD.DGI.I..EV....YGESNMD..FE..H.GATYDQVG.............
gi|225022571|ref|ZP_03711763.1|     ...MRDRLVDL.CNDWNRDKLWPKTYV.T......V.DD.AGA.V..RV....RTELNID..ME..Y.GASDLQLK.............
gi|126738030|ref|ZP_01753751.1|     ...DGSVRLSK.INTWNAENRYARAYI.......SD...SGA......A....RIEMDIF..LG..RDGVSADDFA.............
gi|254294753|ref|YP_003060776.1|    ...PPAMTMEK.VNAFNARWRYVRASL..T.....S....N......VV....RLQMDVN..LD..G.GVTAGNIE.............
gi|149184640|ref|ZP_01862958.1|     ...KDKPTLEE.MNSYAAENRFGRIYL..D.....Q...DR......DP....AIEMDID..LE.AG.GMSKELFI.............
gi|257068239|ref|YP_003154494.1|    ...RKVQMVKL.CNEWNMNRIWPKVYV..R.....R.E.SEGL.L..GV....YGELAAD..FR...AGALDSQID.............
gi|229820926|ref|YP_002882452.1|    ...RLGAMLEF.TDAWNRERIFPKCYV.R......V.LD.DGM.V..HV....TTEVSVP..IQ..H.GLNDVQLE.............
gi|227494902|ref|ZP_03925218.1|     ...YLDVVRAK.IKEFNAEKIFPTCFY.R......I.SD.EGL.L..TV....HTQLTYD..WE..H.GVSDSQLE.............
gi|220934858|ref|YP_002513757.1|    ...GMGITLEQ.VNAWNRNRILAKAYL..D.....R...DG......DT....IFESDLD..LD..G.GITVDRLK.............
gi|291295614|ref|YP_003507012.1|    ...SELEALRL.VNRWNTQRRWPRAYH.......TE...DG.......F....TLDWHID..LE...MGISPALFA.............
gi|296130820|ref|YP_003638070.1|    ...QLGRLLEL.VNEWNATHLWPKGYV.R......L.ED.E.V.V..GV....YAEHTVD..YE..H.GVADAQVD.............
gi|84685465|ref|ZP_01013363.1|      ...EN.VTLET.INSWNTDRRFVRSYL.......TD...EGV......A....RIEMDVA..TS..NDGLSTGDFD.............
gi|229820927|ref|YP_002882453.1|    ...AGAGLAQL.INDWNRDRVLPKVYS..V.....P...EGDA.V..AV....VAETNLG..VR..F.GASDEQLT.............
gi|227496193|ref|ZP_03926499.1|     ...RLDEVRFA.LEDWHREHIWPTCFW.R......E.ND.DALTF..SV....GASHMVD..WE..H.GVADCQLE.............
gi|154507942|ref|ZP_02043584.1|     ...CGEDLRDF.LQAWHGEFLWPKAYV.A......D.QD.EG..D..RV....VAEVNAD..YE..Y.GATDAQLV.............
gi|86137245|ref|ZP_01055823.1|      ...DGSVRLKT.VNDFNAEKRWVRAYV.......AD...NGS......T....KLEMDLY..MG..KDGISADDFA.............
gi|227494903|ref|ZP_03925219.1|     ...AEDQLDAF.IENWHREHYFPKVYT.R......R.ASADTA.L..RV....CCEHSID..LE..H.GVTDKQLA.............
gi|152982702|ref|YP_001354063.1|    ...GGVLPEGV.LTEWHRTKRFARVAL..H.........GD......FV....VLEMDVM..VA..G.GVSPAHLA.............
gi|119486978|ref|ZP_01620850.1|     ...NEATLMRK.LLEMNWLSTYESHFA..I.....N...NQ......QV....IVLSNRT..L...A.GITPAEIS.............
gi|145596051|ref|YP_001160348.1|    ...WEDRAYRV.VNEWNHTRRFCKAYI..G.....D.PSERGQ.L..PI....YAELQVP..FA...AGVHDALLV.............
gi|220903645|ref|YP_002478957.1|    ...E.DFPMAE.VNKWNAQKVYTRTYL.......DK...DQD......L....TMEMDVF..MR...YGMTQKNLE.............
gi|297566041|ref|YP_003685013.1|    ...NRIE.LRH.INTWNRRYRFSKAYL..D.....G...DN......DP....VLEFDLW..LE....GASPELIM.............
gi|227496194|ref|ZP_03926500.1|     ...HLEQVRTV.LDTSHRRSAWPTACY.R......I.DD.DGE.V..RV....FACHAVD..YE..Y.GLSDLQLA.............
gi|119385461|ref|YP_916517.1|       ...DDSMSESM.ANRWNREKRFGKVWL.......DE...TGD......P....FIEMDIG..VA..GDGIGRKNFD.............
gi|183220878|ref|YP_001838874.1|    ...DKKNKMEL.ANKWNQKMRYSRSYM.......DA...EGR......L....VLESDFD..YS...GGVSEEAIK.............
gi|291302096|ref|YP_003513374.1|    ...WNDRAFRA.VNEWNRTRRFLKAYV..G.....E.PTESGS.V..PL....YGETQVP..LR...PGVPDDLLD.............
gi|387905553|ref|YP_006335891.1|    ...QGELPAEL.VANWNRTKRFARLAS..H.........GA......FL....ALEMDVV..VA..G.GVSERHLR.............
gi|238927566|ref|ZP_04659326.1|     ...NRAELLAK.LNGWNAEYRYVKFCL..D.....S...DQ......DV....VVDIDLP..LDLHKGEFKPNAVI.............
gi|322420651|ref|YP_004199874.1|    ...KKPAVLEM.INTLNAEYTMVK.YV..E.....T.G.N.......AL....SIQIVVP..FH...DNFSSEVIV.............
gi|225629038|ref|ZP_03787072.1|     ...VDSEVSPFIGALWNRKNRFGRVYR..I.....N...K.......SL....FLEMDVV..LE..G.GVTQTYLQ.............
gi|227495837|ref|ZP_03926148.1|     ...HLARLRGL.VEEWNATRIGPKAYL.T......I.AD.GGI.V..RL....HGEVTYP..LG..A.GMTDAQLE.............
gi|291295390|ref|YP_003506788.1|    ...DRVEPRHL.LN.WNRRYRFSKAYF..D.....A...DN......DP....VLESDLR..LE....GVTPEAIH.............
gi|302381558|ref|YP_003817381.1|    ...GPQATPDI.VTRWNAERRFVKAFY..AVSGEAE...EG......QA....VAQYDIL..LV..P.AVGPSQLD.............
gi|229589918|ref|YP_002872037.1|    ...QGELPEGL.AQVWNASRRFARLSV..Q.........GE......FL....LMEMDVV..VA..G..VGATHLR.............
gi|229917768|ref|YP_002886414.1|    ...EDLPILNA.LNEFNQEFKYFRCVL..D.....S...DR......DV....TLASTLD..LD..L.GFSPEIIL.............
gi|304320687|ref|YP_003854330.1|    ....TNAPSRT.VAAFNYRYAFVSTGV..D.....D...SG......VA....YVSRYDI..AD..Y.GTPKGNIA.............
gi|154508096|ref|ZP_02043738.1|     S..AEPALFAT.ADNWNREKYFPTVYT..A.....TSP.EGT..L..GV....YADFVVD..TE..A.GLSDVQLR.............
gi|172058831|ref|YP_001815291.1|    ...EDLPILNA.INDFNQEFKYFRVVL..D.....S...DR......DV....TIASTID..LD..H.GFNPAAIF.............
gi|85705365|ref|ZP_01036464.1|      ...LTPEDLNR.ILQANFDTALDARYA..I.....A...KD......LL....WAAFIHP..LK....PLEKDEFI.............
gi|119512017|ref|ZP_01631112.1|     ...EELKLMRH.LLEMNCSNTLEARFG..I.....I...EN......RV....VVISTRT..L...E.DLSPAEVS.............
gi|256397448|ref|YP_003119012.1|    ...LSKKLLTA.VNEINSGYLWVRCYW........A...QG......RL....TVARDLA..AD....TLTADQLT.............
gi|94985189|ref|YP_604553.1|        ...RRQEVMAL.LNDRNRERAFSRS.I.D......A.DG........NV....WLEYVGFYPTL..A.EMPQETFD.............
gi|238927565|ref|ZP_04659325.1|     ...DPTVFHEK.LNEWNSEYRYVKFVV..D.....D...EQ......DV....VVDIDLP..LDLHDGVFQVDSFM.............
gi|304320540|ref|YP_003854183.1|    GRAVTDADFRV.VNGFNDSSVHGRAYV..L....EN...RR......QI....GVDFTID..MV..G.GVTSDHVD.............
gi|163846985|ref|YP_001635029.1|    ...DLADRILY.VNRINDELHIVRSYV.D......R.SG........DI....GFDGYLV..VS..G.GVTRRNII.............
gi|121606820|ref|YP_984149.1|       ...TSLQRLEL.ANEFNRETYMSSAFV........K...DD......IL....KITHVIS..YRDG...LLTETLI.............
gi|121583407|ref|YP_973838.1|       ...SHLQRLEL.CNELNAKNYLVTSCV........K...DD......HL....LFDYILN..YRDG...LLRETFI.............
gi|167856646|ref|ZP_02479340.1|     ...D.NTSLKS.VNKWNATKRFLTAYI.......DK...DGD......L....ALKDDLD..VE...EGISEAYLL.............
gi|283780877|ref|YP_003371632.1|    ...EHNATLKK.VNDWNRNHYITKACI..L.....S...DT......SV....SMGADLV..VA....GSKKTTMA.............
gi|85708893|ref|ZP_01039959.1|      D..NAARLDAF.INAFNDRQPTAKVMR..D.....P...QG......MV....ALQAYIN..AA..N.GITYRNLL.............
gi|304321485|ref|YP_003855128.1|    ....MAGTTAV.MTDFNRTTPVARVTP..G.....P...DG......GA....MLYHYVI..GD..H.GVTQGSLM.............
gi|304321021|ref|YP_003854664.1|    ....PASSPAL.VNNFNIERRTVSAGF..L.....E...GS......LT....GLTYRQV..LR..G.GVLAENVG.............
consensus>70                        .............n..n...................................................g....................
```

```
                                                                     120       130       140       150
E.amylovora-AmyR                      .......................................................HFMKESEEQISMIVMEAFANHLLMIAEDEERPP
D.radiodurans-DR1245                  .......................................................TLFGGVLMHFQDDYAALEG.YV....PQEGMQI
S.elongatus-T110839                   .......................................................RLITIVATLADDYDDALRA.EF....K.G....
E.coli-YbjN                           .......................................................WFVRQSEEQISMVILEANAHQLLLPTDDEGQNN
gi|123253281|sp|Q1GTJ6|Q1GTJ6_SPHAL   .......................................................ETFNTWASLMDSFRDHVFE..............
gi|123129053|sp|Q0C3X0|Q0C3X0_HYPNA   .......................................................DNLLNVLAIAPNAANYIWQ.AG....EYAPGY.
gi|123323499|sp|Q0C3W9|Q0C3W9_HYPNA   .......................................................DNLTNALAIATSVADYVWE.VG....DYAPGA.
gi|122544362|sp|Q2NA07|Q2NA07_9SPHN   .......................................................MQLVVYSQMLKKMRETIYK.DG...........
gi|123736226|sp|Q2G8G7|Q2G8G7_NOVAD   .......................................................NAVDMFRSAMDTLGTEVF...............
gi|122459425|sp|Q1YFZ1|Q1YFZ1_9RHIZ   .......................................................DTFDWWRLSLREFKSGVLK..............
gi|122351524|sp|Q0G7X7|Q0G7X7_9RHIZ   .......................................................YIFSQWTIALAEFSRDVIA.EA....P......
gi|123004239|sp|Q2IXX8|Q2IXX8_RHOP2   .......................................................AEIEIWDHLVQQLIVFLRE.EL....AGLAQP.
gi|122476043|sp|Q213H6|Q213H6_RHOPB   .......................................................AQIEIWDHLVQQLITYLRE.EL....PKLAPV.
gi|122295500|sp|Q07KZ5|Q07KZ5_RHOPA   .......................................................GQIEIWDQLVQQLIGYLRE.EL....KQLSVS.
gi|122403190|sp|Q1I9R8|Q1I9R8_9PSED   .......................................................GSLLLWDRLLQEFIVYLRD.YS....RNVAEQ.
gi|122257709|sp|Q02HI9|Q02HI9_PSEAE   .......................................................SHIELWDRLLQEFIAYLRD.YS....RLAAEQ.
gi|81415193|sp|Q745S9|Q745S9_THET2    .......................................................AFLDLFEENLRAFAAWIGW..............
gi|81596955|sp|Q5N656|Q5N656_SYNP6    .......................................................LGTRFFQSIPLEAIHEHGK.EM....VV.....
gi|123557011|sp|Q31PB3|Q31PB3_SYNP7   .......................................................LATRFFLSIPLDAINDCGE.EL....VA.....
gi|81709081|sp|Q7NG79|Q7NG79_GLOVI   .......................................................RAITIVATLADEYDEPLIE.QF....GGTAPF.
gi|123506214|sp|Q2JU30|Q2JU30_SYNJA   .......................................................RAITLVASLADEYDEQLQA.EF....PKA....
gi|123556836|sp|Q31NC7|Q31NC7_SYNP7   .......................................................RAITLVASIAATYAEGLP.........S.....
gi|81670584|sp|P73244|P73244_SYNY3    .......................................................RAITLVATLADDHDDRLKE.KY....GA.....
gi|122663130|sp|Q4C8F6|Q4C8F6_CROWT  .......................................................LAITLVATIADDNDEKLIE.TY....GCNSVT.
gi|81771933|sp|Q8YVL5|Q8YVL5_ANASP   .......................................................RIITIVATIADDNDEALQS.EF....GA.....
gi|123351931|sp|Q10XZ9|Q10XZ9_TRIEI  .......................................................RAITIVATLADDNDELLVA.EF....G......
gi|81596544|sp|Q5N4K3|Q5N4K3_SYNP6   GLDPFFTALAETQLRQIVRAAKQQGQSLPATLQTLTHLYEEGVLGDLSNGPEIRRFTLDRWREQLERLWPEVEV.D............
gi|123608251|sp|Q3M567|Q3M567_ANAVT  GLNDVFNRLIESRIRQIVQAAKQQGQSLEGTMQNLERFYAEGLLGEINQTVDSREEVLAAWKRQLERLWNEVN...............
gi|81708722|sp|Q7NEZ9|Q7NEZ9_GLOVI   GFQQAFEDLADAKLRQIVVALQDQAKTLEEALQLLERLYDEGVLGTLQSTREDRELTLQVWRLKLTRLWHEADS.P............
gi|298346581|ref|YP_003719268.1|      .......................................................EQVRCALESSEDFFEQLEQ.QL....PNAMPQP
gi|260576430|ref|ZP_05844420.1|       .......................................................DSLDIWERLVGDFKKHIN...............
gi|296129402|ref|YP_003636652.1|      .......................................................QILFCGLSTGSMLFDALDE.RY....PDPAGAA
gi|254464399|ref|ZP_05077810.1|       .......................................................QTVSLWTRAMQDFEEFIG...W...........
gi|269795609|ref|YP_003315064.1|      .......................................................RLLACGLSTSAMLFDTLDG.LY....PDPAAVA
gi|167648377|ref|YP_001686040.1|      .......................................................DDFGVWTGMLGDFTKFID...............
gi|302865994|ref|YP_003834631.1|      .......................................................RLVDCGVTTGCQLAAAVDE.LA....GGTL...
gi|254474961|ref|ZP_05088347.1|       .......................................................ETLGIWSRIVQDFETLID...W...........
gi|238063407|ref|ZP_04608116.1|       .......................................................QLLDCGISTGCQLAAAAGQ.LA....GGVR...
gi|227495918|ref|ZP_03926229.1|       .......................................................LAVHCAMTTGLRFFDHLTT.EI....FPEEWKV
gi|269219692|ref|ZP_06163546.1|       .......................................................QHIACAISTGTQFFEYLNE.TF....PQEWEAY
gi|145593940|ref|YP_001158237.1|      .......................................................RLLDRGITTGCQLAADVAR.LP....GGVR...
gi|269976471|ref|ZP_06183456.1|       .......................................................EHLHCALESSEDFFEKLGE.KF....PQFDEGT
gi|217969472|ref|YP_002354706.1|      .......................................................DSFNWWTKALKEYKKVVLE..............
gi|399991304|ref|YP_006571544.1|      .......................................................ELLGYWSRIVQEFEEFIN...W...........
gi|256832278|ref|YP_003161005.1|      .......................................................TTLACAIQTTTQFFDELDR.HI....PDPALHR
gi|225022571|ref|ZP_03711763.1|       .......................................................QSLDCGIATSMSFFESLDK.KF....PDTRFA.
gi|126738030|ref|ZP_01753751.1|       .......................................................SMVSLWARSMSEFEDMID...W...........
gi|254294753|ref|YP_003060776.1|      .......................................................DTLDIWRRLLETYETEFAA.QA....PA.....
gi|149184640|ref|ZP_01862958.1|       .......................................................DNLLYWESVMVGFAEFSFS.KD....N......
gi|257068239|ref|YP_003154494.1|      .......................................................SAITCGLSTVIAFFHSLEE.RL....GAEIDE.
gi|229820926|ref|YP_002882452.1|      .......................................................HHVQLGLMSGMMVFDHLDE.MF....PDPVALQ
gi|227494902|ref|ZP_03925218.1|       .......................................................MQLQCAVTTSRQFFEELEA.AL....D......
gi|220934858|ref|YP_002513757.1|      .......................................................DWLQTFNMLIDMFVREVVH.GQ....GVDDSK.
gi|291295614|ref|YP_003507012.1|      .......................................................DMCNTVMLAAHQFVMELNP.GR....GQGVAE.
gi|296130820|ref|YP_003638070.1|      .......................................................LQLACGISTALQLFEHLDE.QY....PAEAAAA
gi|84685465|ref|ZP_01013363.1|        .......................................................ALLELWLDSVVLFEDHIK...W...........
gi|229820927|ref|YP_002882453.1|      .......................................................ETITLALSVTAQFFAGLEA.SV....PPAREE.
gi|227496193|ref|ZP_03926499.1|       .......................................................QQVACAMGTIADAFADVRS.RL....ALRADNL
gi|154507942|ref|ZP_02043584.1|       .......................................................QQVMCALATTLQLFRALEE.RY....GLDDDEG
gi|86137245|ref|ZP_01055823.1|        .......................................................TTVGLWSRLMGDFEELID...Y...........
gi|227494903|ref|ZP_03925219.1|       .......................................................LQLHCAIATSLDAIRTGYA.EL....GISLEEE
gi|152982702|ref|YP_001354063.1|      .......................................................VSLQLWTQMMGQFFLHLRN.FK....PESVPA.
gi|119486978|ref|ZP_01620850.1|       .......................................................RIITVVATIADDNDDDLQA.EF....G......
gi|145596051|ref|YP_001160348.1|      .......................................................EMLDCGAAVATSFVDWLHD.EG....AL.....
gi|220903645|ref|YP_002478957.1|      .......................................................EIFDLWKTSLRHLSEVIAA.QS....KKNPS..
gi|297566041|ref|YP_003685013.1|      .......................................................TFVREFEDSCNLFFSYIRM.ID....AELV...
gi|227496194|ref|ZP_03926500.1|       .......................................................QHVDCAIATVNGLFADLNE.VL....G......
gi|119385461|ref|YP_916517.1|         .......................................................DALDTWRIVLSDFRDFID...............
gi|183220878|ref|YP_001838874.1|      .......................................................EFLQKFQILNSQFSTLLIL.AE...........
gi|291302096|ref|YP_003513374.1|      .......................................................EFVDCAAAVSGAYIDWLHG.EA....GI.....
gi|387905553|ref|YP_006335891.1|      .......................................................STVELWDRLIQEFLLHLRD.RP....TLAEQE.
gi|238927566|ref|ZP_04659326.1|       .......................................................AMMAVGMRAVEQVHESLIG.LC....ERGHRP.
gi|322420651|ref|YP_004199874.1|      .......................................................DMVALIFRAMKEEHPRLLK.VV...........
gi|225629038|ref|ZP_03787072.1|       .......................................................YVLAIWADLIQAFLVHLRD.DQ....NILLRY.
gi|227495837|ref|ZP_03926148.1|       .......................................................SFVFTSCRLIVALMREAEN.LF....PDPLRGN
gi|291295390|ref|YP_003506788.1|      .......................................................AFVHDFGDQVTLFFSYLRM.ID....AGLV...
gi|302381558|ref|YP_003817381.1|      .......................................................DPIQVWRSLSNDLGRTVTV.AA....NAAPAV.
gi|229589918|ref|YP_002872037.1|      .......................................................SQLELWDRLLQEFIVYLRE.YS....QQTAQL.
gi|229917768|ref|YP_002886414.1|      .......................................................QHSFMMFQAADEVNEYMQK.LY....TQV....
gi|304320687|ref|YP_003854330.1|      .......................................................VSIANYLHMASTFDRHLFE.AT....QTVQKE.
gi|154508096|ref|ZP_02043738.1|       .......................................................DAISSGISTGIAAIQYVKE.SA....SEALGL.
gi|172058831|ref|YP_001815291.1|      .......................................................GHSVMMFQASDEVFAYLTK.LY....DQV....
gi|85705365|ref|ZP_01036464.1|        .......................................................SGLGQVVNLAQSYGTLYSG.GA....LHYGGG.
gi|119512017|ref|ZP_01631112.1|       .......................................................RLVTIVATIADENDEVLQA.EF....GL.....
gi|256397448|ref|YP_003119012.1|      .......................................................GACAQIGRIADEYDDRFKE.LV....GAGHTVF
gi|94985189|ref|YP_604553.1|          .......................................................TLFGGVLMHFQDDYAALEG.Y......VPGPQL
gi|238927565|ref|ZP_04659325.1|       .......................................................SMVGVGLQVLEEVYPALMK.LR....W......
gi|304320540|ref|YP_003854183.1|      .......................................................ERLKRWPELIRTFRDQMRN.AQ....TG.....
gi|163846985|ref|YP_001635029.1|      .......................................................FATRTFIDHVRAALAKDET.DV....IA.....
gi|121606820|ref|YP_984149.1|         .......................................................RACRQFSGGISLAIDEFDP.EY....KILMRL.
gi|121583407|ref|YP_973838.1|         .......................................................RSCRQFARNLEKGLELVDP.EN....DFVLPP.
gi|167856646|ref|ZP_02479340.1|       .......................................................DTLTRIFVGAYLFASEMKK.E............
gi|283780877|ref|YP_003371632.1|      .......................................................KFLNYYFSQLAPFYKEVCE..............
gi|85708893|ref|ZP_01039959.1|        .......................................................AQMVVFGQNITTLSRALIE.LE....N......
gi|304321485|ref|YP_003855128.1|      .......................................................VNLRAFASSTQKWESVAGR.GS....SRVVSF.
gi|304321021|ref|YP_003854664.1|      .......................................................FTFGLFLEDANTIQEMVIE.QS....GPGPFE.
consensus>70                          ........................................................................................
```

	160
E.amylovora-AmyR	MITSHSLL.......H..
D.radiodurans-DR1245	....QQP.......Q........A..
S.elongatus-T110839	....
E.coli-YbjN	.VTENYFL.......H..
gi|123253281|sp|Q1GTJ6|Q1GTJ6_SPHAL	..................E...
gi|123129053|sp|Q0C3X0|Q0C3X0_HYPNA	.E..AEYEDDEDYFYED..W
gi|123323499|sp|Q0C3W9|Q0C3W9_HYPNA	.E..DEYDGD...W
gi|122544362|sp|Q2NA07|Q2NA07_9SPHN	....
gi|123736226|sp|Q2G8G7|Q2G8G7_NOVAD	....K
gi|122459425|sp|Q1YFZ1|Q1YFZ1_9RHIZ	..................L...
gi|122351524|sp|Q0G7X7|Q0G7X7_9RHIZ	....
gi|123004239|sp|Q2IXX8|Q2IXX8_RHOP2	.T..NANGGGAAASPAVEPGPSPAATM..Q
gi|122476043|sp|Q213H6|Q213H6_RHOPB	.N..TPSPGAALNHQPKVASPGDDAGESASS.VSA..A
gi|122295500|sp|Q07KZ5|Q07KZ5_RHOPA	.S..DAAARPENQAPQSQEPSRDRTAQLAAS.ALI..Q
gi|122403190|sp|Q1I9R8|Q1I9R8_9PSED	.T..GSEAVSA..S
gi|122257709|sp|Q02HI9|Q02HI9_PSEAE	.Q..GMAQDAGQPVDGE..A
gi|81415193|sp|Q745S9|Q745S9_THET2	..................E...
gi|81596955|sp|Q5N656|Q5N656_SYNP6	....
gi|123557011|sp|Q31PB3|Q31PB3_SYNP7	....
gi|81709081|sp|Q7NG79|Q7NG79_GLOVI	.P...A
gi|123506214|sp|Q2JU30|Q2JU30_SYNJA	....
gi|123556836|sp|Q31NC7|Q31NC7_SYNP7	....
gi|81670584|sp|P73244|P73244_SYNY3	....
gi|122663130|sp|Q4C8F6|Q4C8F6_CROWT	....K
gi|81771933|sp|Q8YVL5|Q8YVL5_ANASP	....A
gi|123351931|sp|Q10XZ9|Q10XZ9_TRIEI	....Q
gi|81596544|sp|Q5N4K3|Q5N4K3_SYNP6	................SW.EQ..S
gi|123608251|sp|Q3M567|Q3M567_ANAVT	....S
gi|81708722|sp|Q7NEZ9|Q7NEZ9_GLOVI	................Q..N
gi|298346581|ref|YP_003719268.1|	....DEDS...E
gi|260576430|ref|ZP_05844420.1|	..................F...
gi|296129402|ref|YP_003636652.1|	....P
gi|254464399|ref|ZP_05077810.1|	....
gi|269795609|ref|YP_003315064.1|	....P
gi|167648377|ref|YP_001686040.1|	....W
gi|302865994|ref|YP_003834631.1|	....R
gi|254474961|ref|ZP_05088347.1|	....
gi|238063407|ref|ZP_04608116.1|	....P
gi|227495918|ref|ZP_03926229.1|	....FEAEQAEREALG...E
gi|269219692|ref|ZP_06163546.1|	....QVQAEAQAIAVAEAQAVAEAEADARYN.FKGD........SDRPEEDLTRPDSLALPEPPFHDSSYQPQAEEDDSRKSED
gi|145593940|ref|YP_001158237.1|	....S
gi|269976471|ref|ZP_06183456.1|	....PDEE...A
gi|217969472|ref|YP_002354706.1|	..................E...
gi|399991304|ref|YP_006571544.1|	....
gi|256832278|ref|YP_003161005.1|	....P
gi|225022571|ref|ZP_03711763.1|	.......................Q..
gi|126738030|ref|ZP_01753751.1|	....
gi|254294753|ref|YP_003060776.1|	....D
gi|149184640|ref|ZP_01862958.1|	....D
gi|257068239|ref|YP_003154494.1|	.L........DS..
gi|229820926|ref|YP_002882452.1|	....PD.G...A
gi|227494902|ref|ZP_03925218.1|	....A
gi|220934858|ref|YP_002513757.1|	.P..VTPRRDRELRAGISPTV..G
gi|291295614|ref|YP_003507012.1|	.M..QDLFRTLLERLRERR...
gi|296130820|ref|YP_003638070.1|	....KAAQEQ...D
gi|84685465|ref|ZP_01013363.1|	....
gi|229820927|ref|YP_002882453.1|	....S
gi|227496193|ref|ZP_03926499.1|	....NESQ...W
gi|154507942|ref|ZP_02043584.1|	....HGPAGGHQRGFDGPAWLPES..
gi|86137245|ref|ZP_01055823.1|	....
gi|227494903|ref|ZP_03925219.1|	....E..A
gi|152982702|ref|YP_001354063.1|	.T..DAAAAVTADESVA..A
gi|119486978|ref|ZP_01620850.1|	....Q
gi|145596051|ref|YP_001160348.1|	............L...
gi|220903645|ref|YP_002478957.1|	....
gi|297566041|ref|YP_003685013.1|	...............E..
gi|227496194|ref|ZP_03926500.1|	....R
gi|119385461|ref|YP_916517.1|	..................W...
gi|183220878|ref|YP_001838874.1|	....
gi|291302096|ref|YP_003513374.1|	............L...
gi|387905553|ref|YP_006335891.1|	.A..AARAADADRPDAPAAVTASSGAAVP..S
gi|238927566|ref|ZP_04659326.1|	.R..ARAS.......E..
gi|322420651|ref|YP_004199874.1|	............Q...
gi|225629038|ref|ZP_03787072.1|	.K..RRKQAEQRIQILATCTSGKLRIQPI..W
gi|227495837|ref|ZP_03926148.1|	....LE...P
gi|291295390|ref|YP_003506788.1|	...............E..
gi|302381558|ref|YP_003817381.1|	.P..PA...Q
gi|229589918|ref|YP_002872037.1|	.Q..AAAVGEEAPV...L
gi|229917768|ref|YP_002886414.1|	...............Q..
gi|304320687|ref|YP_003854330.1|	.A..LDTDLAANGLNMQGILADGDLAAQIGL.SPSS........HQVSFEAMTDVVDTFIKADGLAPGRIVN......AVTGKR
gi|154508096|ref|ZP_02043738.1|	.G..ESGR..............E...
gi|172058831|ref|YP_001815291.1|	...............Q..
gi|85705365|ref|ZP_01036464.1|	.D..SGALQRALIDELLKKGEGI...
gi|119512017|ref|ZP_01631112.1|	....A
gi|256397448|ref|YP_003119012.1|	....DGDPGDDDSVEV..
gi|94985189|ref|YP_604553.1|	....QQP.......Q........A..
gi|238927565|ref|ZP_04659325.1|	...............T..
gi|304320540|ref|YP_003854183.1|	....S
gi|163846985|ref|YP_001635029.1|	....
gi|121606820|ref|YP_984149.1|	.M..KTEPESESSENE..
gi|121583407|ref|YP_973838.1|	.G..KIEPEDEETP..
gi|167856646|ref|ZP_02479340.1|	....
gi|283780877|ref|YP_003371632.1|	....E
gi|85708893|ref|ZP_01039959.1|	......................G...
gi|304321485|ref|YP_003855128.1|	.T..PLVPDGPP...VSEGKEDTHLKEATAIAAPADPADPHLVTLSPGQTSSDSARGNALMIKQIAFEPTLS......PKE..R
gi|304321021|ref|YP_003854664.1|	.V..SVPPDERFPRLASAVAATGWDVSRLVA.RFGP........LSPERASPQVPFPSITGFGLAVQE...............D
consensus>70	....
